# Novel Chiral Bis-Phosphoramides as Organocatalysts for Tetrachlorosilane-Mediated Reactions

**DOI:** 10.3390/molecules22122181

**Published:** 2017-12-08

**Authors:** Sergio Rossi, Marco Ziliani, Rita Annunziata, Maurizio Benaglia

**Affiliations:** Dipartimento di Chimica, Università Degli Studi di Milano, via Golgi 19, 20133 Milan, Italy; marco.ziliani@studenti.unimi.it (M.Z.); rita.annunziata@unimi.it (R.A.)

**Keywords:** phosphoroamides, metal-free catalysis, hypervalent silicon, allylation, self-assembly

## Abstract

The formation of novel chiral bidentate phosphoroamides structures able to promote Lewis base-catalyzed Lewis acid-mediated reactions was investigated. Two different classes of phosphoroamides were synthetized: the first class presents a phthalic acid/primary diamine moiety, designed with the aim to perform a self-assembly recognition process through hydrogen bonds; the second one is characterized by the presence of two phosphoroamides as side arms connected to a central pyridine unit, able to chelate SiCl_4_ in a 2:1 adduct. These species were tested as organocatalysts in the stereoselective allylation of benzaldehyde and a few other aromatic aldehydes with allyl tributyltin in the presence of SiCl_4_ with good results. NMR studies confirm that only pyridine-based phosphoroamides effectively coordinate tetrachlorosilane and may lead to the generation of a self-assembled entity that would act as a promoter of the reaction. Although further work is necessary to clarify and confirm the formation of the hypothesized adduct, the study lays the foundation for the design and the synthesis of chiral supramolecular organocatalysts.

## 1. Introduction

**Figure 1:**
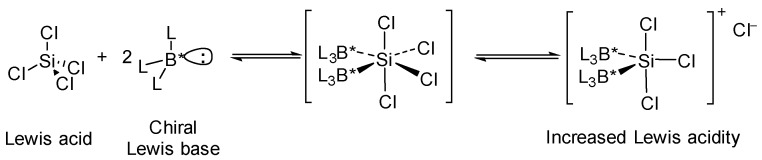
Formation of hypervalent silicon species.

In recent years, chiral phosphoramides have attracted increasing attention due to their ability to promote stereoselective reactions. The most important examples of mono- and bis-phosphoroamides used in stereoselective processes were developed by Denmark [1]. These compounds have found extensive application as catalysts in the so called “Lewis base-catalyzed Lewis acid-mediated reactions” [2] where the combination of chlorosilanes with enantiomerically pure phosphoroamides or phosphinoxides [3] leads to an in situ formation of a new hypervalent (cationic) silicon species with increased acidity at the silicon center that acts as chiral Lewis acid and efficiently promotes highly stereoselective reactions (Figure 1) [4,5,6].

**Figure 2:**
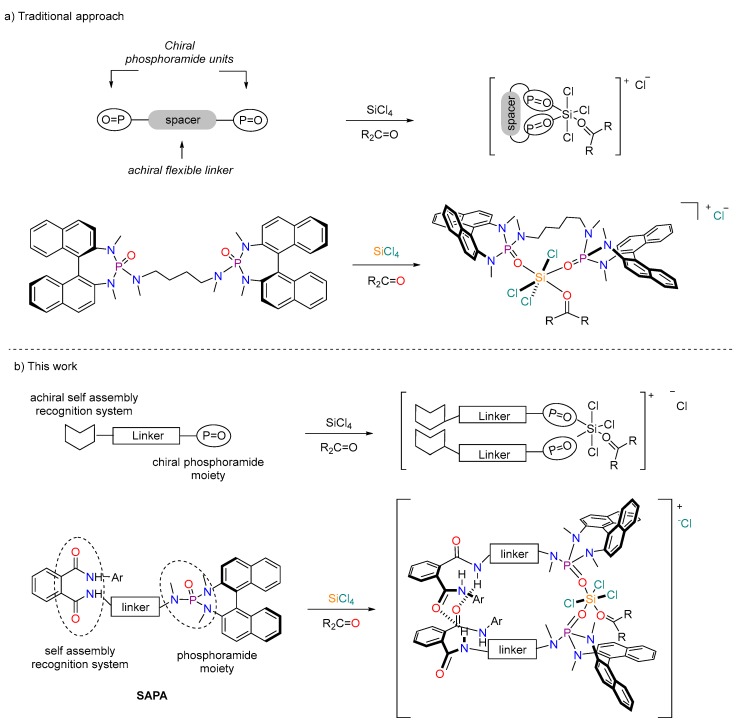
(**a**) Traditional approach for the activation of SiCl_4_ using bis-phosphoramides; (**b**) Design and the synthesis of SAPAs Lewis bases.

In this process, a weak Lewis acidic species could be activated (converted into a stronger acid) upon binding of a chiral Lewis base that is responsible for the control of the stereochemical outcome of the reaction. *C*_2_ symmetric bis-phosphoroamides have been preferentially used, due to the possibility of generating a highly defined space around the silicon atom that can better guarantee high stereocontrol. The conformational restriction provided by the linkage, if well optimized, favorably influences the selectivity and the activity of the catalyst [2,7]. Generally, the structure of these bisdentate phosphoroamides presents two distinct Lewis basic moieties connected through a covalent achiral linker, and their synthesis requires a long and not trivial sequence of synthetic steps. In order to overcome these limitations, a possible solution consists of the synthesis of more easily accessible chiral bis-phosphoroamides, where the covalent tether between the two phosphoramide subunits could be replaced with a non-covalent interaction. In this way, a new in situ bisdentate supramolecular phosphoramide could be obtained and employed as an organocatalyst in Lewis base-catalyzed Lewis acid-mediated reactions. In this work, we report the design and the synthesis of a small library of new, potentially Self-Assembled PhosphorAmides derived Lewis bases (SAPAs) where the covalent linker connecting the two phosphoramide units is replaced by a reversible linker, generated with non-covalent interactions by hydrogen bonding (Figure 2).

There are several strategies to form bidentate ligands by self-assembly recognition of monodentate ligands [8], but the self-assembly between two *homo*-monodentate ligands through non-covalent complementary interactions (one component assembly) represents the easier one since only *homo*-complexes can be formed. The first example describing the selective and reversible assembly of a metal-coordinated two equal monodentate ligands was reported by Breit and Seiche in 2003 [9,10]. The known property of tautomeric pair 2-pyridone/2-hydroxypyridine to dimerize through hydrogen bonds in aprotic solvents was exploited in the regioselective hydroformylation of terminal alkenes to linear aldehydes. Following this strategy, Reek reported the use of MetamorPhos as an adaptive supramolecular ligand for asymmetric hydrogenation [11]. In 2005, Love [12] described the ability of urea-phosphine ligands to form well-defined chelates at palladium and rhodium in the presence of a chloride ion, and few years later, van Leeuwen and Reek [13,14] reported a systematic study of urea-functionalized phosphorous ligands (UREAphos) and palladium complexes that self-associate by hydrogen bond formation. In 2006, Ding and co-workers reported the application of a new class of phosphoramidite ligands for rhodium to promote the enantioselective hydrogenation of several α,β-unsaturated esters [15], and two years later, Breit developed the use of *meta*-carboxypeptidyl-substituted triarylphosphines and phosphites to construct PhanePhos-like structures by means of an inter-ligand helical hydrogen-bonding network [16]. More recently, Gennari reported two novel classes of chiral monodentate phosphite ligands, named PhthalaPhos [17,18] and BenzaPhos [19], which contain, respectively, a phthalic acid primary diamine moiety and a benzamide moiety (that presents both donor and acceptor hydrogen-bonding properties) connected to a chiral 1,1′-binaphthol-derived phosphite moiety, responsible for the coordination of the supramolecular ligand to the metal center. These ligands were satisfactorily employed in the rhodium-catalyzed hydrogenation of acetamidoacrilates and acetamides. 

Supramolecular constructs for the synthesis of ligands are well documented in transition-metal-based catalysis [8,20,21,22,23,24,25,26,27] but only a few examples have been reported in an organocatalytic approach [28].

For this reason, we decided to investigate the possibility of employing supramolecular bisphosphoramide adducts in organocatalysis. In our hypothesis, the role of the metal as a coordinating center for the bisdentate ligand could be replaced by the presence of a silicon atom, in its hypervalent state (Figure 2).

## 2. Results and Discussion

Following this approach, we developed a strategy for the synthesis of a simple, low-weight and relatively inexpensive phosphoramide-based catalyst, where the covalent tether was replaced by non-covalent interactions, as H-bonds. The structure of the catalyst can be subdivided into three different parts: a phosphorous-based catalytic center, responsible for the interaction with the silicon atom; a self-recognition moiety, responsible of the aggregation of two catalyst units, and a linker to distance these units, avoiding interferences between coordination and recognition processes. 

**Scheme 1:**
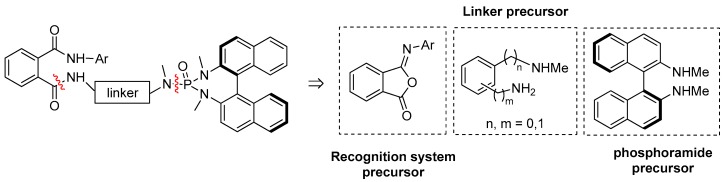
Retrosynthetic approach for the synthesis of SAPA derivates.

Inspired by previously reported examples, we decided to start our investigation by employing the phthalic acid primary diamide moiety as a recognition system [17,18,19]. Binaphthyl diamine-derived phosphoramide was selected as the catalytic center, since this type of structure has proven to be an efficient organocatalyst in many type of reactions involving the generation of hypervalent trichlorosilyl species [1,2]. As the Lewis base and the self-assembly recognition system are fixed, the subsequent step consisted of the evaluation of the correct linker between these two functionalities. Based on literature [29,30] and previous experiences in our group, the phosphorous atom needs to be connected to three nitrogen atoms bearing an alkyl substituent to generate stable compounds. For these reasons, different *N*-methylsubstituted anilines were selected as target linkers (Scheme 1).

The synthesis of the optimal linker necessary to connect the two subunits responsible for the recognition process and the catalytic process requires particular attention. The geometry of the linkers is extremely important because their conformational restriction influences the orientation of the two subunits as well as the selectivity and reactivity of the final self-assembled catalyst. Since the linker bears two amino-functionalities with similar reactivity, the primary amino group (employed for the connection of the recognition system), was generated through the reduction of nitro or nitrile groups.

**Scheme 2:**
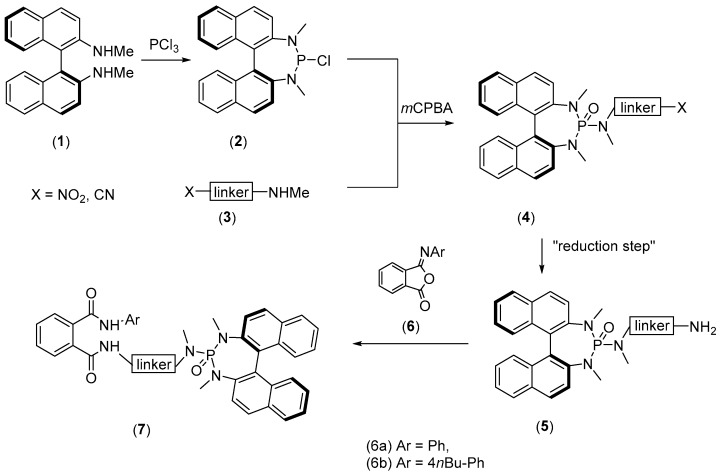
General synthesis of SAPAs derivatives.

**Scheme 3:**
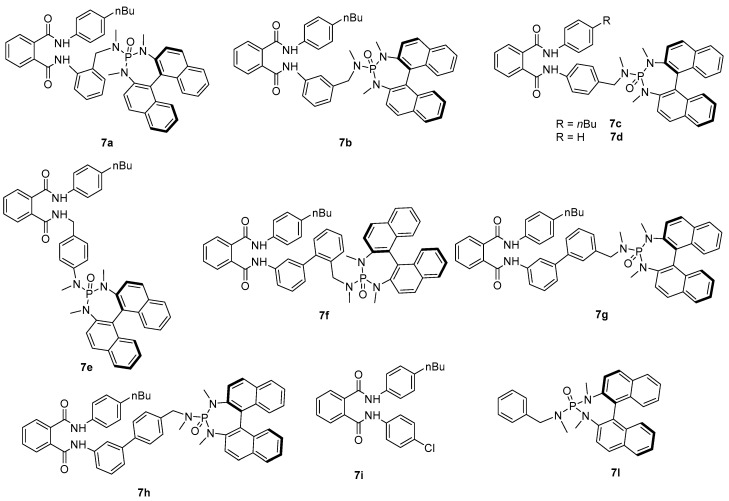
Library of SAPAs derivatives.

The final ligand could be then synthetized starting from the three general precursors reported in Scheme 2. First, chloro-diamino-phosphine (**2**) generated in situ by the reaction of (*S*)-*N*,*N*′-dimethyl-[1,1′-binaphthalene]-2,2′-diamine (**1**) with PCl_3_ [31] was treated with aryl methylamines (**3**) to synthesize phosphoroamides (**4**). At this point, the nitro- or the cyano-group was reduced to amine, forming compounds (**5**), which were converted in the desired ligands (**6**) by reaction with phthalic anhydrides. A small collection of SAPAs derivatives was then synthesized with good yields. These compounds have been reported in Scheme 3 (see supporting information for further details). 

SAPAs **7a**–**d** derive from *orto- meta* and *para-*substituted ((methylamino)methyl) anilines, while compound 7**e** features 4-(aminomethyl)-*N*-methylaniline as a linker. In compounds **7f**–**h** the recognition part is separated from the phosphoroamide unit by the presence of a substituted biphenyl moiety. Furthermore, for control experiments, compound 7**h** (no possibility of self-assembly interaction) and compound 7**i** (no catalytic active site) were synthesized.

All the new ligands were employed as organocatalysts in the enantioselective allylation of benzaldehyde with allyl tributyltin in the presence of SiCl_4_ (Table 1). One equivalent of benzaldehyde was reacted with 1.2 equivalents of allyl tributyltin in the presence of 2 equivalents of SiCl_4_ in dry DCM, using 10 mol % of the monodentate catalyst under a nitrogen atmosphere [32]. Results are shown in Table 1.

As expected, the phosphoramide unit is necessary to promote the reaction (entry 1). Phosphoramide (**7c**) was able to catalyze the reaction with the formation of the product in 73% yield and 70% enantiomeric excess. High dilution conditions were detrimental in terms of yield but did not influence the stereochemical outcome of the reaction (entry 5). The butyl chain had no influence on the reactivity or on the stereoselection (entry 6). Catalyst (**7b**) led to the formation of allyl alcohol (**8a**) with lower yield than the catalyst (**7a**), but with better enantioselectivity (entry 2 vs. entry 3). Catalyst (**7e**) was extremely reactive but did not exert any type of stereocontrol on the process (entry 7). Catalysts (**7g**) and (**7f**) presented a good chemical efficiency but led to the formation of the product with modest stereoselection (entries 8 and 9). By using catalyst **7l**, unable to be involved in a self-assembly recognition process, it was possible to observe the formation of the desired product with good yield and stereoselectivity. 

On the basis of these results, and in order to understand if a real self-assembly recognition process took place, we performed ^1^H-, ^31^P- and ^29^Si-NMR analysis on the catalysts in the presence and absence of SiCl_4_. We first studied the dependence of the NH chemical shifts on the concentration of the ligand **7c** in the 0.45–30 mM range; we determined that the self-aggregation of ligand **7c** due to H-bonding became not significant at 3.75 mM concentration. Thus, the NMR experiments on ligands **7** and SiCl_4_ were conducted at 3.75 mM in CD_2_Cl_2_. 

**Scheme 4:**
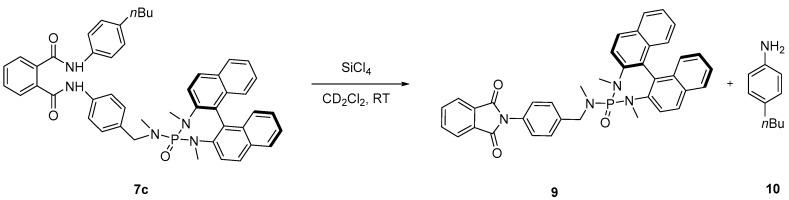
Degradation of chiral ligand **7c**.

Different ratios of (SiCl_4_):(phosphoroamide ligand **7c**) were investigated at −50 °C by NMR. Unfortunately, no chemical shift variation was observed in the ^31^P spectra of the chiral ligand after the addition of SiCl_4_. ^29^Si spectra showed two signals only: −19 ppm, due to free SiCl_4_ and one at −46 ppm, corresponding to a tetravalent silicon atom bounded with oxygen atoms. No signals were observed in the range from −200 to −210 ppm, expected for hexacoordinated silicon species [2,33,34]. Moreover, raising the temperature from −50 °C to RT, it was possible to observe a partial degradation of the ligand **7c** corresponding to the elimination of 4-*n*Bu-aniline **10** with consequent formation of a phthalimide derivative **9**. This could be explained by the coordination of SiCl_4_ to the amide functionality that becomes sensitive to the nucleophilic attack from a nitrogen atom (Scheme 4).

Since these results clearly indicated that no supramolecular structure was formed and that the catalytic activity was formally expressed by a single monomeric ligand, we started to investigate different recognition systems. Since the use of metal as an aggregation center is not compatible with LB-catalyzed LA-mediated reactions, we focused our attention on the use of SiCl_4_ itself as an aggregating agent for the formation of the supramolecular structure. 

**Scheme 5:**
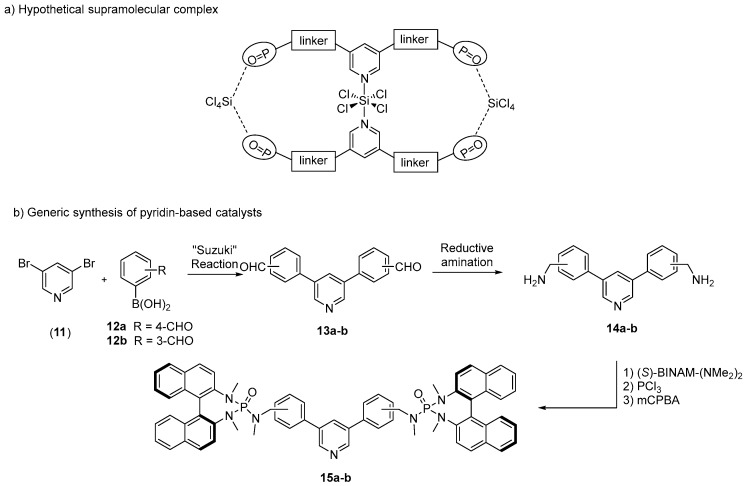
(**a**) Hypothetical supramolecular complex; (**b**) Synthesis of compounds 15a-b.

It is known that SiX_4_ compounds can generate hexacoordinated complexes in the presence of 1,1-phenanthroline, 2,2′-bipyridine and pyridines [35,36,37,38,39,40,41,42]. Among them, only pyridines are able to generate *trans*-complexes with a 2:1 pyridine:Si ratio; therefore, we decided to synthesize a recognition system (Scheme 5a). The capability of SiCl_4_ to form a Py_2_:SiCl_4_ adduct was also confirmed by our preliminary ^29^Si-NMR experiments, where one single signal was observed at −178 ppm at 210 K in CD_2_Cl_2_ (compatible with the bis-*N*-coordinated Si(Halogen)_4_ species). 

New catalysts were then designed and synthesized in three steps only according to the general procedure reported in Scheme 5b. The synthesis involved a Suzuki coupling between 3,5-dibromopyridine (**11**) and carboxyphenylboronic acid **12**, leading to the formation of compound **12**. This compound was then subjected to reductive amination, yielding diamine (**14**), which, upon treatment with (*S*)-1,1′-Binaphthyl-2,2′-diamine **1,** allowed us to obtain C*_2_* chiral ligands **15** with good yields. With this protocol, two new catalysts were synthesized (**15a**,**b**), in 52% and 56% yield respectively, and they were investigated as organocatalysts in the enantioselective allyltributyltin addition to aromatic aldehydes promoted by SiCl_4_ (Table 2). 

Catalyst **15a** allowed us to obtain the desired product **8a** in 71% yield and 61% ee, while catalyst **15b** generated allylic alcohol in lower yield and poor enantiomeric excess, indicating that the *meta* substitution resulted in an unfavorable conformation adopted by the ligand when coordinated with tetrachlorosilane. Product **8b** derived from *p*-chlorobenzaldeyde was obtained in 83% yield and a comparable level of enantioselection, but when electron-donating substituents were present (entry 4), the yield and the stereoselection decreased considerably.

Although further work is necessary to clarify and to confirm the formation of the hypothesized self-assembled adduct, these preliminary results are encouraging and lay the foundation for the study of chiral supramolecular organocatalysts as efficient promoters of stereoselective reactions.

## 3. Materials and Methods

^1^H-NMR, ^13^C-NMR and ^29^Si-NMR spectra were recorded with instruments at 300 MHz (Bruker F300, Billerica, MA, USA) or 500 MHz (Bruker ADVANCE 500 or 600). Proton chemical shifts are reported in ppm (δ) with the solvent reference relative to tetramethylsilane (TMS) employed as the internal standard (CDCl_3_ = δ 7.26 ppm). ^13^C-NMR spectra were recorded operating at 75 MHz, 125 MHz or 192.5 MHz, with complete proton decoupling. Carbon chemical shifts are reported in ppm (δ) relative to TMS with the respective solvent resonance as the internal standard (CDCl_3_, δ = 77.0 ppm). ^29^Si-NMR spectra were recorded operating at 99 MHz; chemical shifts are reported in ppm (δ) relative to TMS. ^31^P spectra were recorded at 121.4 or 202.4 MHz and were referenced to phosphoric acid (H_3_PO_4_) at 0.0 ppm. HPLC analysis was performed on an Agilent Instrument Series 1100 or 1200 series on chiral stationary phase. Purification of the products was performed by column chromatography on silica gel (230–400 mesh ASTM, Merck, Kenilworth, NJ, USA). All the solvents used are commercially available (≥99%, chromatographic grade, purchased from Sigma Aldrich, St. Louis, MO, USA) and stored under nitrogen over molecular sieves (bottles with crown caps). Reactions were monitored by analytical thin-layer chromatography (TLC) using silica gel 60 F_254_ pre-coated glass plates and visualized using UV light.

### 3.1. General Procedure for the Synthesis of Nitro-Phosphoroamides *(**4**)*

*N,N′*-dimethyl-1,1′-binaphthyl-2,2′-diamine (**1**) (1 eq., 3.20 mmol, 1.0 g) and Et_3_N (3 eq., 9.6 mmol, 1.33 mL) were dissolved in dry THF (32 mL). The homogeneous mixture was cooled to 0 °C then PCl_3_ (3 eq., 9.60 mmol, 0.84 mL) was added dropwise via a syringe whereupon a colorless precipitate formed immediately. The reaction mixture was stirred at 0 °C for 1.5 h and was then allowed to warm to room temperature and stirred for another 3 h. The volatiles were removed under high vacuum (room temperature, 0.5 mmHg) and Et_2_O (30.0 mL) was added via syringe, then the mixture was stirred for 5 min. Subsequently, the supernatant was canula-filtered into another round bottom flask. The remaining precipitate in the reaction flask was washed again with Et_2_O (30 mL) and filtered (twice). The volatiles were removed under high vacuum (room temperature, 0.5 mmHg) to yield a light yellow solid. The solid was then dried for 12 h at reduced pressure (room temperature, 0.5 mmHg) to yield a white solid foam (**2**). Dry CH_2_Cl_2_ (40 mL) was added via syringe and the mixture was cooled to 0 °C. To this solution, a mixture of Et_3_N (2 eq., 6.40 mmol, 0.98 mL) and the desired methylamine (**3**) (1.2 eq., 3.84 mmol) dissolved in dry CH_2_Cl_2_ (4 mL) were added. The reaction mixture was allowed to warm to room temperature and stirred for 20 h. A solution of mCPBA (70%) (1.5 eq., 4.80 mmol, 1.18 g) dissolved in 2 mL of THF was then added and the mixture was stirred for 20 h. After quenching with 15 mL of NH_4_Cl saturated aqueous solution, the phases were separated and the aqueous layer was washed with CH_2_Cl_2_ (10 mL). The combined organic extracts were washed with brine, dried over Na_2_SO_4_, filtered and concentrated by rotary evaporation. The crude residue was purified by silica gel flash chromatography using ethyl acetate (100%) as an eluent to yield phosphoroamides with different yields.

### 3.2. General Procedure for the Synthesis of SAPAs Catalyst *(**7**)*

The desired phosphoroamide (**5**) (1 eq., 0.1 mmol) and the desired phthalisoimide (**6**) (3 eq., 0.3 mmol) were dissolved in dry THF (2 mL). The homogeneous mixture was stirred at RT for 48 h, then quenched with 5.0 mL of HCl 5%. The phases, diluted with ethyl acetate were separated and the obtained aqueous layer was washed with ethyl acetate (5.0 mL). The combined organic extracts were washed with brine, dried over Na_2_SO_4_, filtered and the filtrate was concentrated by rotary evaporation. The residue was purified by silica gel flash chromatography using different mixtures furnishing the desired product.

### 3.3. General Procedure for Allylation of Benzaldehyde

Phosphoramide catalyst (0.1 eq. or 0.05 eq.) was dissolved in CH_2_Cl_2_ (1.5 mL) under N_2_. Allyltributyltin (1.2 eq., 0.54 mmol, 169 μL) was added to this solution and the resulting mixture was cooled to −78 °C (bath temperature). Then, freshly distilled SiCl_4_ (2 eq., 0.9 mmol, 104 μL) was added followed by the aldehyde (1 eq., 0.45 mmol). The resulting mixture was stirred at −78 °C (bath temperature) for 6 h whereupon the cold reaction mixture was rapidly poured into a stirring solution of 1/1 sat. aq. KF/1.0 M KH_2_PO_4_ (5 mL). This biphasic mixture was stirred vigorously for 12 h, then, diluted with CH_2_Cl_2_ (10 mL); the layers were separated and the aqueous one was washed with CH_2_Cl_2_ (3 × 10 mL). The combined organic extracts were dried over Na_2_SO_4_, filtered and the filtrate was concentrated by rotary evaporation under vacuum. The residue was purified by silica gel flash chromatography using hexane/ethyl acetate (9:1, *v*/*v*) as an eluent with a plug of solid anhydrous KF (15 mm) on the top of the column . The product-containing fractions were combined, and the solvent was removed by rotary evaporation under vacuum to yield the desired allylic alcohol.

## 4. Conclusions

In conclusion, in this work, we investigated the synthesis of new chiral bidentate phosphoroamides as promoters of Lewis base-catalyzed Lewis acid mediated reactions. A library of different mono- and bis-phosphoramides was synthetized in good yields. These molecules were characterized by the presence of three different designed subunits (a recognition moiety, a linker and a catalytic center) in order to favor a recognition process through reversible interactions for the synthesis of a new bisphosphoroamides adduct. While the use of a phthalic acid primary diamine moiety for a self-assembly recognition process through hydrogen bonds in the presence of SiCl_4_ was unsuccessful, the use of pyridine as a chelating unit in combination with tetrachlorosilane gave promising results.

These species were tested as organocatalysts in the Lewis base-catalyzed Lewis acid-mediated addition of allyl tributyltin to benzaldehyde and other aromatic aldehydes, generating homoallylic alcohol with good yields and enantioselectivities. Further investigations to study the self-assembly recognition processes as well as the use of these chiral ligands in other silicon-based transformations are under investigation in our laboratories and will be reported in due course. 

## Figures and Tables

**Table 1:**
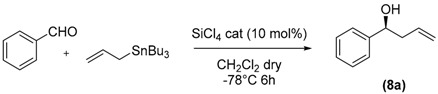
Enantioselective allylation of benzaldehyde.

Entry	Catalyst	Conc [M]	Yield (%)	ee (%) ^1^
1	**7i**	0.5	/	/
2	**7a**	0.5	83	13 (*S*)
3	**7b**	0.5	58	60 (*S*)
4	**7c**	0.5	73	70 (*S*)
5	**7c**	0.05	28	73 (*S*)
6	**7d**	0.5	78	68 (*S*)
7	**7e**	0.5	90	Rac
8	**7f**	0.5	45	44 (*S*)
9	**7g**	0.5	80	34 (*S*)
10	**7h**	0.5	79	60 (*S*)
11	**7l**	0.5	98	65 (*S*)

^1^ determined by HPLC in chiral stationary phase.

**Table 2:**
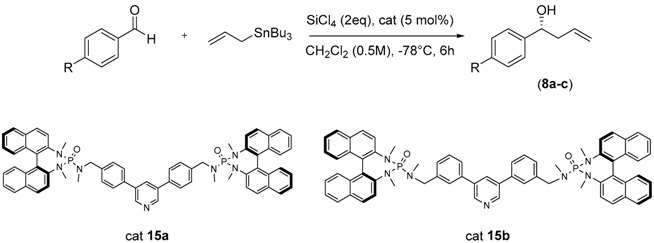
Enantioselective allylation of aldehydes.

Entry	Catalyst	R	Product	Yield (%)	ee (%) ^1^
1	**15a**	H	8a	71	61 (*S*)
2	**15b**	H	8a	30	29 (*S*)
3	**15a**	Cl	8b	83	62 (*S*)
4	**15a**	OMe	8c	31	rac

^1^ determined by HPLC on chiral stationary phase.

## Supplementary Materials

Supplementary Materials are available online.
