# Supplementary material for: Novel Chiral Bis-Phosphoramides as Organocatalysts for Tetrachlorosilane-Mediated Reactions
Source: Molecules. 2017 Dec 8;22(12):2181. doi: 10.3390/molecules22122181 (PMC6149666; doi:10.3390/molecules22122181)
Supplement: Supplementary file 1 [file molecules-22-02181-s001.pdf]

Novel chiral bis-phosphoramides as organocatalysts for tetrachlorosilane-mediated reactions

Sergio Rossi,* Marco Ziliani, Rita Annunziata and Maurizio Benaglia*

Department of Chemistry, Università degli Studi di Milano, via Golgi 19 20133 Milan (IT)

INDEX

1. General information pag *S-2*
2. General procedure A: synthesis of N-methyl-1-(nitrophenyl)methanamines pag *S-2*
3. General procedure B: synthesis of biphenylnitroamines pag *S-3*
4. General procedure C: synthesis of nitro-phosphoroamides pag *S-5*

   4.1 Synthesis of cyano-phosphoroamide 4e pag *S-8*
5. General procedure D: synthesis of amino-phosphoroamides pag *S-9*

   5.1 Synthesis of Phosphoroamide 5e pag *S-11*
6. General procedure E: synthesis of SAPAs pag *S-12*

   6.1. Synthesis of compound 7i pag *S-17*
7. General procedure F: synthesis of 3,5-bisubstituted pyridines pag *S-18*
8. General procedure G: synthesis of bis(*N*-methylamino)-pyridines pag *S-19*
9. General procedure H: synthesis of bisphosphoroamides 15a-b pag *S-20*
10. General procedure for allylation of aldehydes pag *S-23*
11. NMR Studies pag *S-26*
12. NMR Spectra pag *S-30*

1. General Information

^{1}H-NMR, ^{13}C-NMR and ^{29}Si-NMR spectra were recorded with instruments at 300 MHz (Bruker F300) or 500 MHz (Bruker ADVACE 500 or 600). Proton chemical shifts are reported in ppm (δ) with the solvent reference relative to tetramethylsilane (TMS) employed as the internal standard ($CDCl_3$ = δ 7.26 ppm). ^{13}C NMR spectra were recorded operating at 75 MHz, 125 MHz or 192.5 MHz, with complete proton decoupling. Carbon chemical shifts are reported in ppm (δ) relative to TMS with the respective solvent resonance as the internal standard ($CDCl_3$, δ = 77.0 ppm). ^{29}Si NMR spectra were recorded operating at 99 MHz, chemical shifts are reported in ppm (δ) relative to TMS. ^{31}P spectra were recorded at 121.4 or 202.4 MHz and were referenced to phosphoric acid (H_3PO_4) at 0.0 ppm. HPLC analysis was performed on an Agilent Instrument Series 1100 or 1200 series on chiral stationary phase. Purification of the products was performed by column chromatography on silica gel (230–400 mesh ASTM, Merck).

All the solvents were used are commercially available (≥ 99%, chromatographic grade, purchased from Sigma Aldrich) and stored under nitrogen over molecular sieves (bottles with crown cap). Reactions were monitored by analytical thin-layer chromatography (TLC) using silica gel 60 F_{254} pre-coated glass plates and visualized using UV light.

Compounds **1,**[1]**6a,**[2] and **6b**[2] are all known and were synthetized according to literature procedures. **3e**, **3l, 12a**, **12b** are commercially available and were used as received.

2. General procedure A: synthesis of N-methyl-1-(nitrophenyl)methanamines (3a), (3b), (3c)

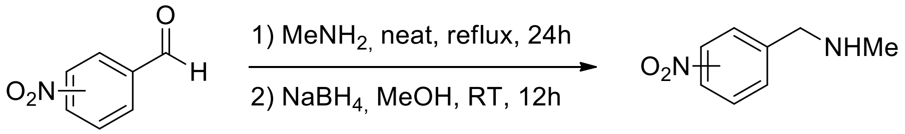

The desired nitro-substituted benzaldehyde (1 eq, 13.2 mmol, 2.0 g), methylamine (40% water solution, 1.1 eq, 14.5 mmol, 0.95 mL) and molecular sieves (3A, 100 mg) were introduced in a screw cap vial and the mixture was heated at 80°C for 24h. After cooling to RT, the mixture was diluted with chloroform (3 mL) and filtered. The solvent was removed by rotary evaporation and the crude obtained was redissolved in methanol (30 mL) and cooled to 0 °C. $NaBH_4$ (1.1 eq, 14.5 mmol, 550 mg) was added in three portions and the resultant mixture allowed warming to RT and reacting for 24 h. The mixture was then quenched with aq. NH_4OH 5% (10 mL) and the solvent was concentrated by rotary evaporation. The obtained aqueous layer was extracted with CH_2Cl_2 (3 x 10 mL). The combined organic extracts were dried over Na_2SO_4, filtered and the filtrate was concentrated by rotary evaporation. The residue was purified by silica gel flash chromatography or distillation under vacuum to give the title compound.

N-methyl-1-(2-nitrophenyl)methanamine (**3a**)

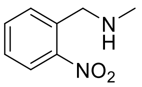

The crude mixture was purified by distillation under vacuum to yield the product as a yellow oil in 84% yield.
^{1}H NMR (300 MHz; $CDCl_3$): δ 7.92 (d, J = 8.2 Hz, 1H), 7.60-7.58 (m, 2H), 7.40 (t, J = 8.2 Hz, 1H), 3.98 (s, 2H), 2.44 (s, 3H), 1.74 (br, 1H).

N-methyl-1-(3-nitrophenyl)methanamine (**3b**)

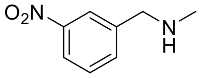

The crude mixture was purified by silica gel flash chromatography using CH_2Cl_2/MeOH (9:1, v:v) as eluent. The product was isolated as yellow oil in 86% yield.
^{1}H NMR (300 MHz; $CDCl_3$): δ 8.16 (s, 1H), 8.05 (d, J = 7.6 Hz, 1H), 7.65 (d, J = 7.6 Hz, 1H), 7.46 (t, J = 7.6 Hz, 1H) 3.84 (s, 2H), 2.45 (s, 3H), 2.04 (br, 1H).

N-methyl-1-(4-nitrophenyl)methanamine (**3c**)

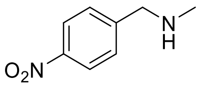

The crude product mixture purified by silica gel flash chromatography using Hex:AcOEt (1:1, v:v) as eluent. The product was isolated as yellow oil in 62% yield.
^{1}H NMR (300 MHz; $CDCl_3$): δ 8.18 (d, J = 8.7 Hz, 2H), 7.50 (d, J = 8.7 Hz, 2H), 3.86 (s, 2H), 2.46 (s, 3H), 1.56 (br, 1H).

3. General procedure B: synthesis of biphenylnitroamines (3f), (3g), (3h):

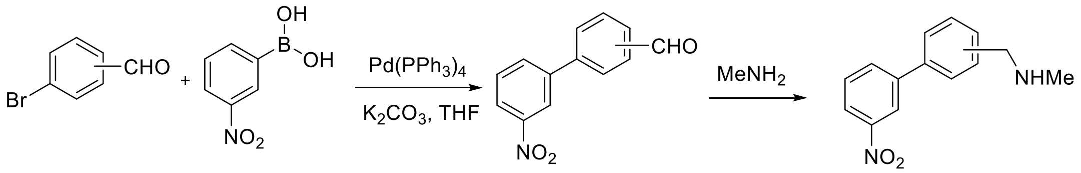

$Pd(OAc)_2$ (0.2 eq, 0.54 mmol, 122 mg) and PPh_3 (0.8 eq, 2.18 mmol, 572 mg) were suspended in dry THF (15 mL). After 15 min of stirring at RT, 3-nitrophenyl boronic acid (1.1 eq, 3 mmol, 500 mg), the desired bromo-benzaldehyde (2 eq, 5.45 mmol, 1 g) and potassium carbonate (3 eq, 8.13 mmol, 1,12 g) were added. The mixture was heated under gently reflux for 18 h. After that, the mixture was cooled to RT, diluted with diethyl ether (30 mL) and washed with brine. The obtained aqueous layer was washed with diethyl ether (2 x 10 mL). The combined organic extracts were dried over Na_2SO_4, filtered and was concentrated by rotary evaporation.

The residue was purified by silica gel flash chromatography using hexane/ethyl acetate (8:2, v:v) as eluent, leading the desired products. The aldehydes were then converted to the corresponding amines **3f**, **3g**, **3h** according to the general procedure A.

3'-nitro-[1,1'-biphenyl]-2-carbaldehyde

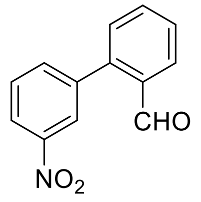

The crude product was purified by silica gel flash chromatography using hexane/ethyl acetate (8:2, v:v) as eluent. The product-containing fractions were combined and the solvent was removed by rotary evaporation to yield the product with 94% yield.

^{1}H NMR (300 MHz; $CDCl_3$): δ 9.97 (s, 1H), 8.37–8.22 (m, 2H), 8.06 (d, J = 7.7 Hz, 1H), 7.73-7.61 (m, 4H), 7.44 (d, J = 7.6 Hz, 1H).

Compound (**3f**)

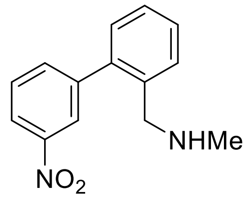

The residue was purified by silica gel flash chromatography using CH_2Cl_2/MeOH (9:1, v:v) as eluent. The product-containing fractions were combined and the solvent was removed by rotary evaporation under vacuum to yield the product in 31% yield.

^{1}H NMR (300 MHz,$CDCl_3$): δ 8.38 (s, 1H), 8.25 (d, J = 8.2 Hz, 1H), 7.80 (d, J = 7.7 Hz, 1H), 7.61 (t, J = 7.9 Hz, 1H), 7.54 (d, J = 7.4 Hz, 1H), 7.45 (dd, J = 7.3, 1.6 Hz, 1H), 7.41-7.37 (m, 2H), 7.29 (d, J = 7.0 Hz, 1H), 3.67 (s, 2H), 2.40 (s, 3H), 1.74 (br, 1H).

3'-nitro-[1,1'-biphenyl]-3-carbaldehyde

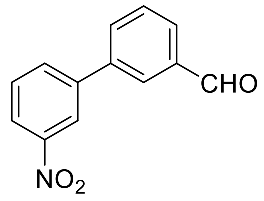

The crude product was purified by silica gel flash chromatography using hexane/ethyl acetate (8:2, v:v) as eluent. The product-containing fractions were combined and the solvent was removed by rotary evaporation to yield the product with 87% yield.

^{1}H NMR (300 MHz; $CDCl_3$): δ 10.13 (s, 1H), 8.50 (s, 1H), 8.27 (d, J = 8.3 Hz, 1H), 8.15 (s, 1H), 7.96 (t, J = 6.8 Hz, 1H), 7.91 (d, J = 7.8 Hz, 1H), 7.74-7.67 (m, 2H).

Compound (**3g**)

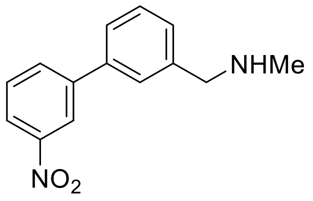

The residue was purified by silica gel flash chromatography using CH_2Cl_2/MeOH (9:1, v:v) as eluent. The product-containing fractions were combined and the solvent was removed by rotary evaporation under vacuum to yield the product in 66% yield.

^{1}H NMR (300 MHz,$CDCl_3$): δ 8.48 (s, 1H), 8.21 (d, J = 8.1 Hz, 1H), 7.95 (d, J = 7.7 Hz, 1H), 7.72-7.38 (m, 5H), 3.88 (s, 2H), 2.53 (s, 3H), 2.26 (br, 1H).

3'-nitro-[1,1'-biphenyl]-4-carbaldehyde

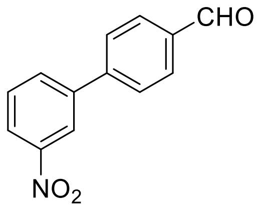

The crude product was purified by silica gel flash chromatography using hexane/ethyl acetate (8:2, v:v) as eluent. The product-containing fractions were combined and the solvent was removed by rotary evaporation to yield the product with 85% yield.

^{1}H NMR (300 MHz; $CDCl_3$): δ 10.13 (s, 1H), 8.53 (s, 1H), 8.31 (d, J = 8.2 Hz, 1H), 8.05 (d, J = 8.2 Hz, 2H), 7.99 (d, J = 7.8 Hz, 1H), 7.83 (d, J = 8.2 Hz, 2H), 7.70 (t, J = 8.0 Hz, 1H)

Compound (**3h**)

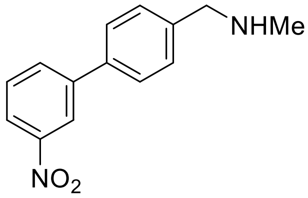

The residue was purified by silica gel flash chromatography using CH_2Cl_2/MeOH (9:1, v:v) as eluent. The product-containing fractions were combined and the solvent was removed by rotary evaporation under vacuum to yield the product in 60% yield.

^{1}H NMR (300 MHz,$CDCl_3$): δ 8.46 (s, 1H), 8.20 (d, J = 7.7 Hz, 1H), 7.93 (d, J = 7.7 Hz, 1H), 7.62 (d, J = 6.5 Hz, 2H), 7.49 (d, J = 6.5 Hz, 2H), 3.86 (s, 2H), 2.52 (s, 3H).

4. General procedure C: synthesis of nitro-phosphoroamides (4a), (4b), (4c), (4d), (4f), (4g), (4h) and (4l):

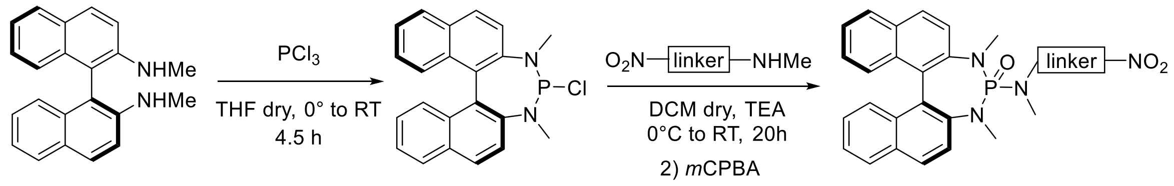

N,N’-dimethyl-1,1’-binaphthyl-2,2’-diamine (**1**) (1 eq, 3.20 mmol, 1.0 g) and Et_3N (3 eq, 9.6 mmol, 1.33 mL) were dissolved in dry THF (32 mL). The homogeneous mixture was cooled to 0 °C then PCl_3 (3 eq, 9.60 mmol, 0.84 mL) was added dropwise via syringe whereupon a colorless precipitate formed immediately. The reaction mixture was stirred at 0 °C for 1.5 h, then was allowed to warm to room temperature and stirred for another 3 h. The volatiles were removed under high vacuum (room temperature, 0.5mmHg) and Et_2O (30.0 mL) was added via syringe, then the mixture was stirred for 5 min. After that the supernatant was canula-filtered into another round bottom flask. The remaining precipitate in the reaction flask was washed again with Et_2O (30 mL) and filtered (2 times). The volatiles were removed under high vacuum (room temperature, 0.5 mmHg) to afford a light yellow solid. The solid was then dried for 12 h at reduced pressure (room temperature, 0.5 mmHg) to give a white solid foam (**2**). Dry CH_2Cl_2 (40 mL) was added via syringe and the mixture was cooled to 0 °C. To this solution, a mixture of Et_3N (2 eq, 6.40 mmol, 0.98 mL) and the desired methylamine (**3**) (1.2 eq, 3.84 mmol) dissolved in dry CH_2Cl_2 (4 mL) were added. The reaction mixture was

allowed to warm to room temperature and stirred for 20 h. A solution of *m*CPBA (70%) (1.5 eq, 4.80 mmol, 1.18 g) dissolved in 2 mL of THF was then added and the mixture was stirred for 20 h. After a quench with 15 mL of NH_4Cl saturated aqueous solution, the phases were separated and aqueous layer was washed with CH_2Cl2 (10 mL). The combined organic extracts were washed with brine, dried over Na_2SO_4, filtered and concentrated by rotary evaporation. The crude residue was purified by silica gel flash chromatography using ethyl acetate (100%) as eluent to yield phosphoroamides with different yields.

Phosphoroamide **4a**

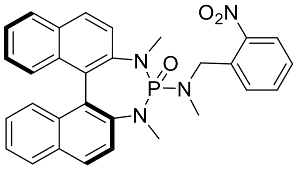

The product was obtained starting from *N*-methyl-1-(2-nitrophenyl)methanamine **3a** in 62% yield.

^{1}H NMR (300 MHz; $CDCl_3$): δ 8.00-7.82 (m, 8H), 7.51-7.07 (m, 8H), 4.63 63 (dd, J = 17.1, 8.1 Hz, 1H), 4.41 (dd, J = 17.2, 9.7 Hz, 1H), 3.11 (d, J = 10.3 Hz, 3H), 3.04 (d, J = 8.8 Hz, 3H), 2.33 (d, J = 8.9 Hz ,3H).

^{31}P NMR (121.4 MHz; $CDCl_3$): δ 29.21.

MS (ESI-): calculated for $C_{30}H_{27}N_4O_3P$ = 522.18 Found *m/z* = 545.71 $[M + Na]^-$.

Phosphoroamide **4b**

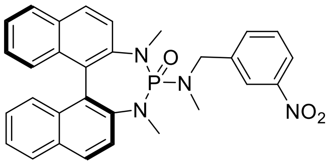

The product was obtained starting from *N*-methyl-1-(3-nitrophenyl)methanamine **3b** in 76% yield.

^{1}H NMR (300 MHz; $CDCl_3$): δ 8.34 (s, 1H), 8.20 (d, J = 8.1 Hz, 1H), 8.02 (d, J = 8.9 Hz, 1H), 7.89-7.85 (m, 3H), 7.78 (d, J = 8.0 Hz, 2H), 7.58-7.50 (m, 2H), 7.44-7.35 (m, 2H), 7.26-7.22 (m, 2H), 7.19-7.16 (m, 1H), 7.09 (d, J = 8.9 Hz, 1H), 4.62 (dd, J = 15.3, 7.5 Hz, 1H), 4.23 (dd, J = 15.3, 10.7 Hz, 1H), 3.13 (d, J = 2.5 Hz, 3H), 3.09 (d, J = 3.9 Hz, 3H), 2.18 (d, J = 8.7 Hz, 3H).

^{13}C NMR (75 MHz, $CDCl_3$) δ 148.5, 142.6, 141.3, 134.4, 132.6, 132.6, 132.5, 131.3, 129.8, 129.5, 129.4, 129.0, 128.1, 127.9, 127.4, 127.1, 126.3, 126.2, 125.4, 125.1, 123.4, 122.6, 122.4, 53.2, 36.2, 35.1, 34.5.

^{31}P NMR (121.4 MHz; $CDCl_3$): δ 28.76.

MS (ESI-): calculated for $C_{30}H_{27}N_4O_3P$ = 522.53 Found *m/z* = 521.56 $[M - 1]^-$.

Phosphoroamide **4c**

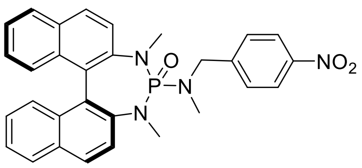

The product was obtained starting from *N*-methyl-1-(4-nitrophenyl)methanamine **3c** in 93% yield.

^{1}H NMR (300 MHz; $CDCl_3$): δ 8.24 (d, J = 8.4 Hz, 2H), 7.99 (d, J = 8.8 Hz, 1H), 7.87 (d, J = 8.6 Hz, 2H), 7.74 (d, J = 8.8 Hz, 2H), 7.58 (d, J = 8.4 Hz, 2H), 7.43-7.33 (m, 3H), 7.25-7.20 (m, 2H) 7.15 (t, J = 7.6 Hz, 1H) 7.05 (d, J = 8.6 Hz, 1H), 4.43 (dd, J = 15.3, 7.5 Hz, 1H), 4.26 (dd, J = 15.1, 10.3 Hz, 1H), 3.07 (d, J = 3.0 Hz, 3H), 3.04 (s, 3H), 2.17 (d, J = 8.7 Hz, 3H).

^{13}C NMR (75 MHz, $CDCl_3$) δ 146.9, 146.0, 142.1, 140.8, 132.2, 132.1, 130.9, 130.8, 129.4, 128.8, 128.8, 127.6, 127.5, 127.4, 126.9, 126.6, 125.9, 125.7, 125.0, 124.6, 123.3, 122.8, 122.1, 52.8, 35.7, 34.5, 34.2.

^{31}P NMR (121.4 MHz; $CDCl_3$): 28.82

MS (ESI-): calculated for $C_{30}H_{27}N_4O_3P$ = 522.18 Found *m/z* = 521.45 [M - 1]$^-$.

Phosphoroamide **4f**

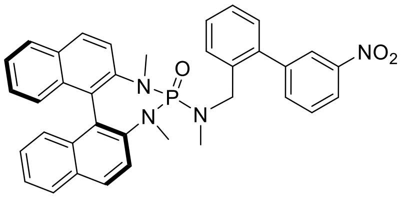

The product was obtained starting from amine **3e** in 87% yield.

^{1}H NMR (300 MHz; $CDCl_3$): δ 8.16 (d, J = 6.7 Hz, 1H), 8.04 (d, J = 7.2 Hz, 1H), 7.95-7.92 (m, 3H), 7.85-7.78 (m, 3H), 7.72 (d, 1H), 7.55-7.30 (m, 6H), 7.20 (m, 3H), 7.12 (t, J = 6.8 Hz, 1H), 7.02 (d, , J = 8.6 Hz, 1H), 4.24 (dd, J = 15.2, 7.2 Hz, 1H), 4.03 (dd, J = 15.9, 9.2 Hz, 1H), 3.04 (t, J = 9.2 Hz), 2.18 (d, J = 9.0 Hz, 3H).

^{31}P NMR (121.4 MHz; $CDCl_3$): 29.34.

MS (ESI-Q-TOF): calculated for $C_{36}H_{31}N_4O_3P$: 598.21 Found *m/z* = 599.16 [M + 1]$^+$ and 621.13 [M + Na]$^+$

Phosphoroamide **4g**

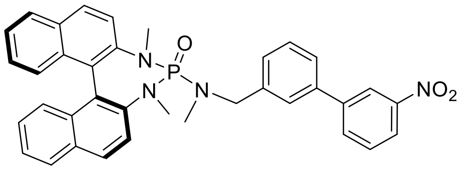

The product was obtained starting from amine **3g** in 62% yield.

^{1}H NMR (300 MHz; $CDCl_3$): δ 8.34 (s, 1H), 8.06 (d, J = 8.2 Hz 1H), 7.85-7.71 (m, 5H), 7.73 (d, J = 8.6 Hz, 1H), 7.52-7.18 (m, 8H), 7.09 (m, 2H), 7.01 (t, 1H), 6.93 (d, J = 8.2 Hz, 1H), 4.30 (dd, J = 15.1, 7.0 Hz, 1H), 4.15 (J = 14.6, 10.7 Hz, 1H), 2.96 (dd, J = 9.6, 5.1 Hz, 6H), 2.10 (d, J = 8.8 Hz, 3H).

^{31}P NMR (121.4 MHz; $CDCl_3$): 29.16.

Phosphoroamide **4h**

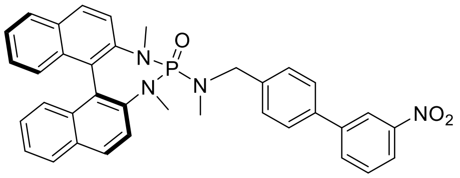

The product was obtained starting from amine **3h** in 45% yield.

^{1}H NMR (300 MHz; $CDCl_3$): δ 8.50 (s, 1H), 8.23 (d, J = 8.1 Hz, 1H), 8.06 (s, 1H), 7.98-7.86 (m, 5H), 7.80 (d, J = 8.8 Hz, 1H), 7.66-7.62 (m, 2H), 7.55-7.32 (m, 5H), 7.28-7.24 (m, 2H), 7.17 (t, 1H), 7.10 (d, J = 8.1 Hz, 1H), 4.45 (dd, J = 14.9, 7.3 Hz, 1H), 4.27 (dd, J = 14.9, 10.4 Hz, 1H), 3.12 (d, J = 10.3 Hz, 6H), 2.24 (d, J = 8.9 Hz, 3H).

^{31}P NMR (121.4 MHz; $CDCl_3$): 29.09.

Phosphoroamide **7l**

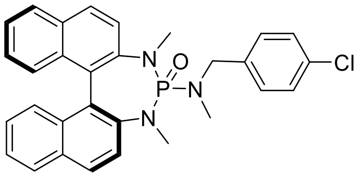

The product was obtained starting from amine **3l** in 31% yield.

^{1}H NMR (300 MHz; $CDCl_3$): δ 7.97 (d, J = 8.9 Hz, 2H), 7.86 (d, J = 8.8 Hz, 2H), 7.75 (d, J = 8.9 Hz, 2H), 7.42-7.34 (m, 5H),, 7.26-7.20 (m, 3H), 7.13 (d, J = 7.2 Hz, 1H), 7.06 (d, J = 8.5 Hz, 1H), 4.31 (dd, J = 15.1, 7.6 Hz, 1H), 4.13 (dd, J = 14.9, 10.3 Hz, 1H), 3.06 (d, J = 1.7 Hz, 3H), 3.03 (d, J = 3.1 Hz, 3H), 2.16 (d, J = 9.0 Hz, 3H).

^{31}P NMR (121.4 MHz; $CDCl_3$): 29.06.

MS (ESI-Q-TOF): calculated for $C_{30}H_{27}ClN_3OP$ = 511.16 Found *m/z* = 512.14 $[M + 1]^+$ and 534.11 $[M + Na]^+$

*4.1 Synthesis of cyano-phosphoroamide **4e***

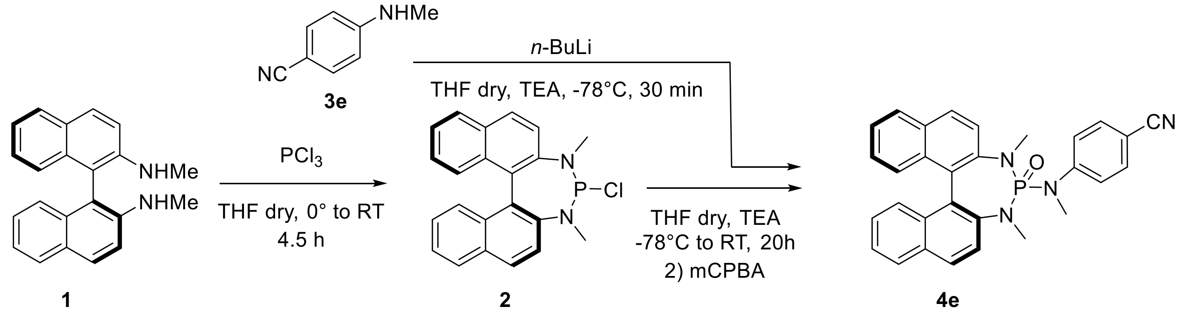

N,N'-dimethyl-1,1'-binaphthyl-2,2'-diamine (**1**) (1 eq, 0.96 mmol, 300 mg) and Et_3N (2.5 eq, 2.4 mmol, 0.34 mL) were dissolved in dry THF (21 mL). The homogeneous mixture was cooled to 0 °C then PCl_3 (3 eq, 9.60

mmol, 0.84 mL) was added dropwise via syringe whereupon a colorless precipitate formed immediately. The reaction mixture was stirred at 0 °C for 1.5 h, then was allowed to warm to room temperature and stirred for another 3 h. The volatiles were removed under high vacuum (room temperature, 0.5mmHg) and Et_2O (30.0 mL) was added via syringe, then the mixture was stirred for 5 min. After that the supernatant was canula-filtered into another round bottom flask. The remaining precipitate in the reaction flask was washed again with Et_2O (5 mL) and filtered (3 times). The volatiles were removed under high vacuum (room temperature, 0.5 mmHg) to afford a light yellow solid. The solid was then dried for 12 h at reduced pressure (room temperature, 0.5 mmHg) to give a white solid foam (**2**). Dry CH_2Cl_2 (40 mL) was added via syringe and the mixture was cooled to -78 °C with a dry ice-acetone bath. In a second round bottom flask, 4-(methylamino)benzonitrile **3e** (1.1 eq, 1.06 mmol, 139.6 mg) was dissolved in 10 mL of THF and cooled to -78 °C, then nBuLi 1.6 M (1.1 eq, 1.06 mmol, 0.66 mL), was slowly added. This solution was transfer in the round bottom flask containing (**3**) and the reaction mixture was allowed to warm to room temperature and then stirred for 20 h. *m*CPBA (70%) (1.5 eq, 1.44 mmol, 354 mg) dissolved in 2 mL of THF was then added and the mixture was stirred for 20 h. After a quench with 5 mL of NH_4Cl saturated aqueous solution, the phases were separated and aqueous layer was washed with CH_2Cl_2 (10 mL). The combined organic extracts were washed with brine, dried over Na_2SO_4, filtered and concentrated by rotary evaporation. The crude residue was purified by silica gel flash chromatography using ethyl acetate/hexane (9:1 v:v) as eluent.

The title compound was obtained in 48% yield.

^{1}H NMR (300 MHz; $CDCl_3$): δ 8.04 (d, J = 8.9 Hz, 1H), 7.92 (dd, J = 8.2, 3.2 Hz, 1H), 7.88 (d, J = 9.0 Hz, 1H), 7.78 (d, J = 9.7 Hz, 1H), 7.50-7.34 (m, 8H), 7.28-7.26 (m, 2H), 7.18 (dd, J = 8.9, 1.5 Hz, 1H), 7.07 (d, J = 8.5 Hz, 1H), 3.18 (d, J = 10.9 Hz, 3H), 3.09 (d, J = 9.2 Hz, 3H), 2.90 (d, J = 7.9 Hz, 3H).

^{31}P NMR (121.4 MHz; $CDCl_3$): 24.02.

MS (ESI-Q-TOF): calculated for $C_{30}H_{25}N_4OP$ = 488.18 Found *m/z* = 489.13 $[M + 1]^+$ and 511.11 $[M + Na]^+$

5. General procedure D: synthesis of amino-phosphoroamides (5a), (5b), (5c), (5d), (5f), (5g), (5h):

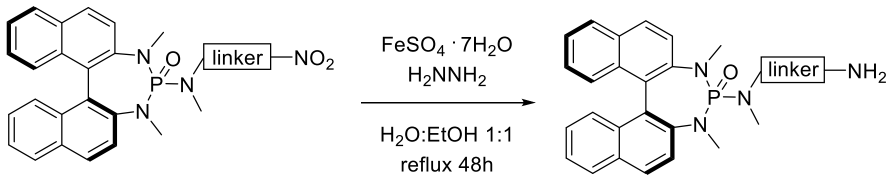

(**4**) (**5**)

Phosphoroamide **4** (1 eq, 1 mmol), $FeSO_4 \cdot 7H_2O$ (0.5 eq, 0.5 mmol, 139 mg) and hydrazine mono hydrate (10 eq, 10 mmol, 0.491 uL) were dissolved in a water:ethanol 1:1 solution (5ml:5mL) and stirred for 10 min, then the heterogeneous mixture was gently refluxed for 48 h. After this time the mixture was cooled to RT, diluted with AcOEt (15 mL), and the phases were separated. The organic extracts were dried over Na_2SO_4, filtered

and concentrated by rotary evaporation under vacuum. The residue was purified by silica gel flash chromatography using ethyl acetate (100%) as eluent to yield phosphoroamides with different yields.

Phosphoroamide **5a**

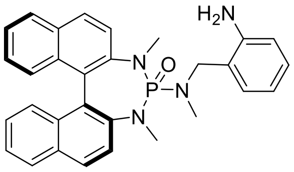

The product was obtained starting from phopsphoroamide **4a** in 92% yield.

^{1}H NMR (300 MHz; $CDCl_3$): δ 7.98-7.85 (m, 4H), 7.73 (d, *J* = 8.8 Hz, 1H), 7.56 (d, *J* = 8.8 Hz, 1H), 7.43-7.33 (m, 2H), 7.27-7.21 (m, 2H), 7.17-7.04 (m, 3H), 6.97 (d, *J* = 7.4 Hz, 1H), 6.66 (d, *J* = 7.9 Hz, 1H), 6.60 (t, *J* = 7.3 Hz, 1H), 4.61 (br, 2H), 4.41 (dd, *J* = 14.4, 9.2 Hz, 1H), 4.17-4.09 (m, 1H), 3.11 (d, J = 9.6, 3H), 3.04 (d, J = 8.1, 3H), 2.07 (d, *J* = 9.1 Hz, 3H).

^{31}P NMR (121.4 MHz; $CDCl_3$): 30.13.

Phosphoroamide **5b**

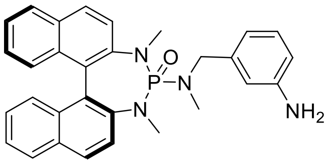

The product was obtained starting from phopsphoroamide **4b** in 98% yield.

^{1}H NMR (300 MHz; $CDCl_3$): δ δ 7.97 (d, J = 8.9 Hz, 1H), 7.85 (m, 3H), 7.74 (d, J = 8.8 Hz, 1H), 7.42 (d, J = 8.8 Hz, 1H), 7.35 (m, 2H), 7.22 (m, 2H), 7.13 (t, 2H), 7.06 (d, 1H), 6.73 (m, 2H), 6.60 (d, J = 7.8 Hz, 1H), 4.20 (dd, J = 14.7, 7.3 Hz, 1H), 4.02 (dd, J = 14.8, 10.4 Hz, 1H), 3.65 (br, 1H) 3.07 (d, J = 7.1 Hz, 3H), 3.04 (d, J = 9.2 Hz, 3H), 2.20 (d, J = 9.0 Hz, 3H).

^{31}P NMR (121.4 MHz; $CDCl_3$): δ 29.27.

Phosphoroamide **5c**

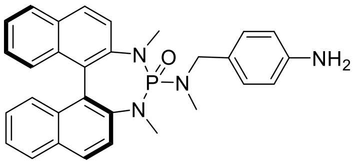

The product was obtained starting from phopsphoroamide **4c** in 92% yield.

^{1}H NMR (300 MHz; $CDCl_3$): δ 7.98 (d, J = 8.9 Hz, 1H), 7.90-7.84 (m, 3H), 7.76 (d, J = 8.8 Hz, 1H), 7.45-7.36 (m, 3H), 7.28-7.14 (m, 5H), 7.05 (d, J = 8.5 Hz, 1H), 6.74 (d, J = 7.9 Hz, 2H), 4.20 (dd, J = 14.5, 7.2 Hz, 1H), 4.03 (dd, J = 14.3, 10.4 Hz, 1H), 3.04 (d, J = 4.8 Hz, 3H), 3.01 (d, J = 6.2 Hz, 3H), 2.13 (d, J = 9.0 Hz, 3H).

^{31}P NMR (121.4 MHz; $CDCl_3$): δ 29.30.

Phosphoroamide **5f**

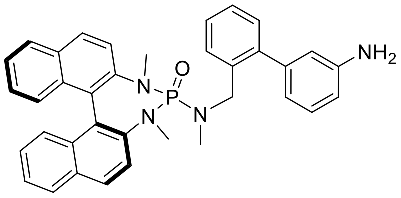

The product was obtained starting from phopsphoroamide **4f** in 17% yield.

^{1}H NMR (300 MHz; $CDCl_3$): δ 8.16 (d, J = 7.5 Hz, 1H), 8.05 (s, 1H) 7.96 (d, J = 9.0 Hz, 1H), 7.85-7.71 (m, 5H), 7.49-7.01 (m, 11H), 4.25-4.10 (m, 1H), 4.09-3.85 (m, 1H) 3.10 (d, 3H), 3.04 (d, J = 8.9 Hz, 6H), 2.16 (d, J = 8.8 Hz, 3H), 1.25 (br, 2H).

^{31}P NMR (121.4 MHz; $CDCl_3$): 29.34.

Phosphoroamide **5g**

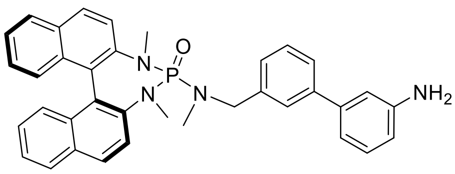

The product was obtained starting from phopsphoroamide **4g** in 85% yield.

^{1}H NMR (300 MHz; $CDCl_3$): δ 7.97 (d, J = 9.1 Hz, 1H), 7.85-7.71 (m, 6H), 7.49-7.01 (m, 12H), 6.67 (d, J = 8.1 Hz, 1H), 4.39 (dd, J = 15.8, 7.0 Hz, 1H), 4.12 (dd, J = 17.1, 8.0, 1H), 3.75 (br, 2H), 3.06 (d, J = 7.1 Hz, 6H), 2.23 (d, J = 9.0 Hz, 3H).

^{31}P NMR (121.4 MHz; $CDCl_3$): 29.82.

Phosphoroamide **5h**

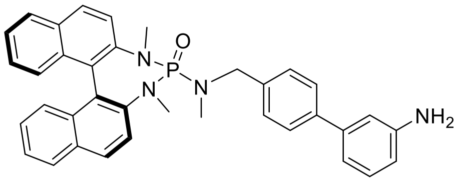

The product was obtained starting from phopsphoroamide **4h** in 37% yield.

^{1}H NMR (300 MHz; $CDCl_3$): δ 7.98 (d, J = 9.1 Hz, 1H), 7.90-7.79 (m, 3H), 7.76 (d, J = 8.8 Hz, 1H), 7.66-7.51 (m, 2H), 7.44-7.34 (m, 5H), 7.23 (t, J = 7.9 Hz, 3H), 7.14 (t, J = 7.9 Hz, 1H), 7.07-6.98 (m, 2H), 6.94 (s, 1H), 6.68 (d, J = 7.9 Hz, 1H), 4.36 (dd, J = 14.7, 7.3 Hz, 1H), 4.15 (dd, J = 16.7, 9.2 Hz, 2H), 3.07 (d, J = 10.0 Hz, 6H), 2.21 (d, J = 9.0 Hz, 3H).

^{31}P NMR (121.4 MHz; $CDCl_3$): 29.02.

Synthesis of Phosphoroamide **5e**

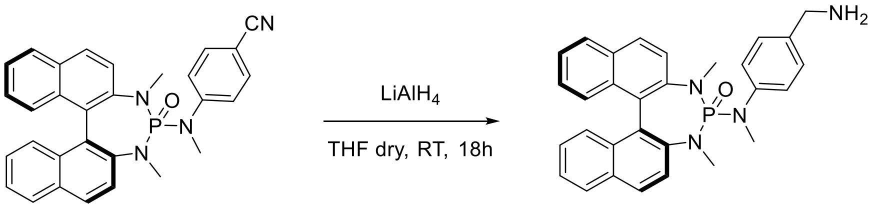

4e **5e**

A solution of phosphoroamide **4e** (1 eq, 1 mmol, 488.5 mg) in dry THF (4 mL) was slowly added to a 0 °C suspension of $LiAlH_4$ (1.5 eq, 1.5 mmol, 56.9 mg) in THF (1 mL). The mixture was allow to reach RT and was stirred for 18 h. After this time, a quench with $NaHCO_3$ ss (1.0 mL) was performed. The resultant gray precipitate was filtered through Celite pad and washed with diethyl ether (3 x 10 mL). The organic solutions were combined and the solvent was removed by rotary evaporation under vacuum. The crude compound was purified by silica gel flash chromatography using CH_2Cl_2/MeOH (9:1, v:v) as eluent allowing to obtain the title compound in 76% yield.

^{1}H NMR (300 MHz; $CDCl_3$): δ δ 7.99 (d, J = 8.9 Hz, 1H), 7.86 (dd, J = 8.2, 4.0 Hz, 2H), 7.79 (dd, J = 12.7, 8.8 Hz, 2H), 7.39-7.27 (m, 3H), 7.25-7.10 (m, 6H), 3.81 (s, 2H),3.21 (d, J = 10.6 Hz, 3H), 3.02 (d, J = 9.1 Hz, 3H), 2.77 (d, J = 8.1 Hz, 3H), 2.54 (br, 2H).

^{31}P NMR (121.4 MHz; $CDCl_3$): 25.27.

6. General procedure E: synthesis of SAPAs 7a-h:

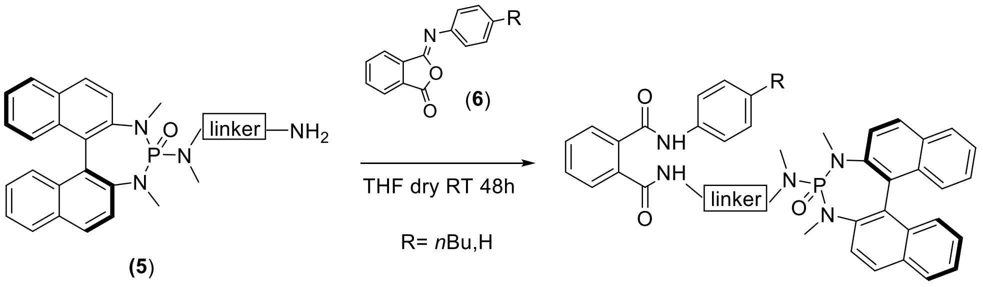

(**5**) (**7**)

The desired phosphoroamide (**5**) (1 eq, 0.1 mmol) and the desired phthalisoimide (**6**) (3 eq, 0.3 mmol) were dissolved in dry THF (2 mL). The homogeneous mixture was stirred at RT for 48 h, then quenched with 5.0 mL of HCl 5%. The phases, diluted with ethyl acetate were separated and the obtained aqueous layer was washed with ethyl acetate (5.0 mL). The combined organic extracts were washed with brine, dried over Na_2SO_4, filtered and the filtrate was concentrated by rotary evaporation. The residue was purified by silica gel flash chromatography using different mixtures furnishing the desired product in different yields.

Phosphoroamide **7a**

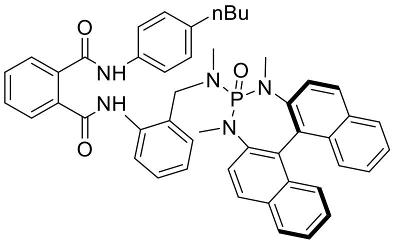

Phosphoroamide (**7a**) was synthetized starting from phosphoroamide (**5a**) and phthalisoimide (**6b**). The crude product was purified by silica gel flash chromatography using ethyl acetate (100%) as eluent. The product-containing fractions were combined and the solvent was removed by rotary evaporation under vacuum to yield the product with 44% yield.

^{1}H NMR (600 MHz; $CDCl_3$): δ 10.35 (s, 1H), 9.42 (s, 1H), 8.42 (d, *J* = 8.2 Hz, 1H), 7.94-7.85 (m, 4H), 7.78 (d, 1H), 7.76 (d, *J* = 7.0 Hz, 1H), 7.56-7.47 (m, 4H), 7.44-7.35 (m, 4H), 7.22-7.15 (m, 2H), 7.17-7.07 (m, 4H), 7.03-6.95 (m, 3H), 4.32 (dd, *J* = 14.3, 11.9 Hz, 1H), 4.16 (dd, *J* = 14.8, 6.4 Hz, 1H), 2.82 (d, *J* = 10.3 Hz, 3H), 2.70 (d, *J* = 9.3 Hz, 3H), 2.52 (t, *J* = 7.6 Hz, 2H), 2.09 (d, *J* = 9.1 Hz, 3H), 1.57-1.53 (m, 2H), 1.31-1.26 (m, 2H), 0.91 (t, *J* = 7.3 Hz, 3H).

^{13}C NMR (600 MHz; $CDCl_3$): δ 168.7, 166.8, 142.3, 140.8, 138.6, 137.0, 136.3, 136.2, 135.7, 132.5, 132.4, 131.2, 131.1, 130.8, 130.2, 130.0, 129.7, 129.3, 129.0, 128.8 (3C), 128.4, 128.1, 128.0, 127.8, 127.4, 127.3, 126.4, 126.2, 125.4, 125.2, 124.1, 124.0, 123.9, 122.9, 122.7, 119.9, 50.8 (2C), 35.5, 35.4, 35.0, 34.9, 34.5, 33.6, 22.2, 13.9.

^{31}P NMR (121.4 MHz; $CDCl_3$): δ 29.91.

MS (ESI-Q-TOF): calculated for $C_{48}H_{46}N_5O_3P$: 771.33 Found *m/z* = 794.33 [M + Na]$^+$

Phosphoroamide **7b**

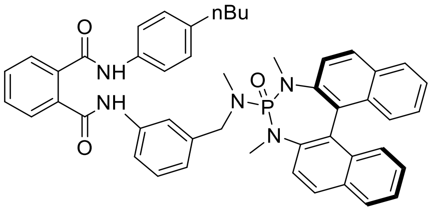

Phosphoroamide (**7b**) was synthetized starting from phosphoroamide (**5b**) and phthalisoimide (**6b**). The crude product was purified by silica gel flash chromatography using CH_2Cl_2/MeOH (98:2 v:v) as eluent. The product-containing fractions were combined and the solvent was removed by rotary evaporation under vacuum to yield the product with 51% yield

^{1}H NMR (300 MHz; $CDCl_3$): 9.82 (s, 1H), 9.60 (s, 1H), 7.92 (d, *J* = 8.8 Hz, 1H), 7.85 (d, *J* = 8.1 Hz, 1H), 7.79 (t, *J* = 7.6 Hz, 2H), 7.69-7.66 (m, 3H), 7.59-7.54 (m, 2H), 7.48-7.30 (m, 5H), 7.29-7.25 (m, 2H), 7.18-7.12 (m, 4H), 7.50 (s, 1H), 7.04 (d, *J* = 8.1 Hz, 1H), 6.97 (d, *J* = 8.1 Hz, 2H), 4.19 (dd, *J* = 14.4, 6.6 Hz, 1H), 3.98 (dd, *J* = 14.5,

10.3 Hz, 1H), 3.00 (d, *J* = 2.8 Hz, 3H), 2.97 (d, *J* = 4.6 Hz, 3H), 2.47 (t, *J* = 7.6 Hz, 2H), 2.11 (d, *J* = 8.8 Hz, 3H), 1.53-1.43 (m, 2H), 1.31-1.26j (m, 2H), 0.87 (t, *J* = 7.2 Hz, 3H).

^{13}C NMR (500 MHz; $CDCl_3$): δ 167.6, 167.4, 143.0, 141.5, 139.2 (2C), 138.5, 135.7, 135.1, 134.9, 132.6, 132.5, 131.3, 131.2, 130.4 (2C), 129.7, 129.3, 128.9 (2C), 128.7 (3C), 128.4, 128.1, 128.0, 127.8, 127.4, 127.3, 126.1, 126.0, 125.2, 125.0, 124.5, 123.3, 122.7, 120.3 (2C), 120.0, 119.4, 53.4, 36.0, 35.0 (2C), 34.2, 33.6, 22.3, 13.9,

^{31}P NMR (121.4 MHz; $CDCl_3$): δ 29.23.

MS (ESI+): calculated for $C_{48}H_{46}N_5O_3P$ = 771.33 Found *m/z* = 772.49 $[M]^+$ and 794.86 $[M + Na]^+$.

Phosphoroamide **7c**

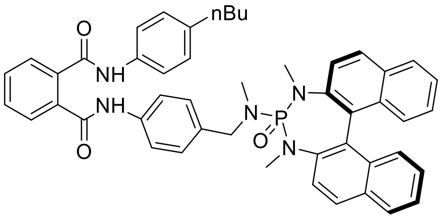

Phosphoroamide (**7c**) was synthetized starting from phosphoroamide (**5b**) and phthalisoimide (**6a**). The crude product was purified by silica gel flash chromatography using ethyl acetate/methanol (9:1 v:v) as eluent. The product-containing fractions were combined and the solvent was removed by rotary evaporation under vacuum to yield the product with 66% yield.

^{1}H NMR (300 MHz; $CDCl_3$): δ 9.25 (s, 1H), 9.15 (s, 1H), 7.93 (d, *J* = 8.9 Hz, 1H), 7.86-7.81 (m, 3H), , 7.67-7.63 (m, 6H), 7.51 (d, *J* = 8.1 Hz, 2H), 7.41-7.36 (m, 2H), 7.32-7.25 (m, 2H), 7.25-7.20 (m, 4H), 7.17-7.10 (m, 1H), 7.05 (d, *J* = 8.4 Hz, 3H), 4.16 (dd, *J* = 15.2, 7.4 Hz, 1H), 4.04 (dd, *J* = 14.5, 10.1 Hz, 1H), 2.97 (d, *J* = 3.6 Hz, 3H), 2.94 (d, *J* = 2.2 Hz, 3H), 2.52 (t, *J* = 7.7 Hz, 2H), 2.12 (d, *J* = 8.9 Hz, 3H), 1.58-1.48 (m, 2H), 1.32-1.25 (m, 2H), 0.89 (t, *J* = 7.3 Hz, 3H)

MS (ESI-Q-TOF): calculated for $C_{48}H_{46}N_5O_3P$: 771.33 Found *m/z* = 794.29 $[M + Na]^+$

Phosphoroamide **7d**

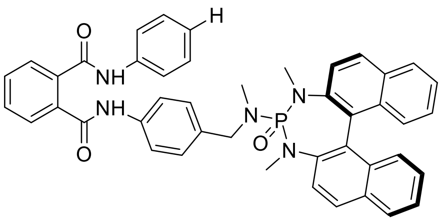

Phosphoroamide (**7d**) was synthetized starting from phosphoroamide (**5b**) and phthalisoimide (**6b**). The crude product was purified by silica gel flash chromatography using ethyl acetate (100%) as eluent. The product-containing fractions were combined and the solvent was removed by rotary evaporation under vacuum to yield the product with 66% yield.

^{1}H NMR (300 MHz; $CDCl_3$): δ 9.25 (s, 1H), δ 9.15 (s, 1H), δ 7.94-7.91 (m, 1H), δ 7.86-7.81 (m, 3H), δ 7.53 (d, 1H), δ 7.67-7.33 (m, 6H), δ 7.51 (d, 2H), δ 7.38 (t, 2H), δ 7.30 (t, 2H), δ 7.25-7.20 (m, 4H), δ 7.31 (t, 1H), δ 7.04 (d 3H), δ 4.25 (dd, *J* = 14.4, 6.8 Hz, 1H), δ 4.15 (dd, J = 14.1, 10.1 Hz, 1H), δ 2.97 (d, 6H), δ 2.12 (d, *J* = 7.2 Hz, 3H).

^{31}P NMR (121.4 MHz; $CDCl_3$): δ 29.17.

Phosphoroamide **7e**

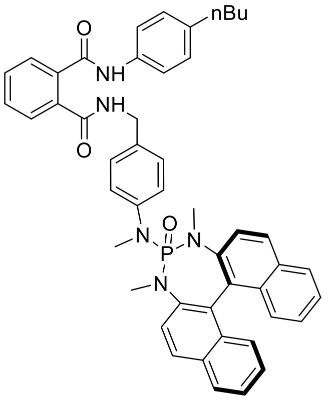

Phosphoroamide (**7e**) was synthetized starting from phosphoroamide (**5e**) and phthalisoimide (**6b**). The crude product was purified by silica gel flash chromatography using CH2Cl2/MeOH (95:5 v:v) as eluent. The product-containing fractions were combined and the solvent was removed by rotary evaporation under vacuum to yield the product with 57% yield.

^{1}H NMR (300 MHz; $CDCl_3$): δ 9.35 (s, 1H), 8.00 (d, J = 8.8 Hz, 1H), 7.90 (d, J = 8.2 Hz, 1H), 7.80 (d, J = 9.0 Hz, 2H), 7.73 (d, J = 8.8 Hz, 1H), 7.52 (d, J = 7.2 Hz, 2H), 7.45-7.35 (m, 6H), 7.29-7.23 (m, 3H), 7.17-7.02 (m, 7H), 4.46 (d, J = 5.7 Hz, 2H), 3.17 (d, J = 10.6 Hz, 3H), 2.98 (d, J = 9.1 Hz, 3H), 2.77 (d, J = 8.0 Hz, 3H), 2.58 (t, J = 7.7 Hz, 2H), 1.58 (m, 2H), 1.36 (d, J = 7.6 Hz, 2H), 0.93 (t, J = 7.3 Hz, 3H).

^{31}P NMR (121.4 MHz; $CDCl_3$): δ 29.23.

Phosphoroamide **7f**

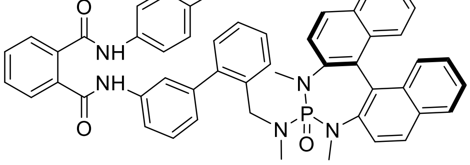

Phosphoroamide (**7f**) was synthetized starting from phosphoroamide (**5f**) and phthalisoimide (**6b**). The crude product was purified by silica gel flash chromatography using CH2Cl2/MeOH (98:2 v:v) as eluent. The product-containing fractions were combined and the solvent was removed by rotary evaporation under vacuum to yield the product with 39% yield.

^{1}H NMR (300 MHz; $CDCl_3$): δ 9.22 (s, 1H), 9.04 (s, 1H), 7.94 (d, J = 8.86 Hz, 1H), 7.86 (d, J = 8.14 Hz, 1H), 7.82 (d, J = 8.14 Hz, 1H), 7.78 (d, J = 8.91 Hz, 1H), 7.72-7.63 (m, 5H), 7.50-7.46 (m, 3H), 7.79-7.30 (m, 7H), 7.21-7.13 (m, 5H), 7.04 (d, J = 8.54 Hz, 3H), 6.84 (d, J = 7.58 Hz, 1H), 4.20 (m, 1H), 4.09 (m, 1H), 3.00 (d, J = 8.68, 3H), 2.92 (d, J = 10.29 Hz, 3H), 2.54 (t, J = 7.66 Hz, 2H), 2.13 (m, 3H), 1.55 (m, 2H), 1.32 (m, 2H), 0.92 (t, J = 7.26 Hz, 3H).

^{31}P NMR (121.4 MHz; $CDCl_3$): δ 29.64.

^{13}C NMR (75 MHz; $CDCl_3$): δ 167.4, 167.2, 142.9, 141.5, 141.3 (2C), 139.4, 137.9, 135.6 (2C),135.1, 134.9, 132.6 (2C), 131.2 (2C), 130.5, 130.4, 130.1, 129.7, 129.1, 128.8 (5C), 128.0, 127.9 (2C), 127.8 (2C), 127.6, 127.4, 126.8, 126.1, 126.0, 125.5, 125.2 (2C), 124.9, 123.3, 122.7, 121.2, 120.3 (2C), 119.2, 50.6, 35.1, 34.6, 33.6, 29.7, 22.5, 13.9.

MS (ESI-Q-TOF): calculated for $C_{54}H_{50}N_5O_3P$ = 847.37 Found *m/z* = 848.32 $[M + 1]^+$ and 870.37 $[M + Na]^+$.

Phosphoroamide **7g**

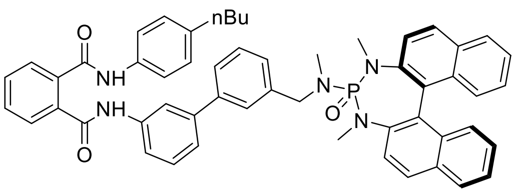

Phosphoroamide (**7g**) was synthetized starting from phosphoroamide (**5g**) and phthalisoimide (**6b**). The crude product was purified by silica gel flash chromatography using CH_2Cl_2/MeOH (98:2 v:v) as eluent. The product-containing fractions were combined and the solvent was removed by rotary evaporation under vacuum to yield the product with 39% yield.

^{1}H NMR (300 MHz; $CDCl_3$): δ 9.56 (s, 1H), 9.21 (s, 1H), 7.91 (d, J = 9.0 Hz, 1H)j, 7.85-7.78 (m, 3H), 7.68 (d, J = 8.9 Hz, 1H), 7.60-7.46 (m, 7H), 7.39-7.29 (m, 9H), 7.20 (m, 2H), 7.16-7.09 (m, 1H), 7.06-6.98 (m, 3H), 4.30 (dd, J = 14.8, 7.5 Hz, 1H), 4.10 (dd, J = 17.9, 7.8 Hz, 1H), 3.01 (d, J = 4.3 Hz, 3H), 2.98 (d, J = 2.9 Hz, 3H), 2.50 (t, J = 7.6 Hz, 1H), 2.18 (d, J = 9.0 Hz, 3H), 1.55 (m, 2H), 1.32 (m, 2H), 0.88 (t, J = 7.3 Hz, 3H).

^{31}P NMR (121.4 MHz; $CDCl_3$): δ 29.25.

^{13}C NMR (75 MHz; $CDCl_3$): 167.04 (2C), 142.44, 140.97 (3C), 140.40, 138.58, 138.32 (2C), 135.27,, 134.64,134.45,132.11, 132.02, 130.73 (2C), 130.65, 129.68, 129.61, 129.18, 128.69 (2C), 128.30, 128.10 (3C), 127.95, 127.59, 127.45, 127.35, 126.89 (2C), 126.56, 125.65 (2C), 125.52, 124.69, 124.43, 122.69, 122.55, 122.16, 119.83 (2C), 118.97, 118.61, 53.06, 35.47 34.54, 34.46, 33.75, 33.04, 21.75, 13.40 .

MS (ESI-Q-TOF): calculated for $C_{54}H_{50}N_5O_3P$ = 847.37 Found *m/z* = 870.30 $[M + Na]^+$.

Phosphoroamide **7h**

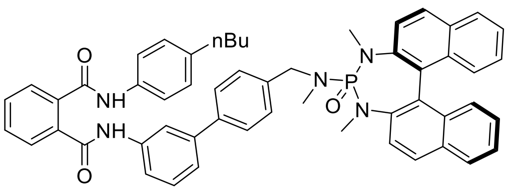

Phosphoroamide (**7h**) was synthetized starting from phosphoroamide (**5h**) and phthalisoimide (**6b**). The crude product was purified by silica gel flash chromatography using CH2Cl2/MeOH (98:2 v:v) as eluent. The product-containing fractions were combined and the solvent was removed by rotary evaporation under vacuum to yield the product with 46% yield.

^{1}H NMR (300 MHz; $CDCl_3$): δ 9.46 (s, 1H), 9.04 (s, 1H), 7.96 (d, J = 8.5 Hz, 2H), 7.89-7.84 (m, 3H), 7.72 (d, J = 8.8 Hz, 2H), 7.63-7.52 (m, 8H), 7.44-7.37 (m, 11H), 7.24 (m, 2H), 7.21-7.12 (m, 1H), 7.08 (d, J = 8.4 Hz, 3H), 4.33 (dd, J = 14.9, 7.3 Hz, 1H), 4.13 (dd, J = 14.8, 10.2 Hz, 1H), 3.06 (d, J = 2.3 Hz, 3H), 3.03 (d, J = 3.7 Hz, 3H), 2.55 (t, J = 7.6 Hz, 2H), 2.21 (d, J = 9.0 Hz, 3H), 1.55 (m, 2H), 1.32 (m, 2H), 0.91 (t, J = 7.3 Hz, 3H).

^{31}P NMR (121.4 MHz; $CDCl_3$): δ 29.22.

^{13}C NMR (75 MHz; $CDCl_3$): δ 167.65, 167.55, 142.93, 141.46, 141.21, 139.77, 137.88, 137.45,, 137.40,135.86,135.12, 134.96, 132.57, 131.48, 131.20, 131.13, 130.05, 129.74, k129.66, 129.14, 128.63 (2C), 128.53, 128.47, 128.29, 128.06, 127.93 (2C), 127.82, 127.34, 127.25, 127.22, 127.12 (2C), 126.12, 125.98, 125.15, 124.89, 123.20, 123.19, 122.89, 122.61, 120.23 (2C), 119.30, 118.88, 53.24,35.97 35.08, 34.98, 34.21, 33.58, 22.29, 13.95 .

MS (ESI-Q-TOF): calculated for $C_{54}H_{50}N_5O_3P$ = 847.37 Found *m/z* = 848.32 $[M+1]^+$ and 870.31 $[M+Na]^+$.

<u>*6.1. Synthesis of compound* **7i:**</u>

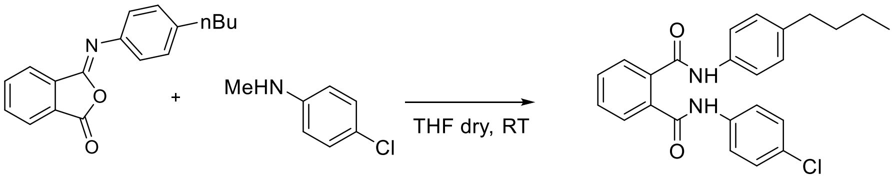

6b **3i** **7i**

Compound (**6b**) (1 eq, 1.43 mmol, 400 mg) and amine (**3i**) (1.1 eq, 1.57 mmol, 200 mg) were dissolved in dry THF (15 mL). After 24 h refluxing, the mixture was cooled to RT and 5.0 mL of HCl 5% solution were added. The crude was then diluted with ethyl acetate (30 mL), and the organic layer was washed with brine, dried over Na_2SO_4, filtered and concentrated by rotary evaporation. The residue was purified by silica gel flash chromatography using ethyl acetate/CH_2Cl_2 (9:1 v:v) as eluent furnishing the desired product in 42% yield.

^{1}H NMR (300 MHz; $CDCl_3$): 8.94 (s, 1H), 8.76 (s, 1H), 8.03, 7.59-7.09 (m, 13H), 2.60 (m, 2H), 1.59 (m, 2H), 1.37 (m, 2H), 0.92 (t, J = 6.9 Hz, 3H).

7. General procedure F: synthesis of 3,5-bisubstituted pyridines 13a-b

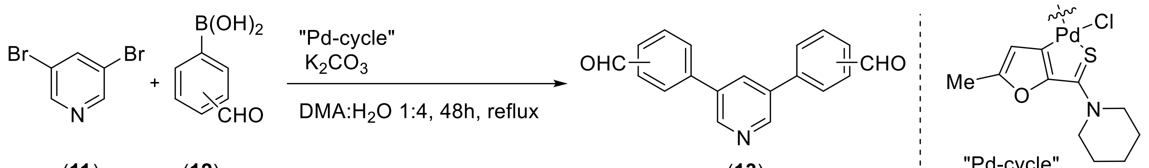

(11) (12) (13)

"Pd-cycle" catalyst was synthetized according to a procedure reported in literature.[3]

Pd catalyst (0.02 eq, 0.1 mmol, 76 mg) and 3,5-dibromopyridine **11** (1 eq, 5.54 mmol, 1.29g) were dissolved in 8.25 mL of dimethylacetamide. The mixture was stirred for 15 min, then solid K_2CO_3 (4 eq, 21.82 mmol, 3.0 g), the desired boronic acid **12** (2.2 eq, 12 mmol, 1.8 g) and 2.75 ml of water were added and the mixture was gently refluxed for 48h. After that time, the mixture was cooled at RT and diluted with 30 ml of $CHCl_3$. The mixture was then washed with water (15 ml) and HCl 3% solution (15 ml). The organic layer was collected, dried over Na_2SO_4, filtered and concentrated by rotary evaporation. The residue was purified by silica gel flash chromatography using $MeOH/CH_2Cl_2$ (1:99 v:v) as eluent furnishing the desired product

Compound **13a**

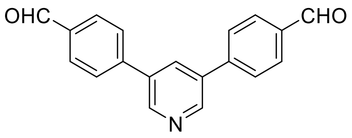

The product was obtained starting from (4-formylphenyl)boronic acid **12a** in 65% yield.

^{1}H NMR (300 MHz; $CDCl_3$): δ 10.14 (s, 2H), 8.01 (d, J = 9.0 Hz, 4H), 7.88 (s, 1H), 7.81 (d, J = 9.0 Hz, 4H), 7.69 (d, J = 6.0 Hz, 2H), 7.64-7.59 (m, 1H).

MS (ESI+): calculated for $C_{19}H_{13}NO_2$ = 287.09. Found *m/z* = 288.44 $[M + 1]^+$.

Compound **13b**

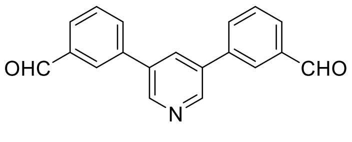

The product was obtained starting from (3-formylphenyl)boronic acid **12b** in 52% yield.

^{1}H NMR (300 MHz; $CDCl_3$): δ 10.14 (s, 2H), 8.92 (s, 2H), 8.17 (d, *J* = 7.1 Hz, 2H), 7.95 (t, *J* = 8.7 Hz, 3H), 7.71 (t, *J* = 7.8 Hz, 2H), 7.26 (s, 2H).

^{13}C NMR (75 MHz, DMSO) δ 191.7, 149.0, 148.1, 147.5, 138.4, 137.2, 135.5, 134.5, 132.9, 130.0, 129.9, 129.8, 129.5, 127.9, 123.7.

MS (ESI+): calculated for $C_{19}H_{13}NO_2$ = 287.09. Found *m/z* = 288.45 $[M + 1]^+$.

8. General procedure G: synthesis of bis(N-methylamino)-pyridines 14a-b

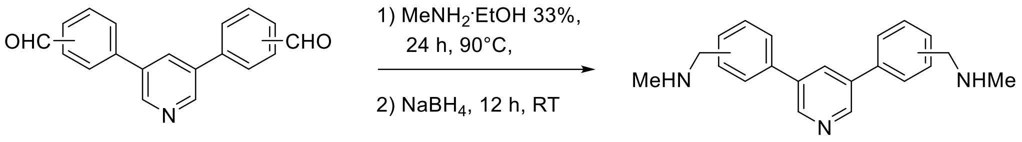

The desired bisamino-pyridine **13a** or **13b** (1 eq, 0.57 mmol, 165 mg), methylamine (33% EtOH solution, 10 eq, 5.74 mmol, 0.715 mL) and molecular sieves (ca 30 mg) were introduced in a screw cap vial and the mixture was heated at 80°C for 24h. After cooling to RT, the mixture was diluted with 3 ml of EtOH and cooled to 0 °C. $NaBH_4$ (3 eq, 1.72 mmol, 65 mg) was added and the resultant mixture allowed warming to RT and reacting for 24 h. The mixture was then quenched with aq. NH_4OH 5% (4 mL) and the solvent was concentrated by rotary evaporation. The obtained aqueous layer was extracted with CH_2Cl_2 (2 x 5 mL). The combined organic extracts were dried over Na_2SO_4, filtered and the filtrate was concentrated by rotary evaporation. The residue was purified by silica gel flash chromatography using MeOH/CH_2Cl_2 (3:7: v:v) + Et_3N 1% as eluent furnishing the desired product.

Compound **14a**

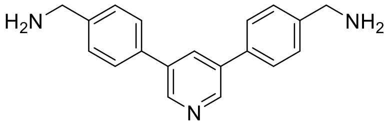

The product was obtained starting from compound **13a** in 82% yield.

^{1}H NMR (300 MHz; $CDCl_3$): δ 8.80 (s, 2H), 8.01 (s, 1H), 7.61 (d, J = 8.1 Hz, 4H), 7.46 (d, J = 7.9 Hz, 4H), 4.12 (br, 2H), 3.88 (s, 4H), 2.52 (s, 6H).

MS (ESI+): calculated for $C_{21}H_{23}N_3$ = 317.19. Found m/z = 318.44 $[M + 1]^+$.

Compound **14b**

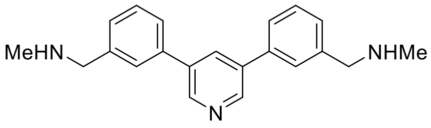

The product was obtained starting from compound **13b** in 45% yield.

^{1}H NMR (300 MHz; $CDCl_3$): δ 8.79 (s, 2H), 8.08 (s, 1H), 7.65 (s, 2H), 7.54 (d, J = 7.5 Hz, 2H), 7.44 (t, J = 7.5 Hz, 2H), 7.37 (d, J = 7.6 Hz, 2H), 4.07 (br, 2H), 3.87 (s, 4H), 2.49 (s, 6H).

^{13}C NMR (75 MHz, DMSO) δ 146.4, 146.3, 139.1, 137.4, 135.8, 132.4, 128.7, 128.6, 128.4, 127.8, 127.7, 126.8, 126.7, 125.6, 125.5, 54.7, 34.8.

9. General procedure H: synthesis of bisphosphoroamides 15a-b:

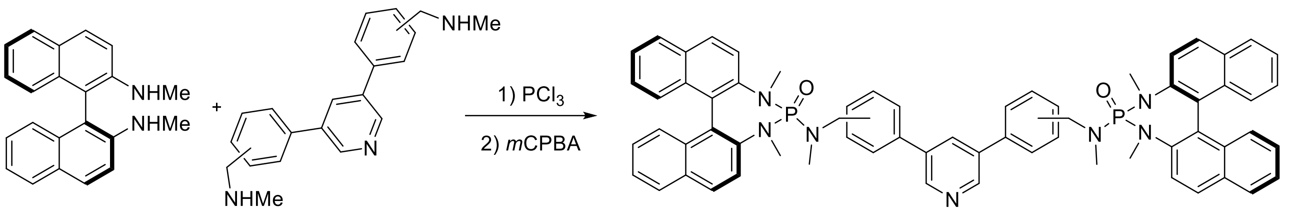

1 **14** **15**

N,N'-dimethyl-1,1'-binaphthyl-2,2'-diamine (**1**) (1 eq, 1.66 mmol, 520 g) and Et_3N (5 eq, 3.96 mmol, 0.552 mL) were dissolved in dry THF (18 mL). The homogeneous mixture was cooled to 0 °C then PCl_3 (6.3 eq, 10.45 mmol, 0.915 mL) was added dropwise via syringe whereupon a colorless precipitate formed immediately. The reaction mixture was stirred at 0 °C for 1.5 h, then was allowed to warm to room temperature and stirred for another 3 h. The volatiles were removed under high vacuum (room temperature, 0.5mmHg) and Et_2O (20.0 mL) was added via syringe, then the mixture was stirred for 5 min. After that the supernatant was canula-filtered into another round bottom flask. The remaining precipitate in the reaction flask was washed again with Et_2O (20 mL) and filtered (2 times). The volatiles were removed under high vacuum (room temperature, 0.5 mmHg) to afford a light yellow solid. The solid was then dried for 12 h at reduced pressure (room temperature, 0.5 mmHg) to give a white solid foam (**2**). Dry CH_2Cl_2 (10 mL) was added via syringe and the mixture was cooled to 0 °C. To this solution, a mixture of Et_3N (5 eq, 3.96 mmol, 0.552 mL) and the desired bis(*N*-methylamino)-pyridine (**14**) (1 eq, 0.79 mmol, 250 mg) dissolved in dry CH_2Cl_2 (12 mL) were added. The reaction mixture was allowed to warm to room temperature and stirred for 24 h. A solution of freshly crystallized *m*CPBA (70%) (3 eq, 2.37 mmol, 582 mg) dissolved in 2 mL of CH_2Cl_2 was then added and the mixture was stirred for 72 h. After a quench with 10 mL of NH_4Cl saturated aqueous solution, the phases were separated and aqueous layer was washed with CH_2Cl_2 (10 mL). The combined organic extracts were washed with brine, dried over Na_2SO_4, filtered and concentrated by rotary evaporation. The crude residue was purified by silica gel flash chromatography using hexane:ethyl acetate (2:8 v:v) as eluent to yield the desired product as pale yellow solid.

Compound **15a**

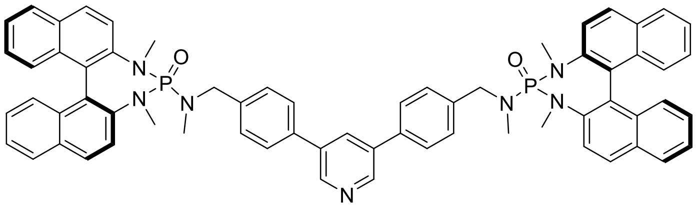

The product was obtained starting from compound **14a** in 52% yield.

^{1}H NMR (300 MHz; $CDCl_3$): δ 8.88 (s, 2H), 8.13 (s, 1H), 7.98-7.86 (m 10H), 7.79-7.65 (m, 8H), 7.58-7.51 (m, 4H), 7.50-7.43 (m, 2H), 7.40-7.34 (m, 5H), 7.25-7.16 (m, 6H), 7.13 (d, 2H), 7.07-7.04 (m, 3H), 4.41 (dd, *J* = 14.9, 7.3 Hz, 1H), 4.24 (dd, *J* = 14.1, 11.0 Hz, 1H), 3.09 (d, *J* = 9.4 Hz, 12H), 2.23 (d, *J* = 8.8 Hz, 6H).

^{13}C NMR (192.5 MHz; $CDCl_3$): δ 146.3, 142.7, 141.3, 138.8, 138.8, 136.4, 132.8, 132.5, 132.4, 131.1 (2C), 129.6, 129.1 (2C), 128.7, 128.0 (2C), 127.8, 127.8, 127.3 (2C), 127.2 (2C), 126.1, 125.9, 125.2, 124.8, 123.2, 122.6, 53.2, 36.1, 35.0, 34.2, 29.5.

^{31}P NMR (121.4 MHz; $CDCl_3$): δ 29.15.

MS (ESI-): calculated for $C_{65}H_{57}N_7O_2P_2$ = 1029.40 Found *m/z* = 521.45 [M - 1]$^-$.

Compound **15b**

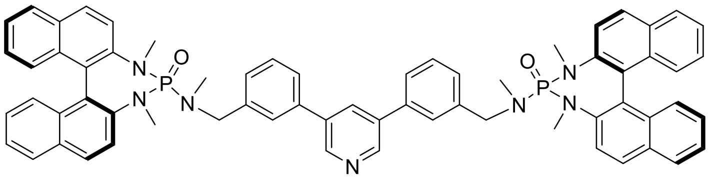

The product was obtained starting from compound **14b** in 56% yield.

^{1}H NMR (600 MHz; $CDCl_3$): δ 8.89 (s, 2H), 8.15 (s, 1H), 8.10-8.05 (m, 4H), 8.00 (d, *J* = 8.9 Hz, 1H), 7.96 (d, *J* = 7.8 Hz, 1H), 7.90 (d, *J* = 8.2 Hz, 1H), 7.85 (t, *J* = 8.4 Hz, 2H), 7.78 (d, *J* = 8.8 Hz, 1H), 7.69 (s, 1H), 7.63 (t, *J* = 7.5, 1H), 7.56-7.48 (m, 6H), 7.45-7.36 (m, 6H), 7.24-7.22 (m, 5H) 7.19-7.16 (m, 1H), 7.08 (d, *J* = 8.5 Hz, 2H), 4.48 (dd, *J* = 14.3, 6.5 Hz, 2H), 4.27 (dd, *J* = 14.6, 10.4 Hz, 2H), 3.10 (d, *J* = 8.0 Hz, 6H), 3.09 (d, *J* = 6.7 Hz, 6H), 2.25 (d, *J* = 8.3 Hz, 6H).

^{13}C NMR (192.5 MHz; $CDCl_3$): δ 147.0 (2C), 142.8 (2C), 141.5 (2C), 139.8 (2C), 137.9 (2C), 136.7 (2C), 133.0, 132.7 (2C), 132.5 (2C), 132.0 (4C), 131.2 (4C), 129.8 (2C), 129.4 (2C), 129.3 (2C), 129.2 (2C), 128.2 (2C), 128.0 (2C), 127.9 (2C), 127.4 (2C), 127.3 (2C), 126.3 (2C), 126.1 (4C), 125.3 (2C), 125.0 (2C), 123.3 (2C), 122.7 (2C), 53.7 (2C), 36.2 (2C), 35.1 (2C), 34.4 (2C).

^{31}P NMR (121.4 MHz; $CDCl_3$): 28.88.

10. General procedure for allylation of aldehydes

Phosphoramide catalyst (0.1 eq or 0.05 eq) was dissolved in CH_2Cl_2 (1.5 mL) under N_2. To this solution was added allyltributyltin (1.2 eq, 0.54 mmol, 169 μL) and the resulting mixture was cooled to -78°C (bath temperature). Then fresh distilled $SiCl_4$ (2 eq, 0.9 mmol, 104 μl) was added followed by the aldehyde (1 eq, 0.45 mmol). The resulting mixture was allowed to stir at -78 °C (bath temperature) for 6 h whereupon the cold reaction mixture was rapidly poured into a stirring solution of 1/1 sat. aq. KF/1.0 M KH_2PO_4 (5 mL). This biphasic mixture was stirred vigorously for 12 h, then, diluted with CH_2Cl_2 (10 mL), the layers were separated and aqueous one was washed with CH_2Cl_2 (3 x 10 mL). The combined organic extracts were dried over Na_2SO_4, filtered and the filtrate was concentrated by rotary evaporation under vacuum. The residue was purified by silica gel flash chromatography using hexane/ethyl acetate (9:1, v:v) as eluent with a plug of solid anhydrous KF (15 mm) on the top of the column . The product-containing fractions were combined and the solvent was removed by rotary evaporation under vacuum to yield the desired allylic alcohol.

1-phenylbut-3-en-1-ol (8a)

^1H NMR (300 MHz; $CDCl_3$): 7.38-7.27 (m, 5H), 5.89-5.80 (m, 1H), 5.21-5.15 (m, 2H), 4.75 (t, J = 5 Hz, 1H), 2.62-2.46 (m, 2H).

HPLC: t_R (R)=9.51 min ; t_R (S)=10.62 min (Chiralcel OD, hexane : *i*-PrOH 95:5, 0.800 mL/min, 15 bar, 210.8 nm). For example: HPLC trace of product (entry 5, table 1).

1-(4-chlorophenyl)but-3-en-1-ol (8b)

^1H NMR (300 MHz; $CDCl_3$): δ 7.44 – 7.21 (m, 4H), 5.94-5.66 (m, 1H), 5.20 (d, J = 3.6 Hz, 2H), 4.74 (dd, J = 7.6, 5.2 Hz, 2H), 2.58-2.42 (m, 2H).

HPLC: t_R (S)=32.04 min ; t_R (S)=36.7 min (Chiralcel OJ-H, hexane : *i*-PrOH 98:2, 0.800 mL/min, 30 bar, 230 nm).

1-(4-methoxyphenyl)but-3-en-1-ol (8c)

^1H NMR (300 MHz; $CDCl_3$):

7.31 (d, J = 8.7 Hz, 2H), 6.91 (d, J = 8.7 Hz, 2H), 5.94-5.72 (m, 1H), 5.26-5.09 (m, 2H), 4.72 (t, J = 6.5 Hz, 1H), 3.83 (s, 3H), 2.55.2.50 (m, 2H).

HPLC: t_R (R)=25.97 min ; t_R (S)=30.30 min (Chiralcel OD, hexane : *i*-PrOH 98:2, 0.800 mL/min, 15 bar, 210.8 nm

1-phenylbut-3-en-1-ol (8a)

CHIRALCEL OD Hex:iPrOH 95:5 0.800 ml/min 15.5 bar

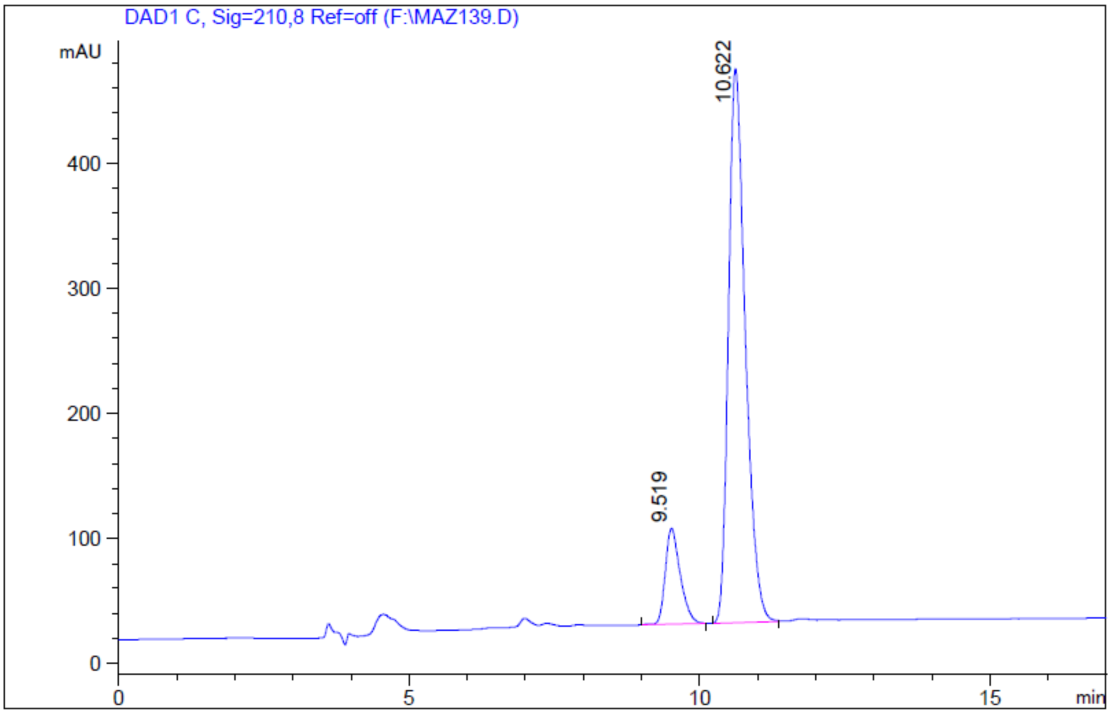

Signal 1: DAD1 C, Sig=210,8 Ref=off

Peak #	RT [min]	Type	Width [min]	Area	Area %	Name
1	9.519	BB	0.269	1373.796	13.351	
2	10.622	BB	0.304	8915.929	86.649	

1-(4-chlorophenyl)but-3-en-1-ol (8b)

OJ-H Hex:IPA 98:2 0.8 ml/min 30bar

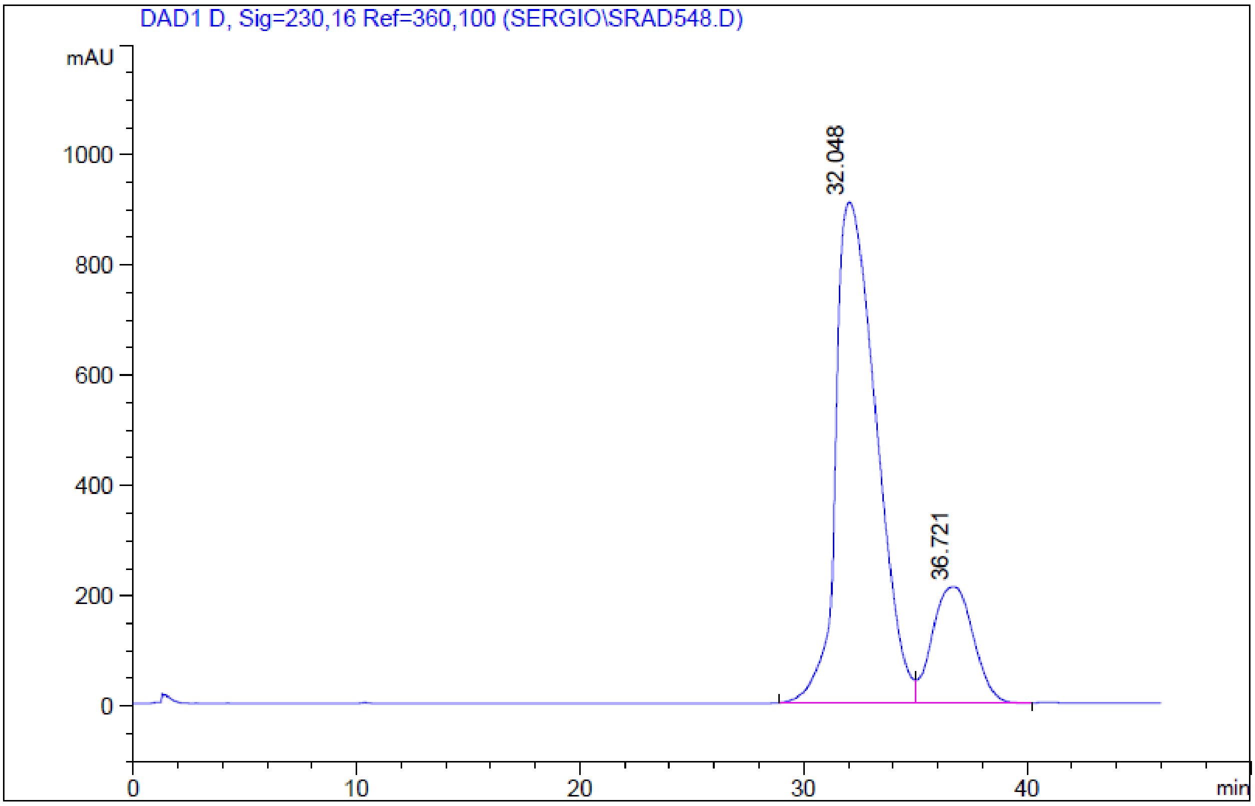

Signal 1: DAD1 D, Sig=230,16 Ref=360,100

Peak #	RT [min]	Type	Width [min]	Area	Area %	Name
1	32.048	BV	1.479	114761.477	81.057	
2	36.721	VB	2.008	26819.705	18.943	

1-(4-methoxyphenyl)but-3-en-1-ol (8c)

OD Hex:IPA 98:2 0.8 ml/min 15bar

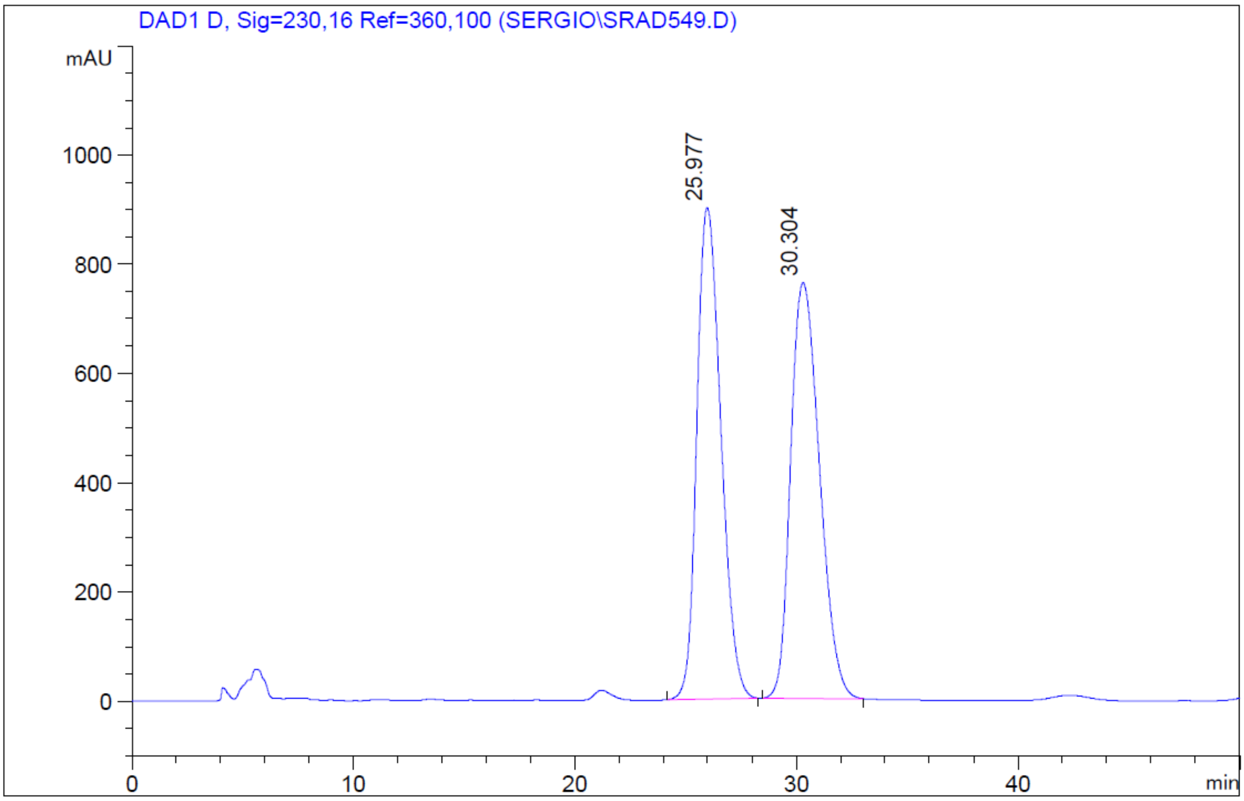

Signal 1: DAD1 D, Sig=230,16 Ref=360,100

Peak #	RT [min]	Type	Width [min]	Area	Area %	Name
1	25.977	BB	1.188	68067.445	49.661	
2	30.304	BB	1.421	68996.727	50.339	

11. NMR Studies

11.1 Dependence of the NH chemical shifts of free catalyst 7c on the concentration

A NMR tube was charged with a solution of ligand **7c** in CD_2Cl_2 at different concentrations. Experiments were conducted operating at 300 MHz at RT. The variation of the chemical shift (^1H-NMR of the NH groups and ^{31}P-NMR are reported in table S-1.

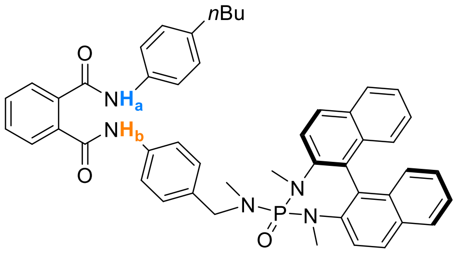

Entry	[mM]	H_a ppm	$\Delta_{(ppm)}H_a$	H_b ppm	$\Delta_{(ppm)}H_b$	P (ppm)	$\Delta_{(ppm)}P$
1	30	9.4283	-	9.1271	-	29.1511	-
2	15	9.2162	0.212	8.8893	0.238	29.1382	0.0129
3	7.5	9.0263	0.189	8.6805	0.209	29.1319	0.0063
4	3.75	8.8767	0.149	8.5198	0.160	29.1352	0.0033
5	1.875	8.7738	0.102	8.4088	0.111	29.1327	0.0035
6	0.937	8.7083	0.065	8.3403	0.069	29.1252	0.0075
7	0.45	8.6816	0.027	8.3094	0.031	29.1231	0.0021

Table S-1

Δ(ppm) between 3.75 mM and 1.875 mM is about 0.1 ppm. So 3.75 mM was selected as final concentration for further experiments.

11.2 NMR Studies of catalyst 7c in the presence of $SiCl_4$ at RT

Under nitrogen, a NMR tube was charged with a solution of catalyst **7c** (2 eq, 0.03 mmol) dissolved in 0.4 mL of CD_2Cl_2 followed by a solution of $SiCl_4$ (1 eq, 0.015 mmol) diluted in 0.4 mL of CD_2Cl_2. NMR acquisition at RT showed the formation of compound **9**, that was isolated by chromatographic purification using ethyl acetate/methanol (99:1 v:v) as eluent.

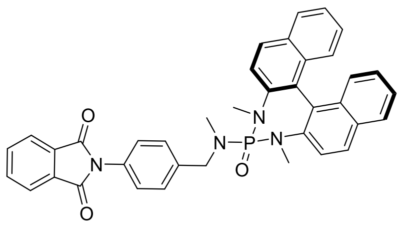

^{1}H NMR (300 MHz; $CDCl_3$): δ 7.99-7.94 (m, 3H), 7.87 (d, *J* = 8.5 Hz, 2H), 7.81-7.76 (m, 3H), 7.53 (d, *J* = 8.3 Hz, 2H), 7.46 (d, *J* = 8.4 Hz, 2H), 7.40-7.33 (m, 4H), 7.22-7.18 (m, 2H), 7.15-7.12 (m, 1H), 7.05 (d, *J* = 8.3 Hz, 1H), 4.40 (dd, *J* = 14.7, 7.5 Hz, 1H), 4.17 (dd, *J* = 14.1, 7.6 Hz, 1H), 3.08 (d, *J* = 9.1 Hz, 6H), 2.22 (d, *J* = 8.9 Hz, 3H).
^{31}P NMR (121.4 MHz; CDCl3): 29.03.

11.3 NMR Studies of catalyst 7c in the presence of $SiCl_4$ at -50°C

Under nitrogen, a NMR tube was charged with a cooled solution of catalyst **7c** (2 eq, 0.03 mmol) dissolved in 0.4 mL of CD_2Cl_2 followed by a cooled solution of $SiCl_4$ (1 eq, 0.015 mmol) diluted in 0.4 mL of CD_2Cl_2. NMR acquisitions were performed at -50 °C.

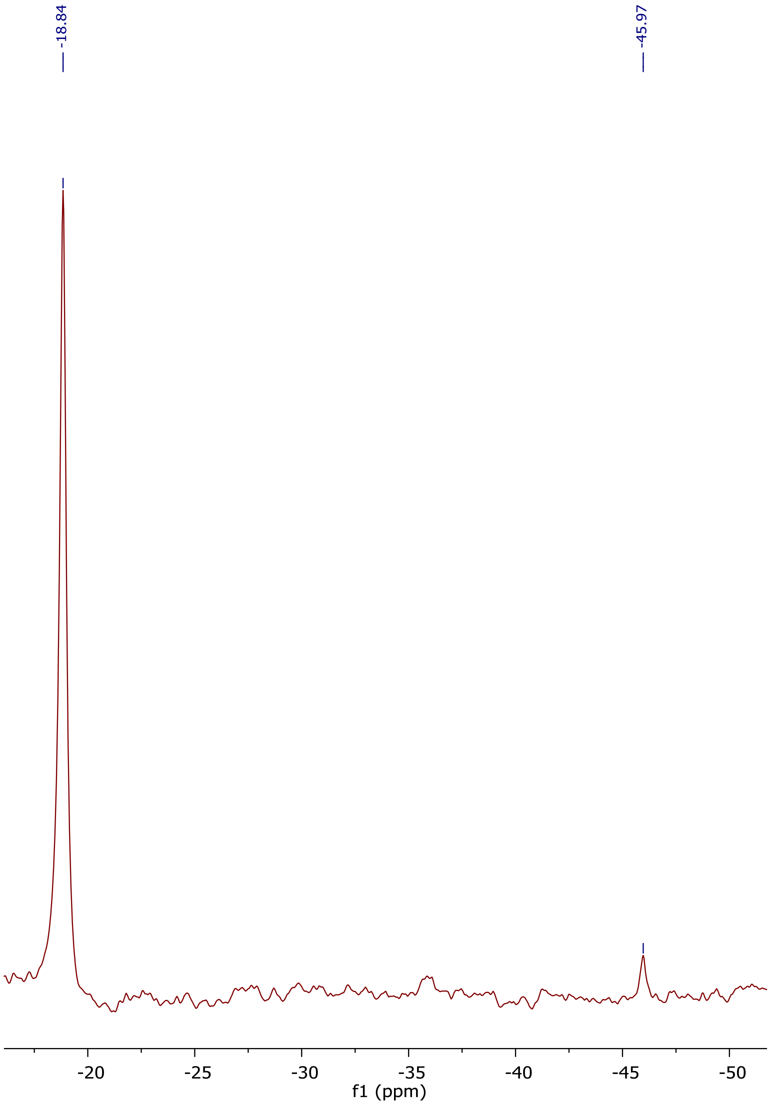

BBI; ^{29}SI; zgig; 3.75 mM; 223 K (-50°C); CD_2Cl_2

11.4 Investigation on solubility of Py_2:$SiCl_4$ complexes

In order to investigate the complexation of substituted pyridines with $SiCl_4$, preliminary studies on the solubility of the complexes were conducted. A NMR tube was charged with a solution of 4-substituted pyridine (2 eq) and $SiCl_4$ (1 eq) in CD_2Cl_2 (final concentration in $SiCl_4$ 0.3 M, 0.15 M or 0.075 M) under nitrogen. The reaction was allow to stay for 15 min, then the formation of precipitate was investigated by naked eye.

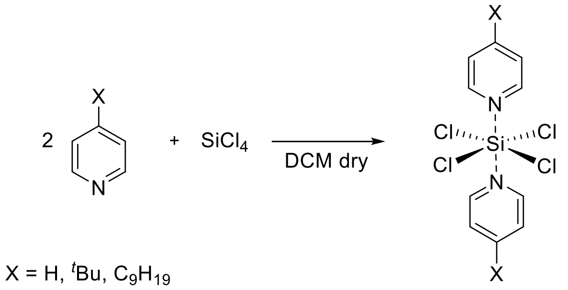

	[Py_2SiCl_4]			[$(4\text{-}^tBu\text{-}Py)_2SiCl_4$]			[$(4\text{-}C_9H_{19}\text{-}Py)_2SiCl_4$]		
	0.3 M	0.15M	0.075 M	0.3 M	0.15M	0.075 M	0.3 M	0.15M	0.075 M
25°C	■ (red)	■ (yellow)	■ (green)	■ (green)	■ (green)	■ (green)	■ (green)	■ (green)	■ (green)
-20°C	■ (red)	■ (red)	■ (green)	■ (green)	■ (green)	■ (green)	■ (green)	■ (green)	■ (green)
-50°C	■ (red)	■ (red)	■ (yellow)	■ (red)	■ (green)	■ (green)	■ (red)	■ (green)	■ (green)
-78°C	■ (red)	■ (red)	■ (yellow)	■ (red)	■ (red)	■ (green)	■ (red)	■ (yellow)	■ (green)

■ (red) Precipitate formed. ■ (yellow) Cloudy solution. ■ (green) No precipitate observed.

$(4\text{-}C_9H_{19}\text{-}Py)_2SiCl_4$ was selected for ^{29}Si NMR investigations

BBI; ^{29}SI; zgig; [(4-C_9H_{19}-Py)$_2$$SiCl_4$]; 0.075M; 210 K (-63°C); CD_2Cl_2

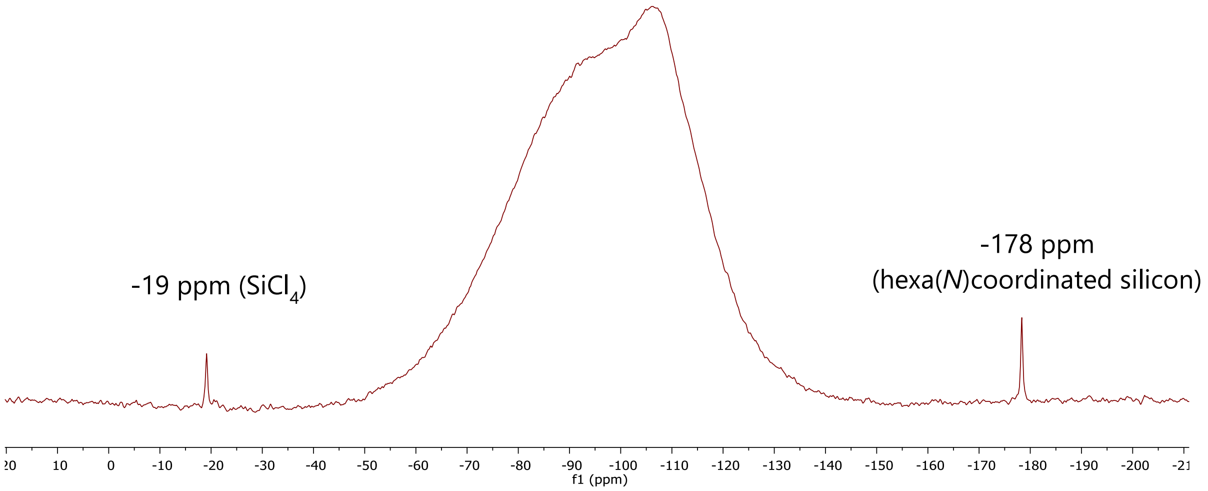

REFERENCES

1. Denmark, S. E.; Rossi, S.; Webster, M. P.; Wang, H., Catalytic, enantioselective sulfenylation of ketone-derived enoxysilanes. *J Am Chem Soc* **2014,** 136, (37), 13016-28.
2. Pignataro, L.; Carboni, S.; Civera, M.; Colombo, R.; Piarulli, U.; Gennari, C., PhthalaPhos: chiral supramolecular ligands for enantioselective rhodium-catalyzed hydrogenation reactions. *Angew. Chem. Int. Ed* **2010,** 49, (37), 6633-6637.
3. Nojima, Y.; Nonoyama, M.; Nakajima, K.; Cyclopalladation of N-(p-thiotoluoyl)pyrrolidine and -piperidine *Polyhedron* **1996**, *15*, 3795-3809.

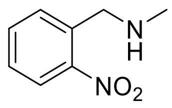

NO2
N
H

3a

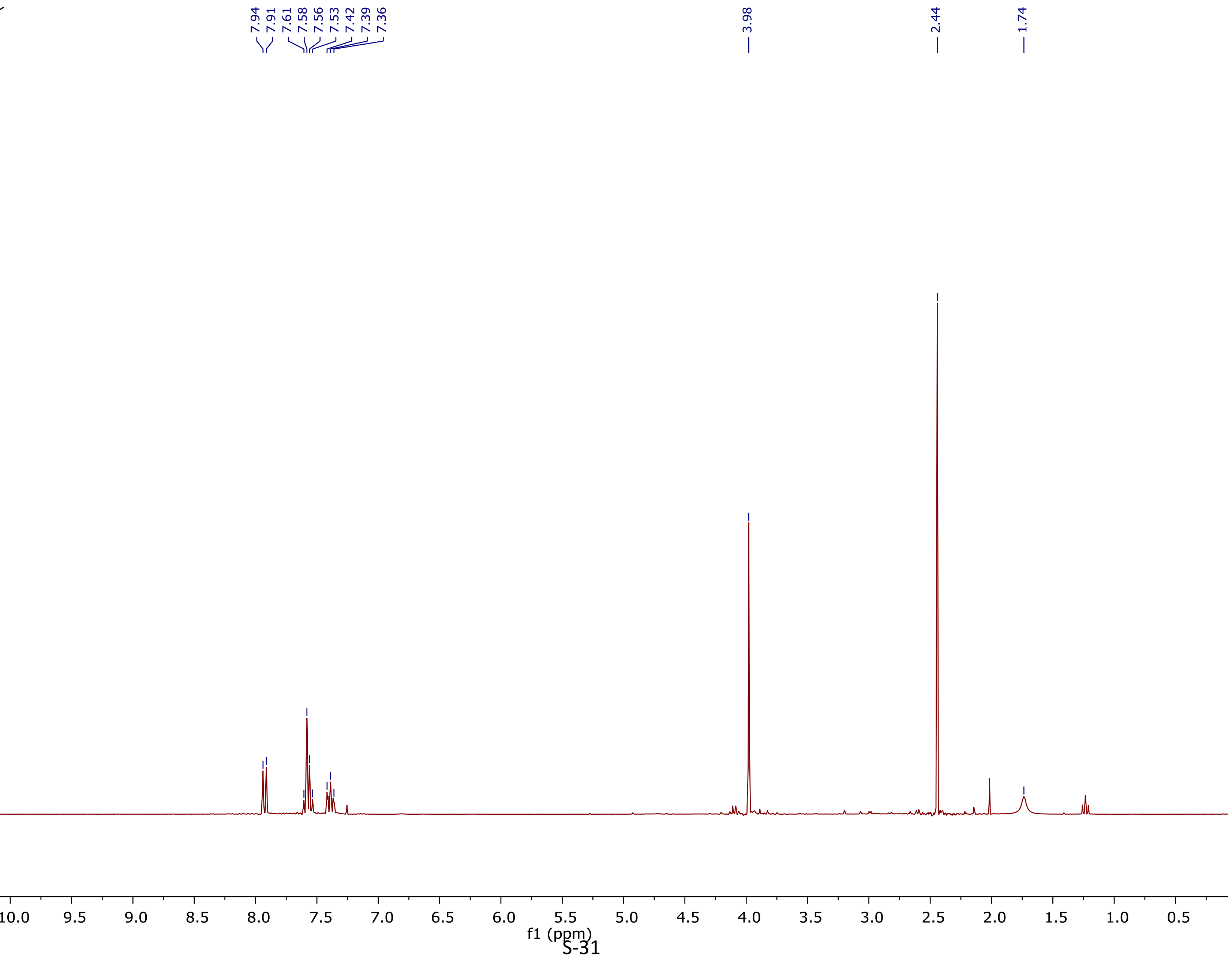

7.94
7.91
7.61
7.58
7.56
7.53
7.42
7.39
7.36
3.98
2.44
1.74
1.0
10.5
10.0
9.5
9.0
8.5
8.0
7.5
7.0
6.5
6.0
5.5
5.0
4.5
4.0
3.5
3.0
2.5
2.0
1.5
1.0
0.5
f1 (ppm)

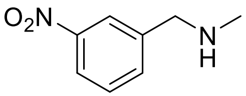
O2N
N
H

3b

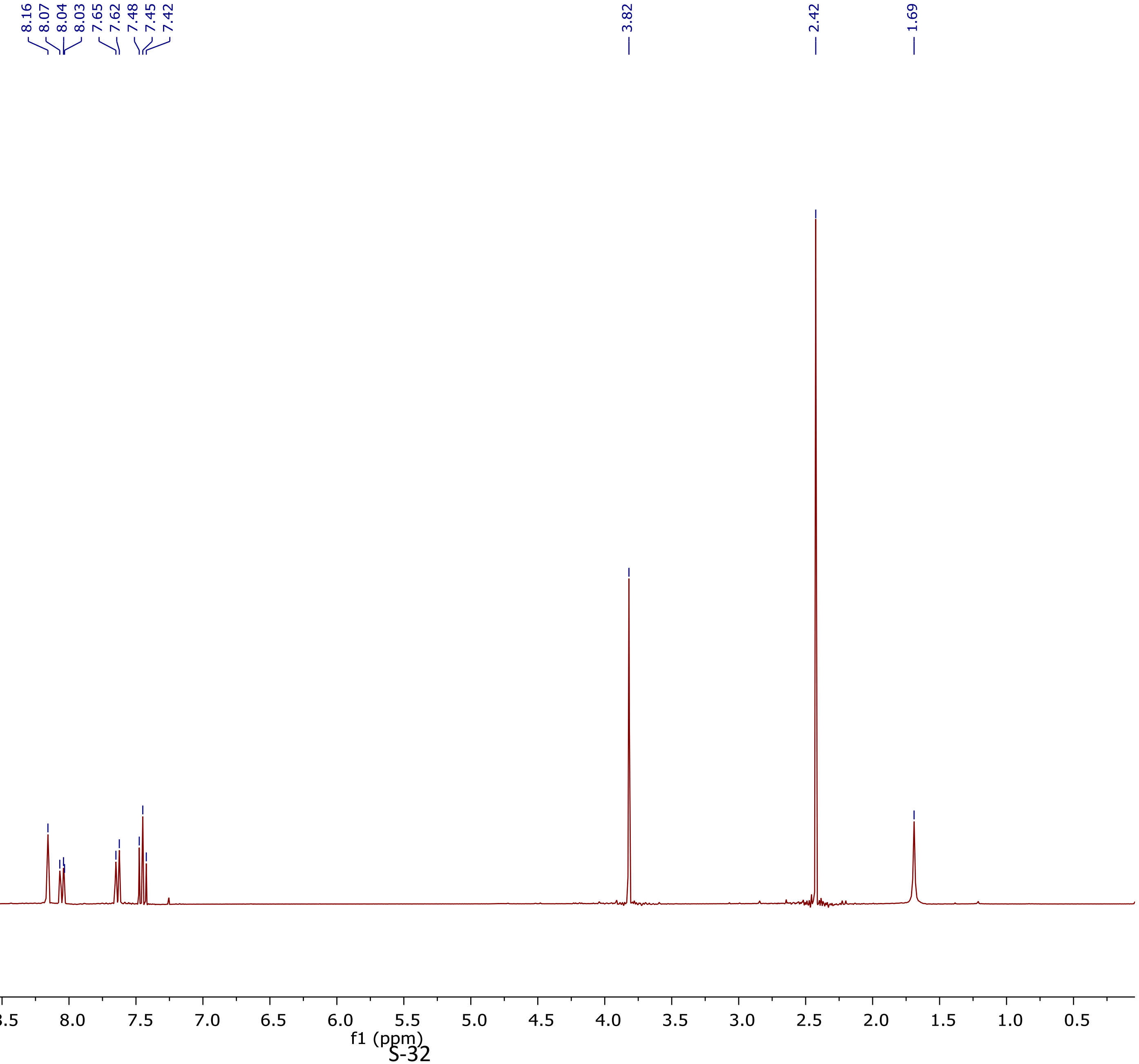
8.16
8.07
8.04
8.03
7.65
7.62
7.48
7.45
7.42
3.82
2.42
1.69
11.0
10.5
10.0
9.5
9.0
8.5
8.0
7.5
7.0
6.5
6.0
5.5
5.0
4.5
4.0
3.5
3.0
2.5
2.0
1.5
1.0
0.5
f1 (ppm)

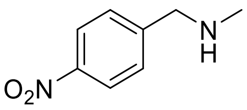

O_2N
N
H

3c

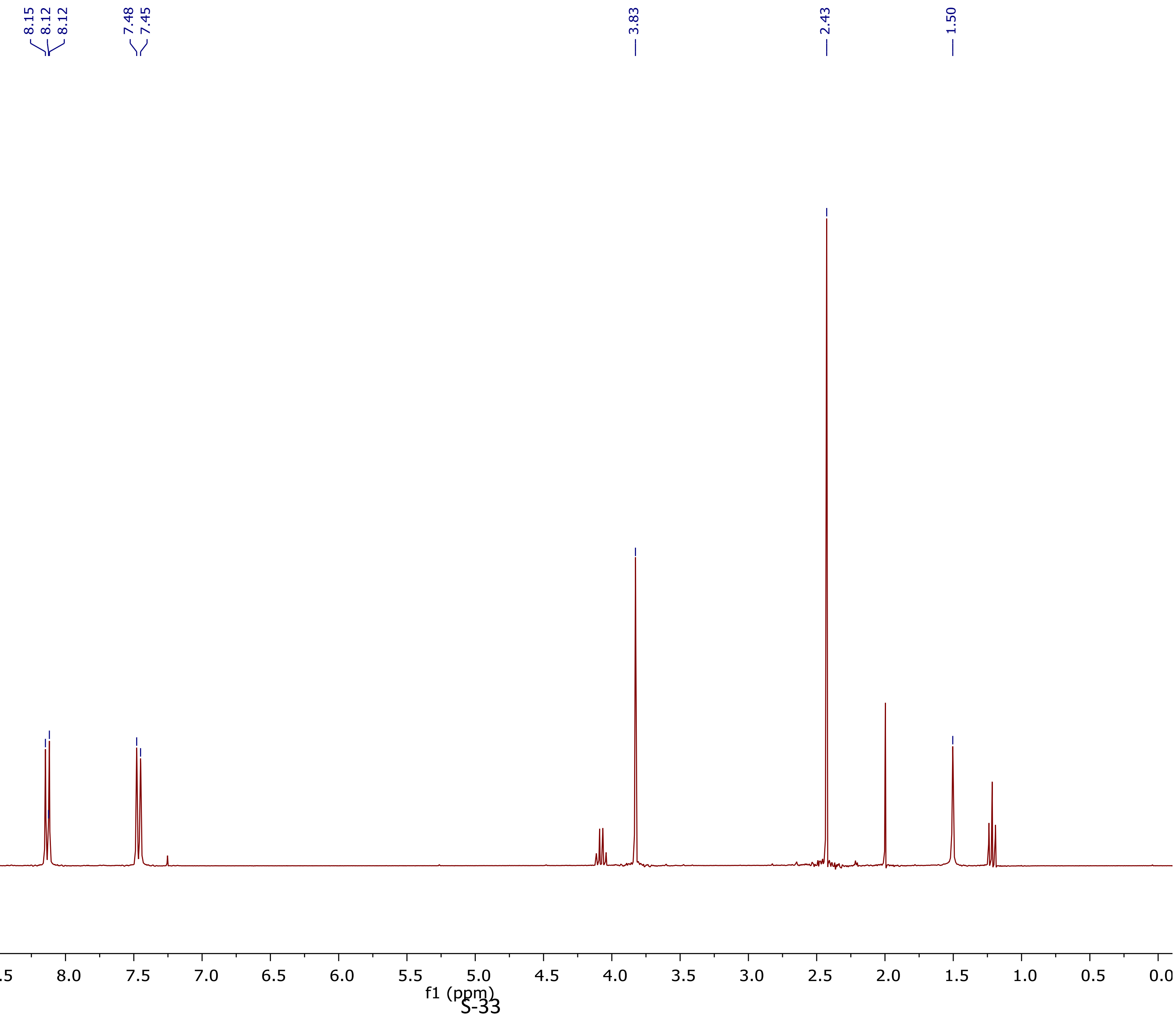

8.15
8.12
8.12
7.48
7.45
3.83
2.43
1.50
10.0
9.5
9.0
8.5
8.0
7.5
7.0
6.5
6.0
5.5
5.0
4.5
4.0
3.5
3.0
2.5
2.0
1.5
1.0
0.5
0.0
f1 (ppm)

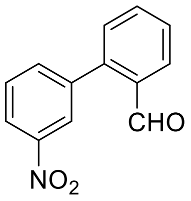

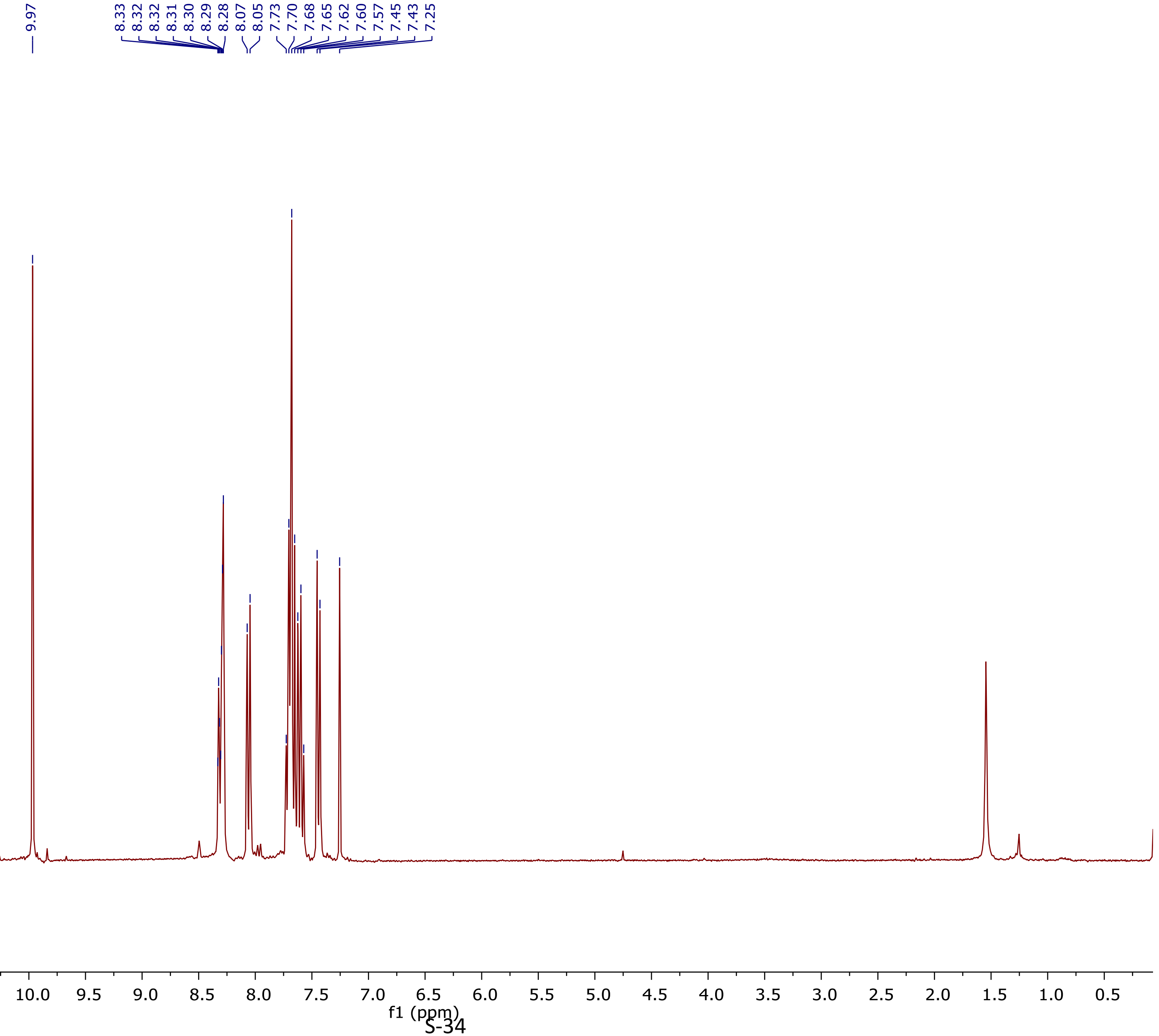

9.97
8.33
8.32
8.32
8.31
8.30
8.29
8.28
8.07
8.05
7.73
7.70
7.68
7.65
7.62
7.60
7.57
7.45
7.43
7.25
3.0
12.5
12.0
11.5
11.0
10.5
10.0
9.5
9.0
8.5
8.0
7.5
7.0
6.5
6.0
5.5
5.0
4.5
4.0
3.5
3.0
2.5
2.0
1.5
1.0
0.5
f1 (ppm)

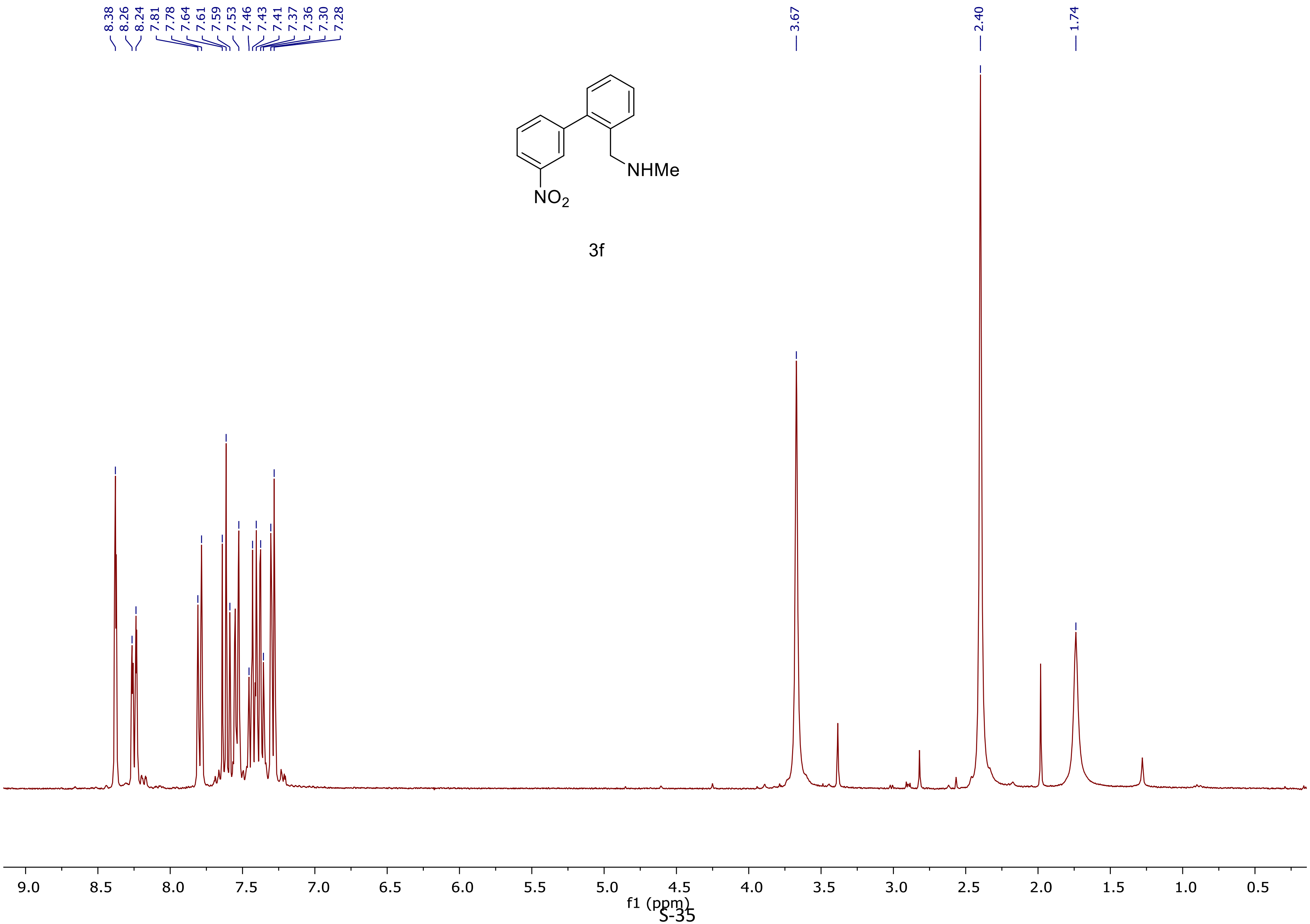
8.38
8.26
8.24
7.81
7.78
7.64
7.61
7.59
7.53
7.46
7.43
7.41
7.37
7.36
7.30
7.28
3.67
2.40
1.74
NHMe
NO_2
9.0
8.5
8.0
7.5
7.0
6.5
6.0
5.5
5.0
4.5
4.0
3.5
3.0
2.5
2.0
1.5
1.0
0.5
f1 (ppm)

3f

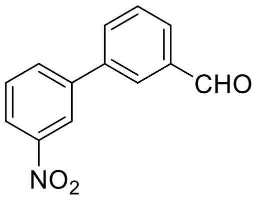
CHO
NO2

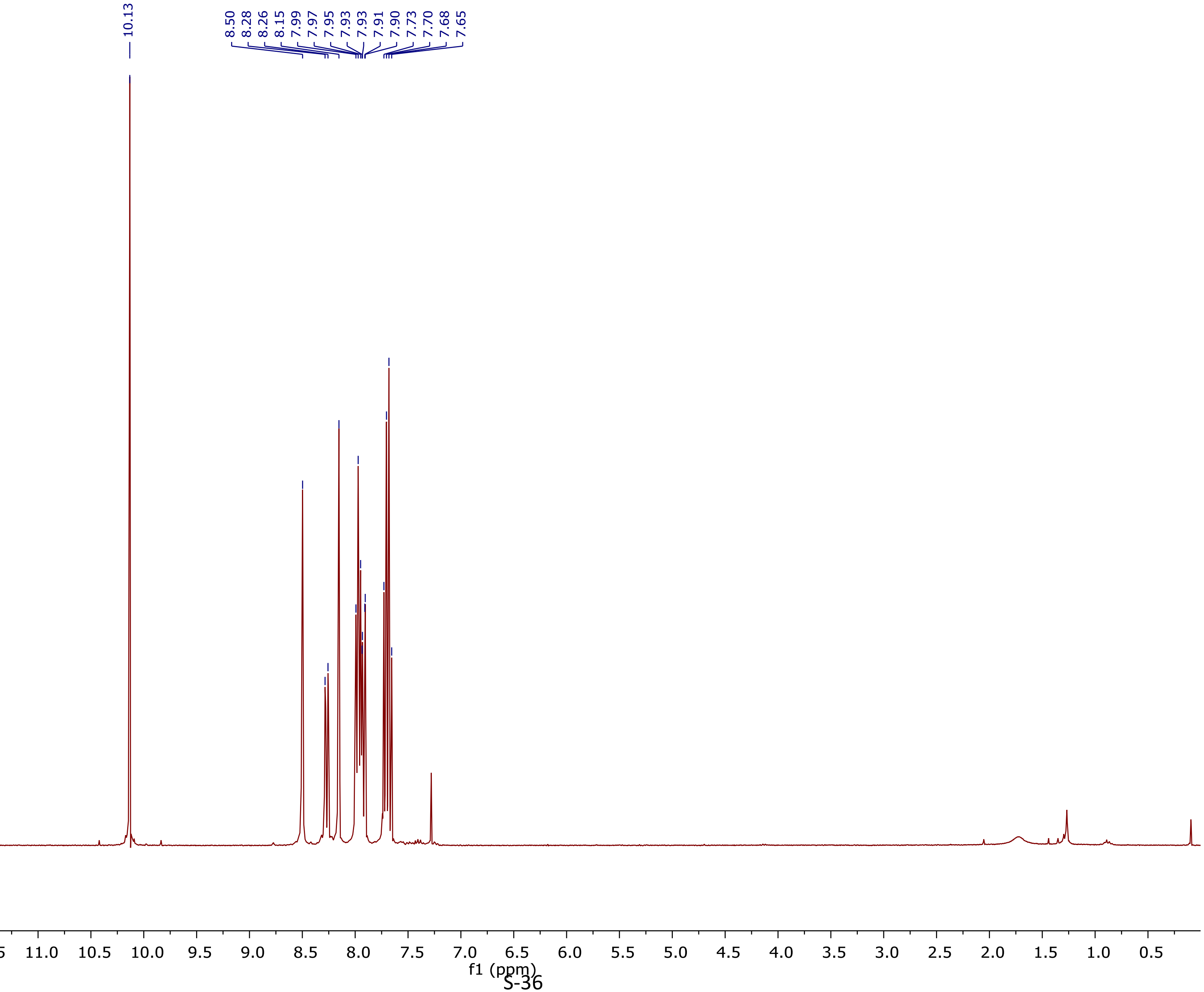
10.13
8.50
8.28
8.26
8.15
7.99
7.97
7.95
7.93
7.93
7.91
7.90
7.73
7.70
7.68
7.65
13.0 12.5 12.0 11.5 11.0 10.5 10.0 9.5 9.0 8.5 8.0 7.5 7.0 6.5 6.0 5.5 5.0 4.5 4.0 3.5 3.0 2.5 2.0 1.5 1.0 0.5
f1 (ppm)

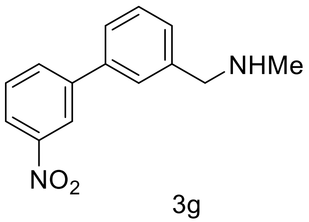
NHMe
NO2

3g

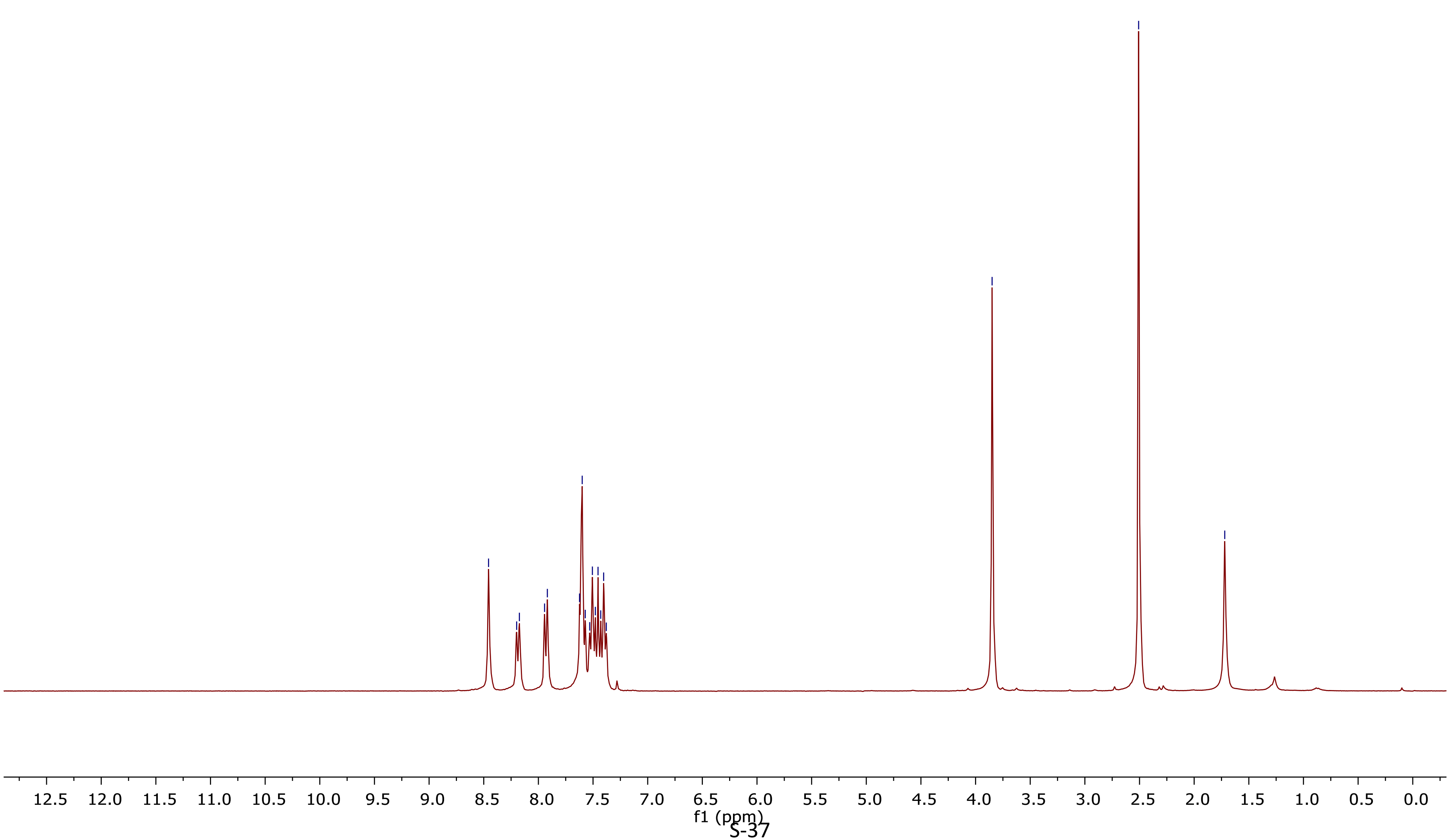
8.46
8.20
8.17
7.94
7.92
7.62
7.60
7.57
7.53
7.51
7.48
7.45
7.43
7.40
7.38
3.85
2.51
1.72
12.5
12.0
11.5
11.0
10.5
10.0
9.5
9.0
8.5
8.0
7.5
7.0
6.5
6.0
5.5
5.0
4.5
4.0
3.5
3.0
2.5
2.0
1.5
1.0
0.5
0.0
f1 (ppm)

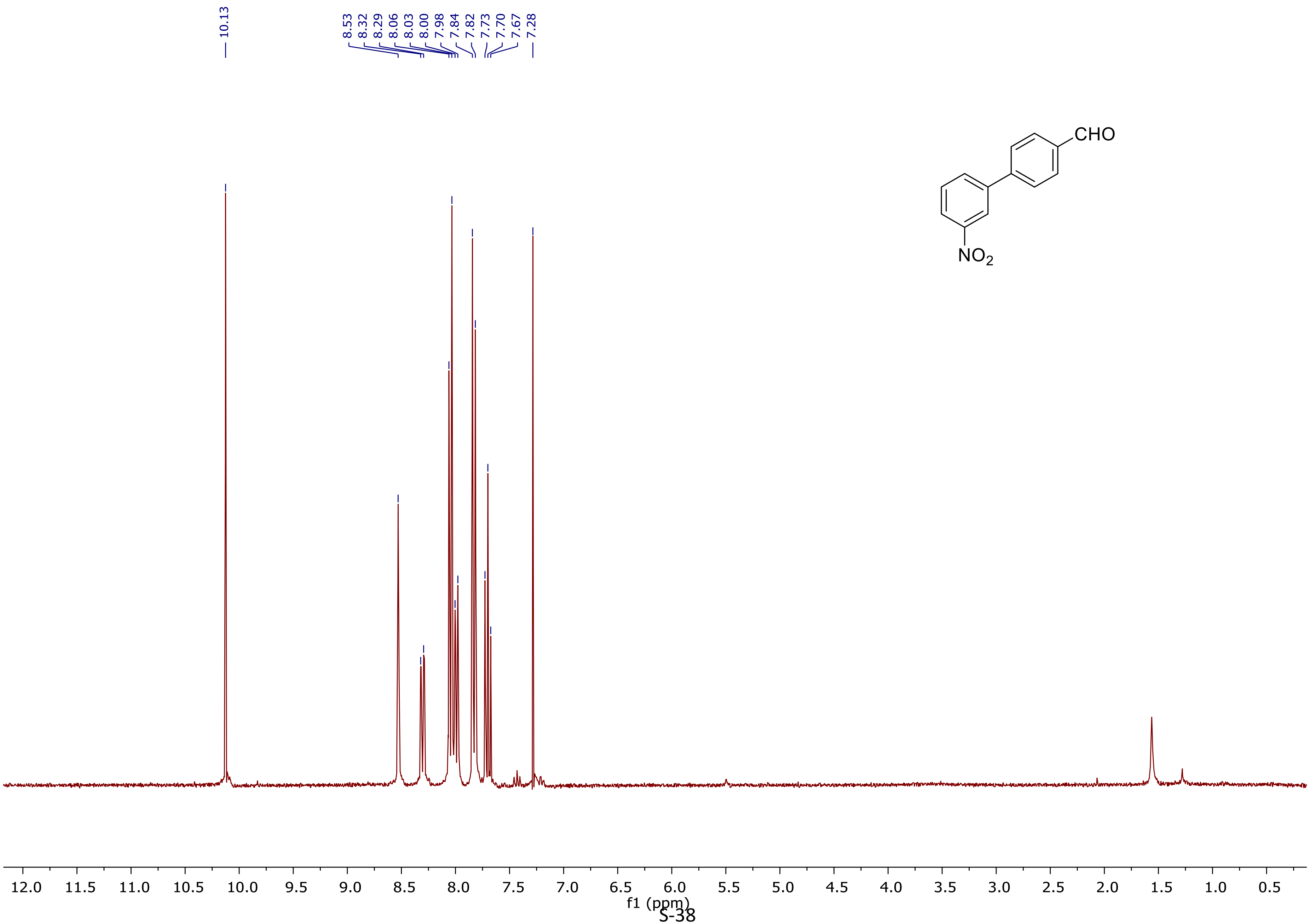
10.13
8.53
8.32
8.29
8.06
8.03
8.00
7.98
7.84
7.82
7.73
7.70
7.67
7.28
CHO
NO2
12.0
11.5
11.0
10.5
10.0
9.5
9.0
8.5
8.0
7.5
7.0
6.5
6.0
5.5
5.0
4.5
4.0
3.5
3.0
2.5
2.0
1.5
1.0
0.5
f1 (ppm)

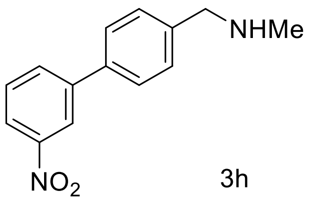

3h

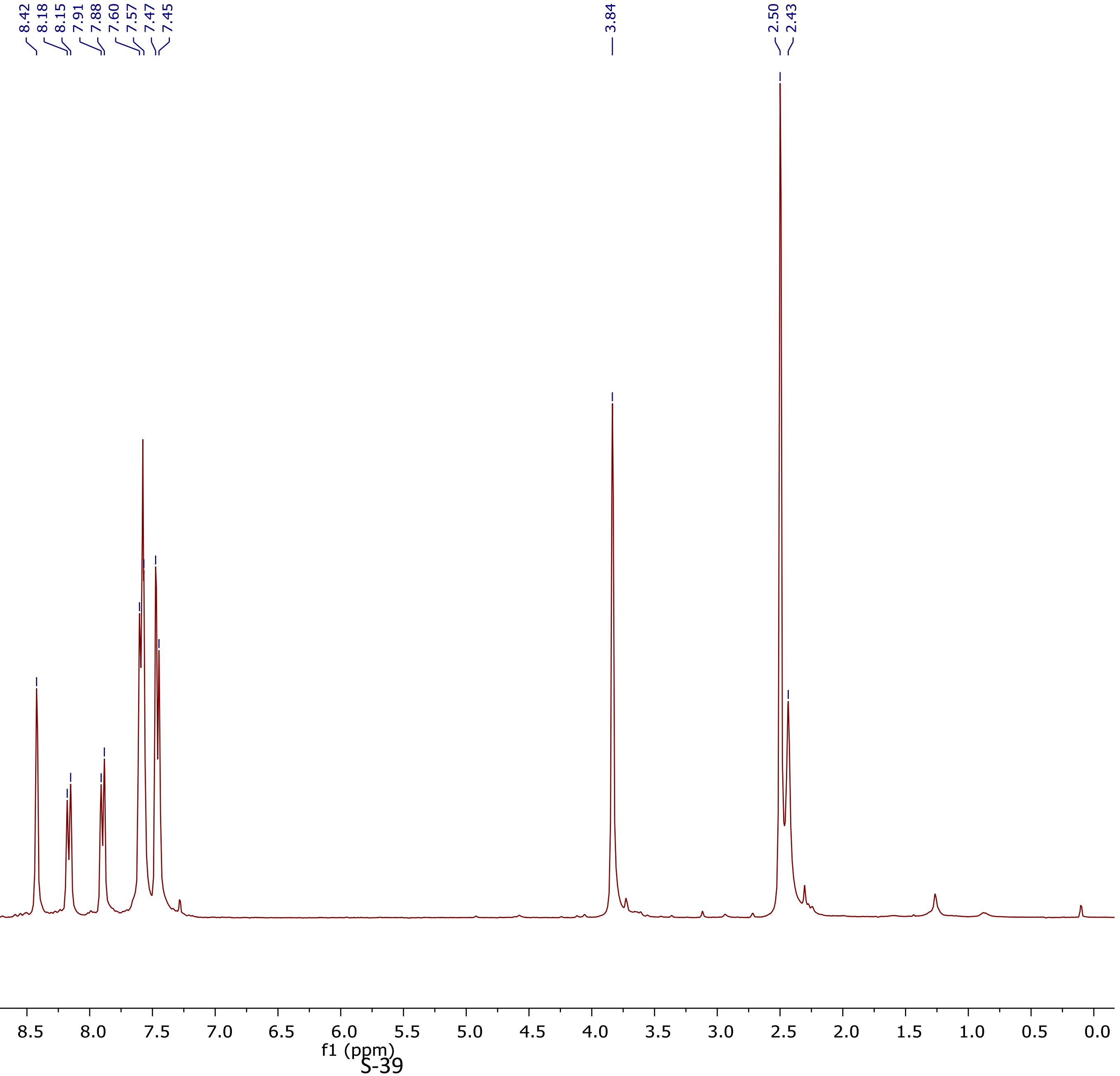
8.42
8.18
8.15
7.91
7.88
7.60
7.57
7.47
7.45
3.84
2.50
2.43
11.5 11.0 10.5 10.0 9.5 9.0 8.5 8.0 7.5 7.0 6.5 6.0 5.5 5.0 4.5 4.0 3.5 3.0 2.5 2.0 1.5 1.0 0.5 0.0
f1 (ppm)

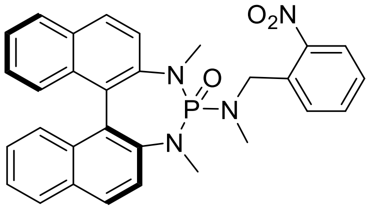

4a

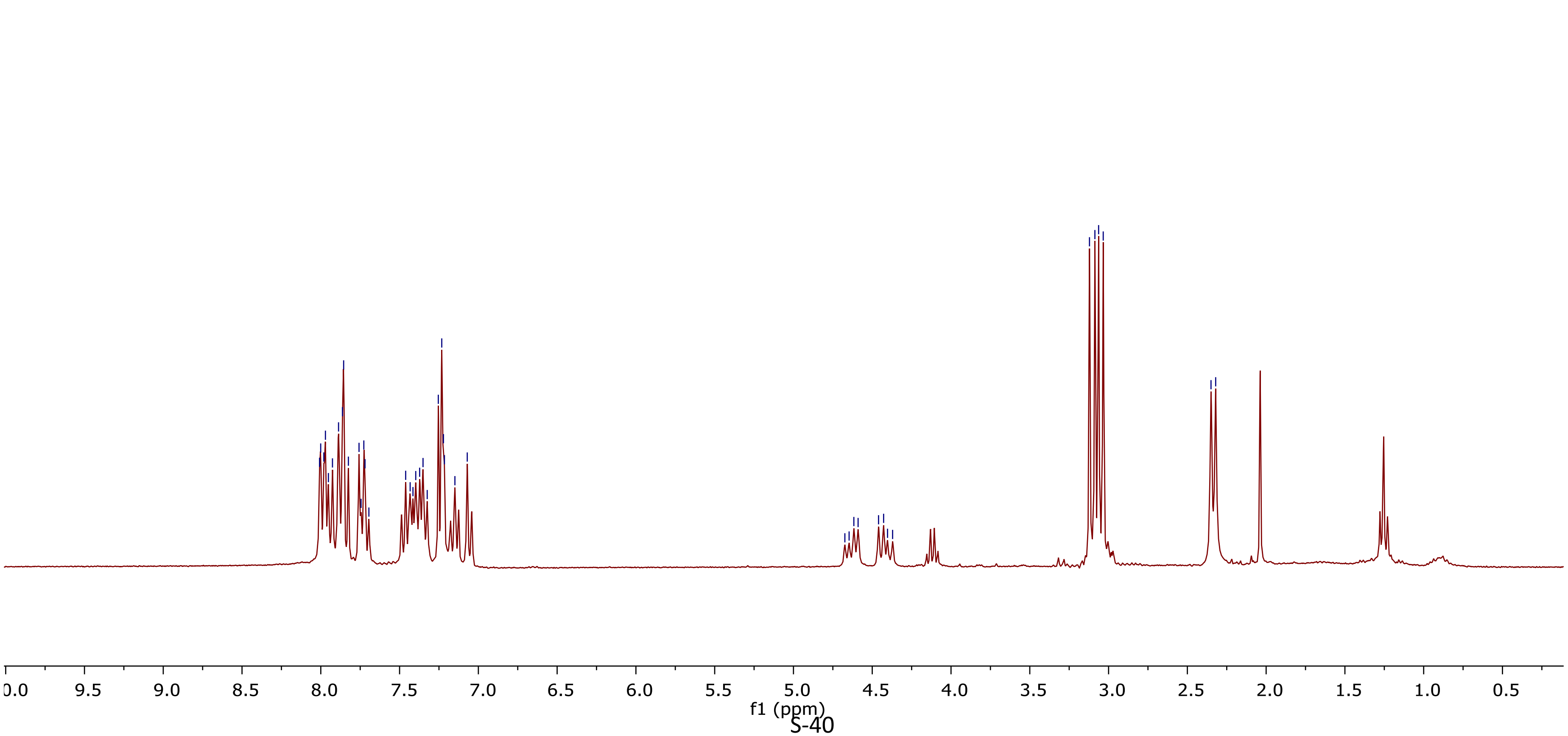
8.01 8.00 7.98 7.97 7.95 7.93 7.89 7.86 7.86 7.82 7.76 7.74 7.73 7.72 7.69 7.46 7.43 7.42 7.40 7.37 7.35 7.32 7.25 7.23 7.22 7.22 7.15 7.07
4.67 4.65 4.62 4.59 4.46 4.43 4.40 4.37
3.12 3.09 3.06 3.03
2.35 2.32
f1 (ppm)

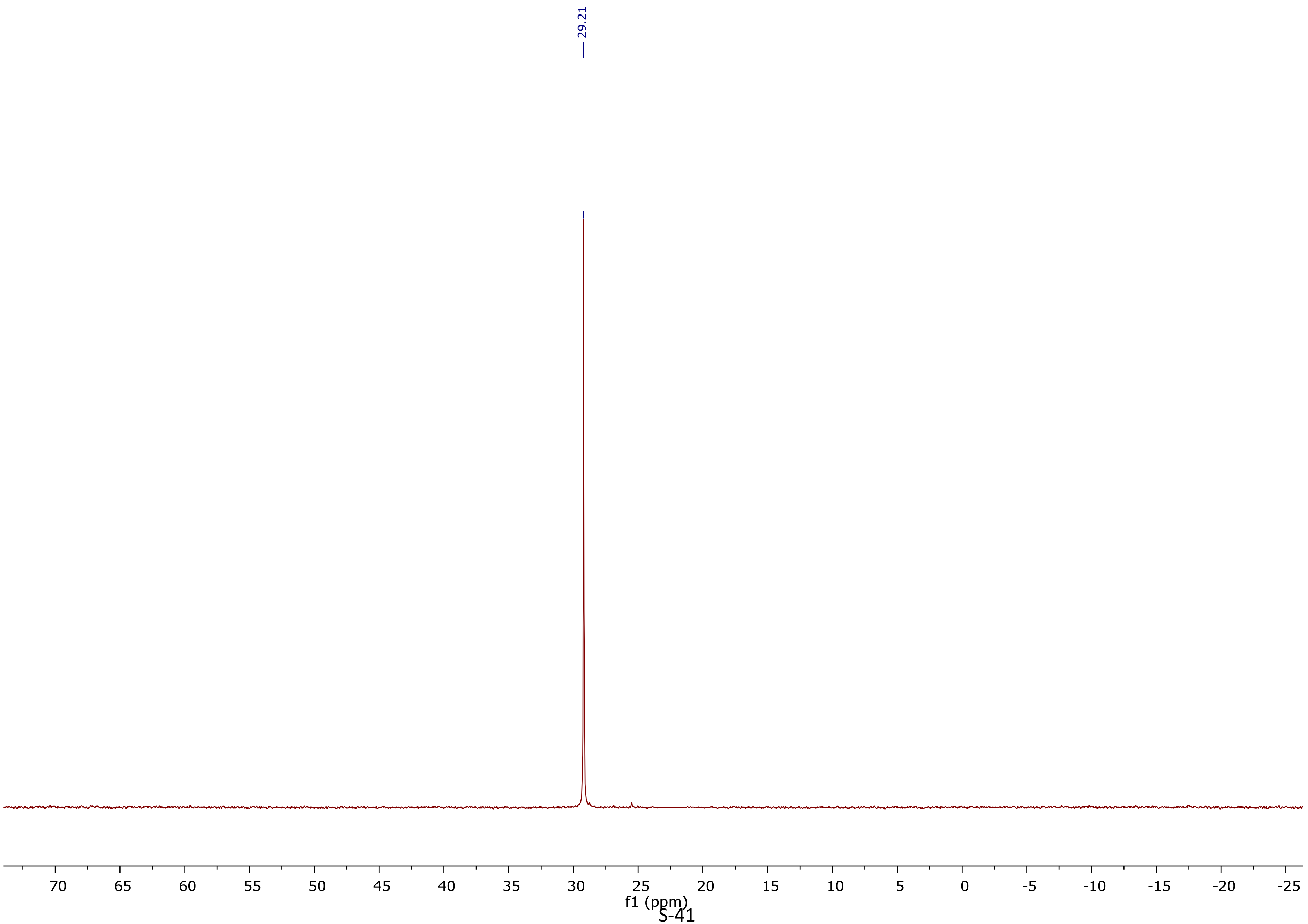
29.21
70 65 60 55 50 45 40 35 30 25 20 15 10 5 0 -5 -10 -15 -20 -25
f1 (ppm)

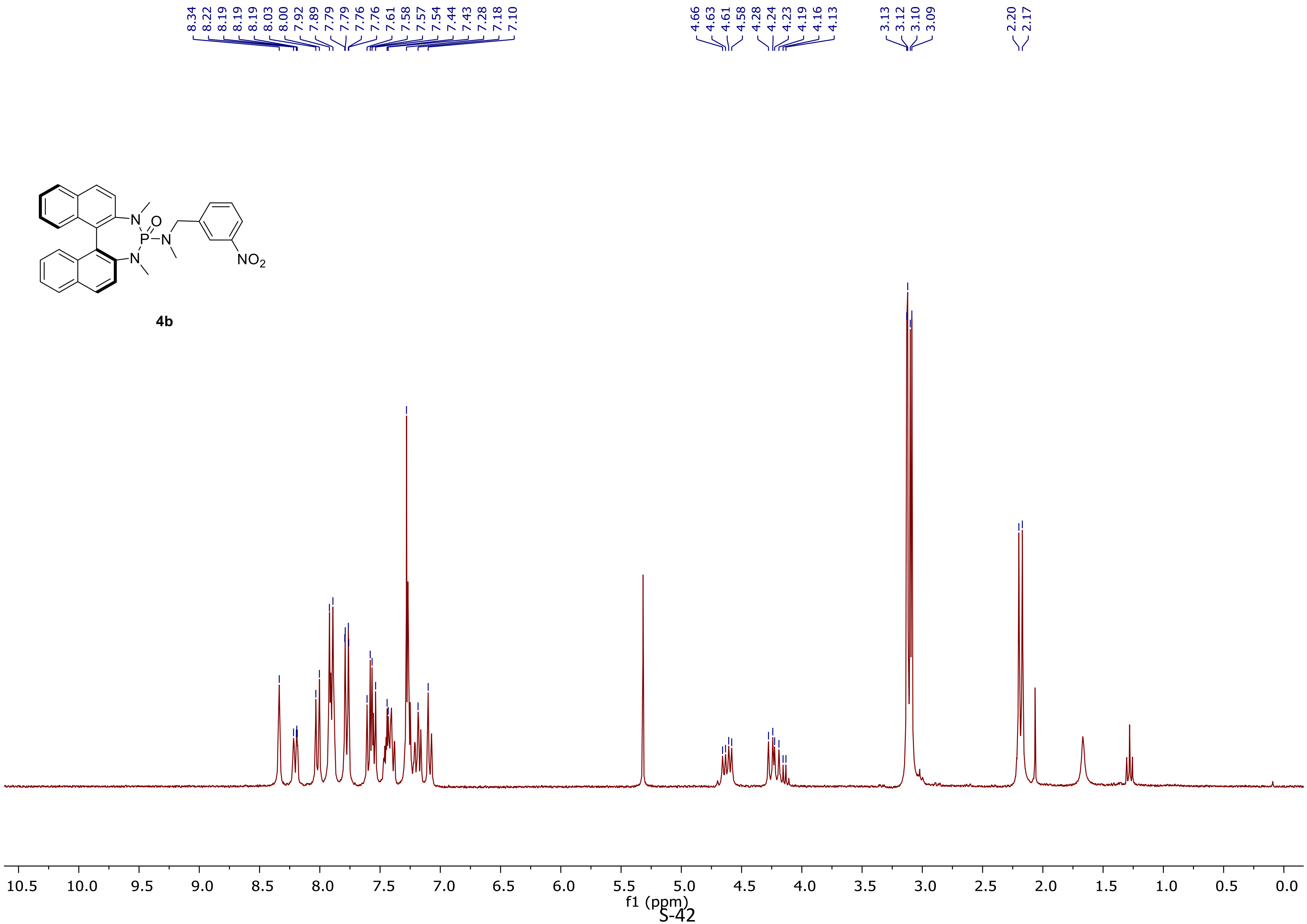

8.34 8.22 8.19 8.19 8.19 8.03 8.00 7.92 7.89 7.79 7.79 7.76 7.76 7.61 7.58 7.57 7.54 7.44 7.43 7.28 7.18 7.10
4.66 4.63 4.61 4.58 4.28 4.24 4.23 4.19 4.16 4.13
3.13 3.12 3.10 3.09
2.20 2.17
10.5 10.0 9.5 9.0 8.5 8.0 7.5 7.0 6.5 6.0 5.5 5.0 4.5 4.0 3.5 3.0 2.5 2.0 1.5 1.0 0.5 0.0
f1 (ppm)

4b

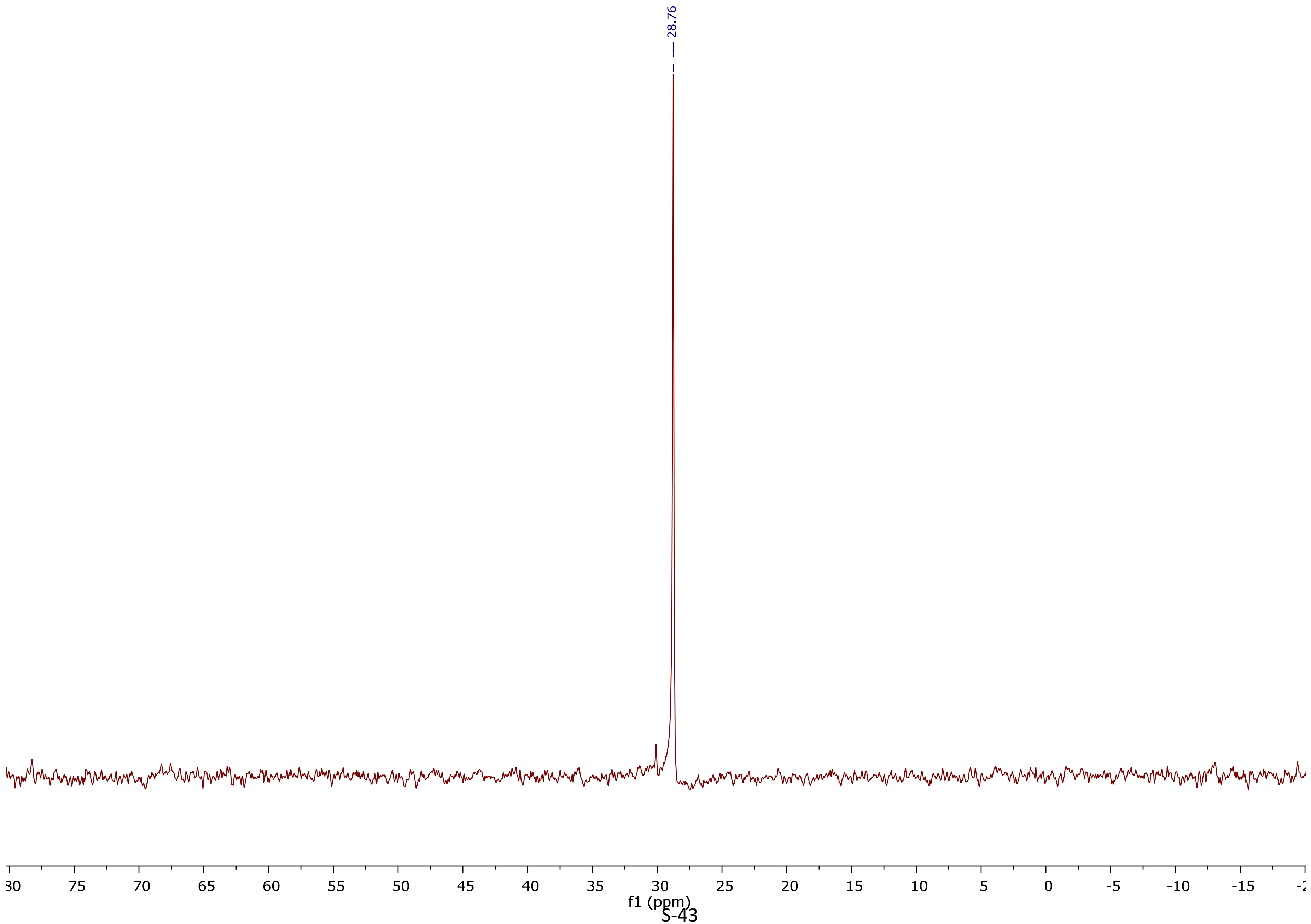

28.76
f1 (ppm)

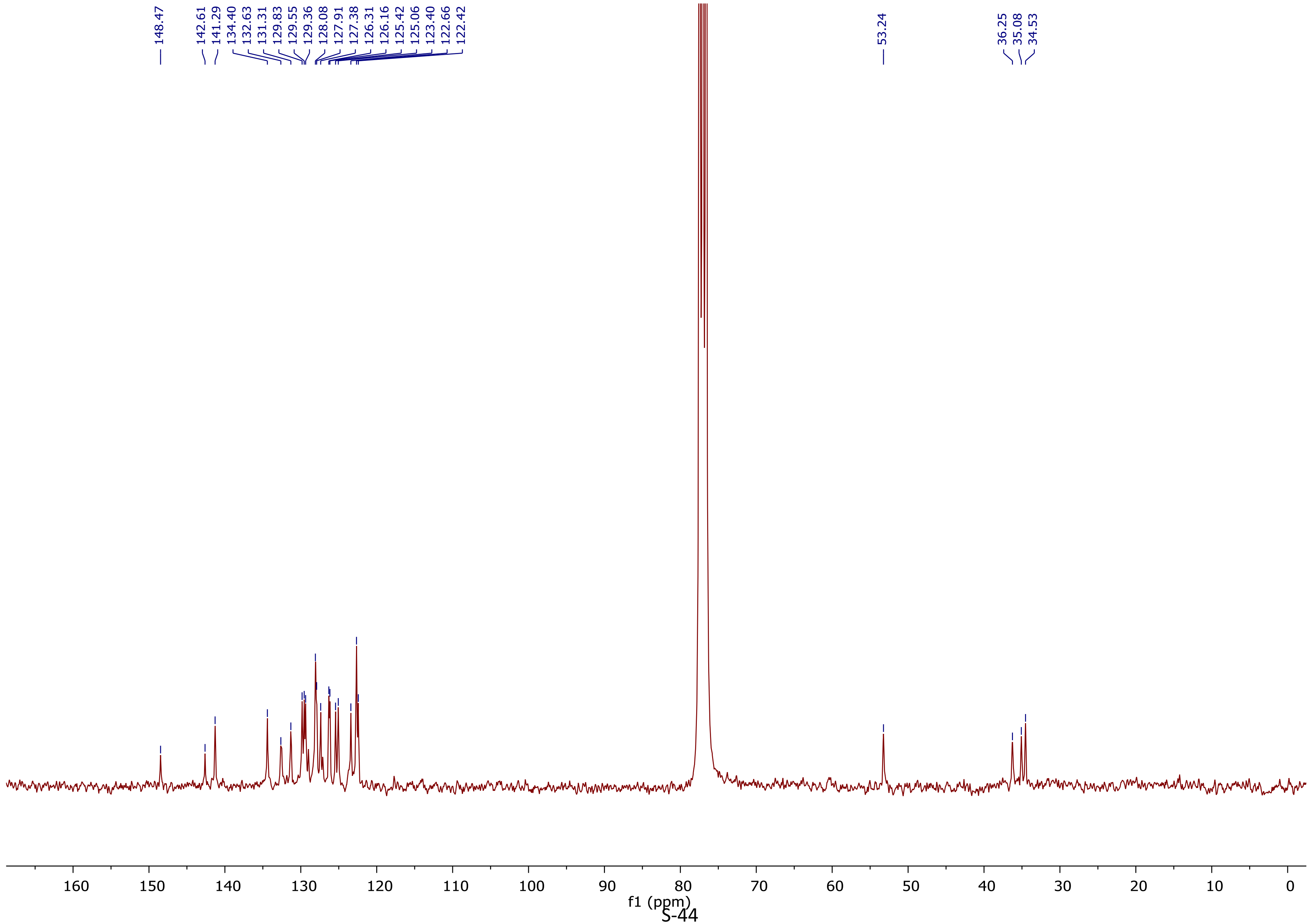
148.47
142.61
141.29
134.40
132.63
131.31
129.83
129.55
129.36
128.08
127.91
127.38
126.31
126.16
125.42
125.06
123.40
122.66
122.42
53.24
36.25
35.08
34.53
160
150
140
130
120
110
100
90
80
70
60
50
40
30
20
10
0
f1 (ppm)

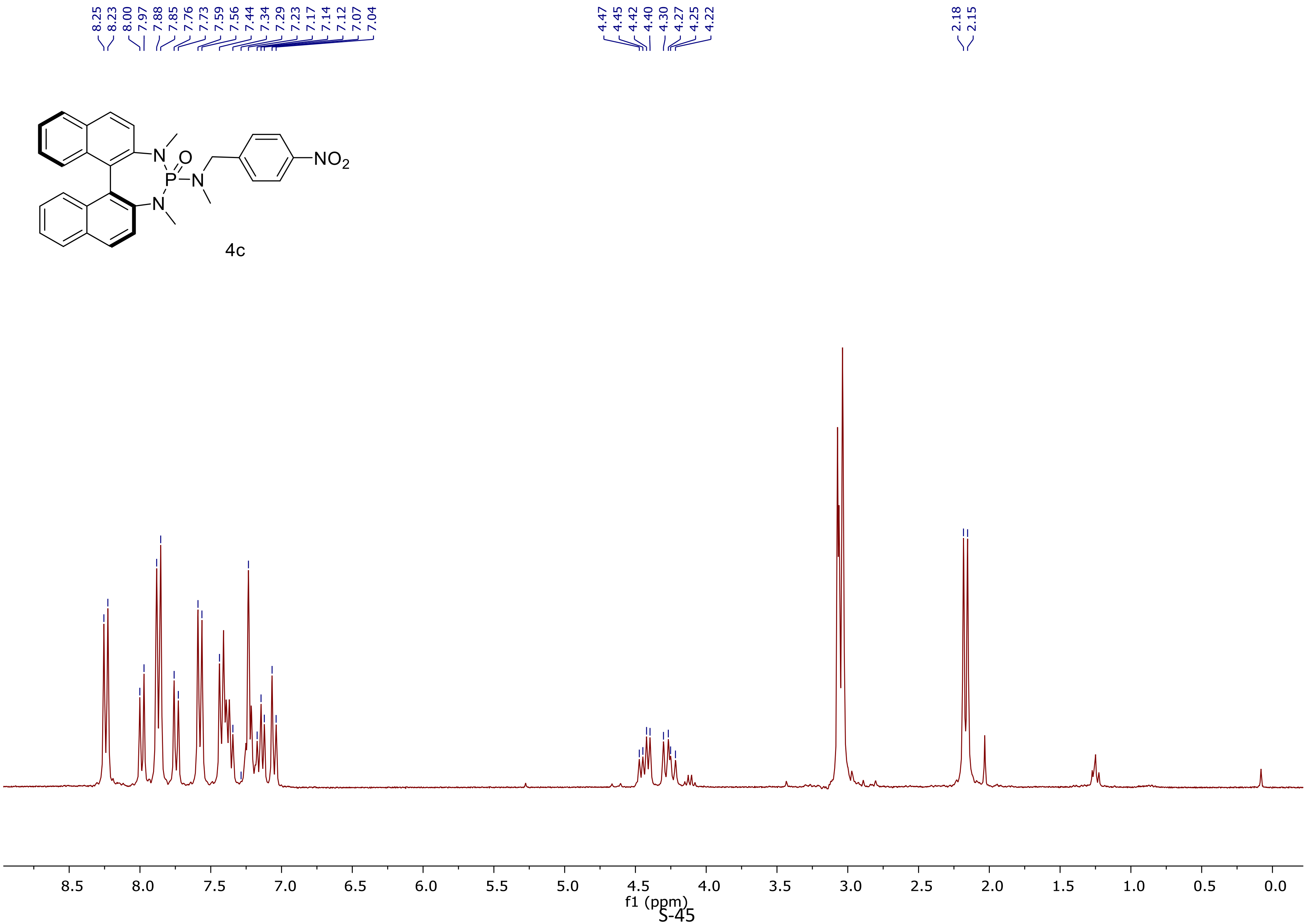
8.25
8.23
8.00
7.97
7.88
7.85
7.76
7.73
7.59
7.56
7.44
7.34
7.29
7.23
7.17
7.14
7.12
7.07
7.04
4.47
4.45
4.42
4.40
4.30
4.27
4.25
4.22
2.18
2.15
4c
f1 (ppm)

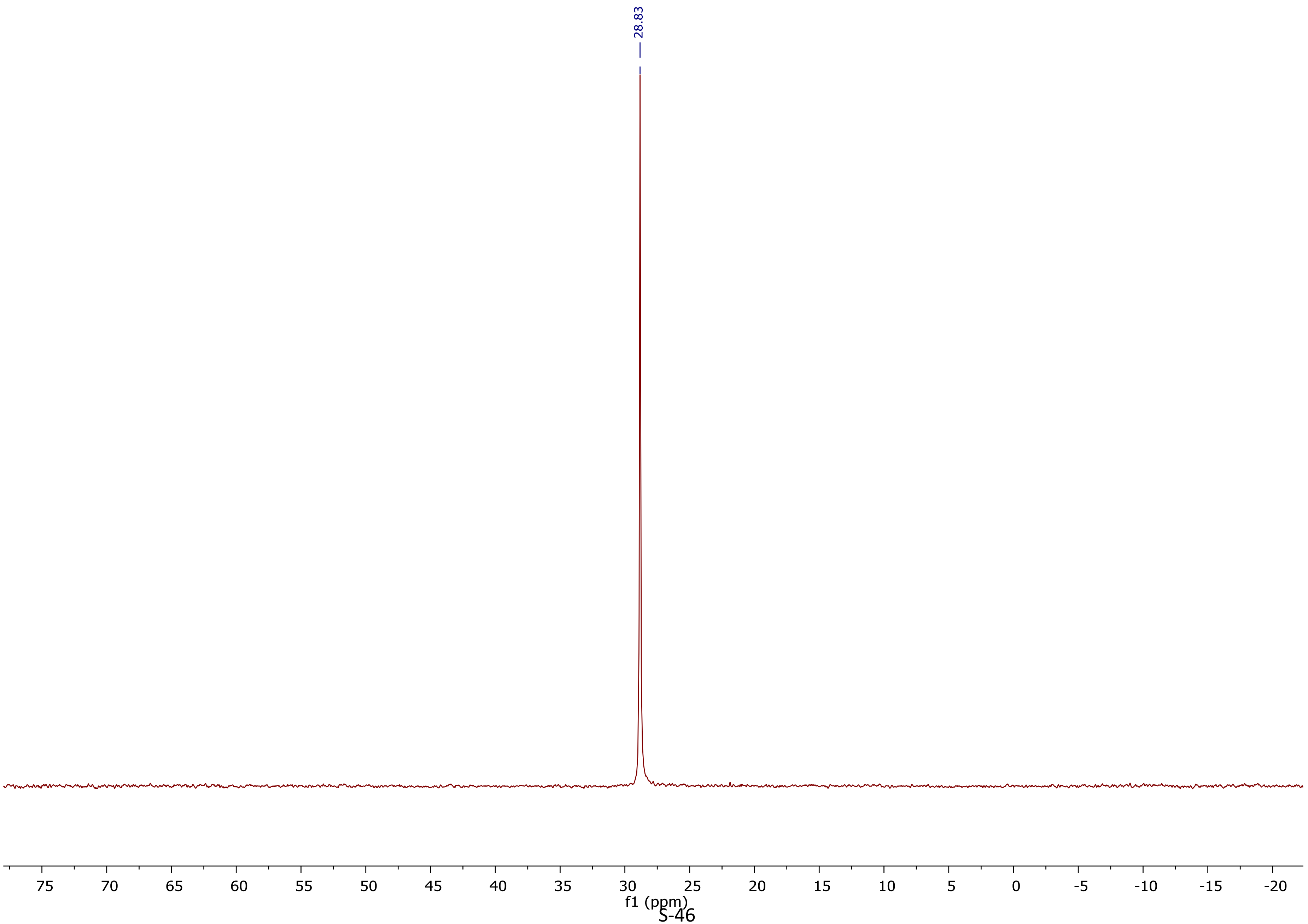
28.83
75
70
65
60
55
50
45
40
35
30
25
20
15
10
5
0
-5
-10
-15
-20
f1 (ppm)

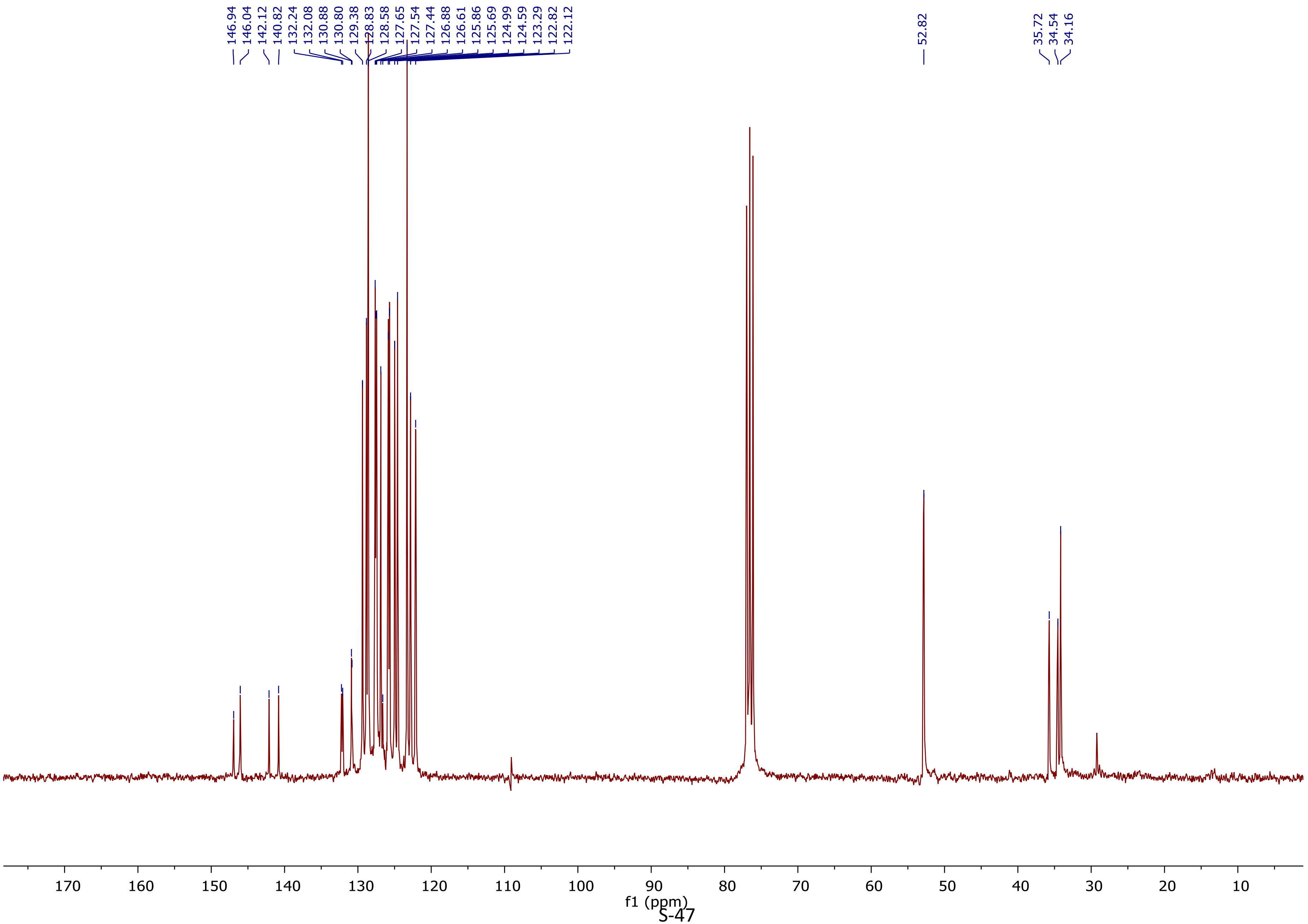
146.94
146.04
142.12
140.82
132.24
132.08
130.88
130.80
129.38
128.83
128.58
127.65
127.54
127.44
126.88
126.61
125.86
125.69
124.99
124.59
123.29
122.82
122.12
52.82
35.72
34.54
34.16
170
160
150
140
130
120
110
100
90
80
70
60
50
40
30
20
10
f1 (ppm)

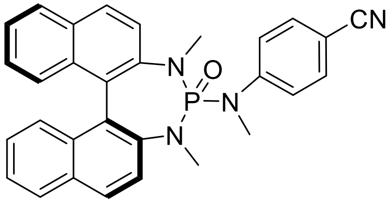
CN
N
O
P
N
N

4e

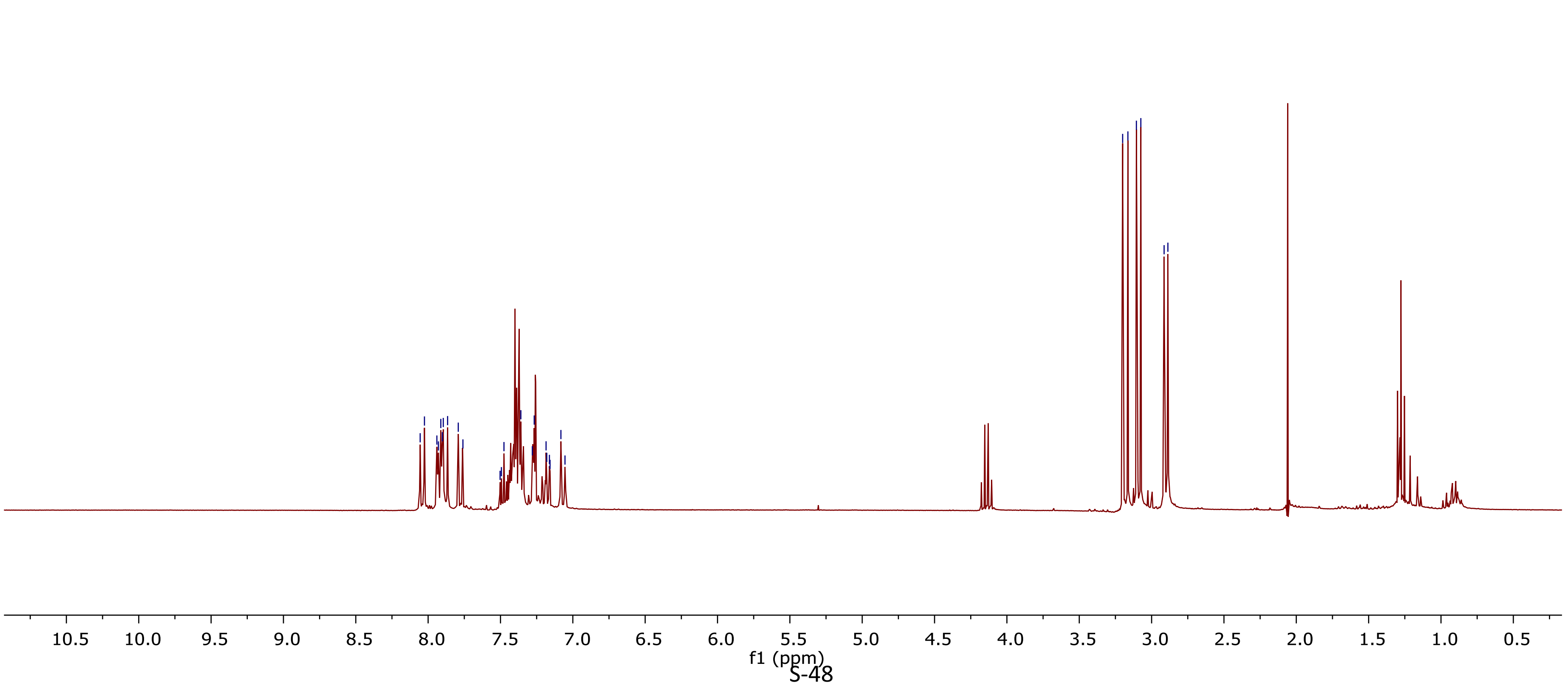
8.05
8.03
7.94
7.93
7.91
7.90
7.90
7.87
7.79
7.76
7.50
7.49
7.48
7.36
7.28
7.27
7.18
7.18
7.16
7.08
7.05
3.20
3.16
3.11
3.07
2.91
2.89
10.5
10.0
9.5
9.0
8.5
8.0
7.5
7.0
6.5
6.0
5.5
5.0
4.5
4.0
3.5
3.0
2.5
2.0
1.5
1.0
0.5
f1 (ppm)

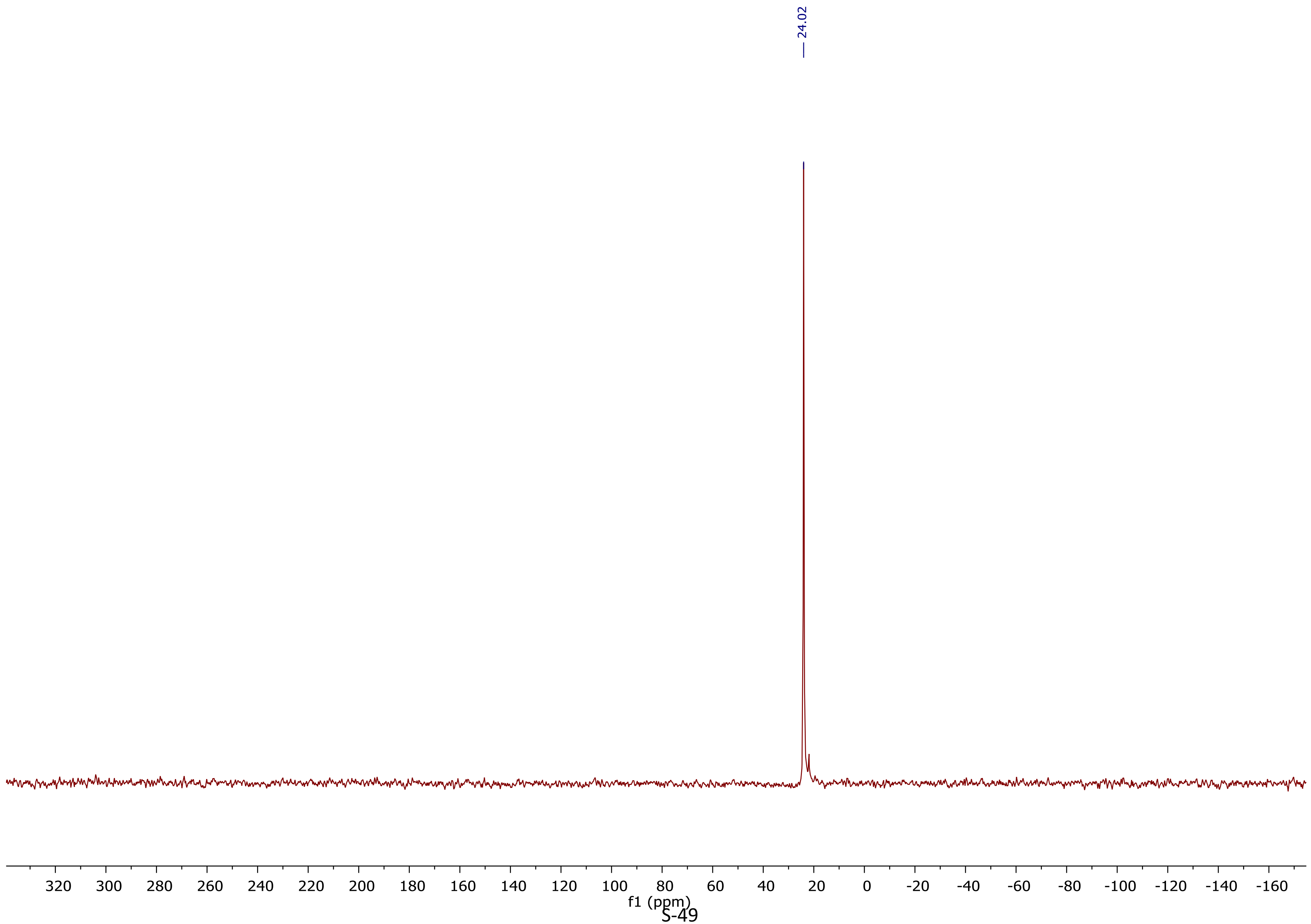
24.02
320 300 280 260 240 220 200 180 160 140 120 100 80 60 40 20 0 -20 -40 -60 -80 -100 -120 -140 -160
f1 (ppm)

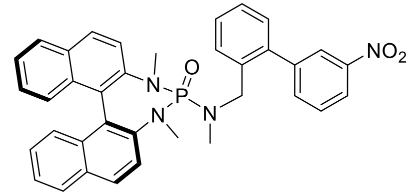
NO2
O
P
N
N
N

4f

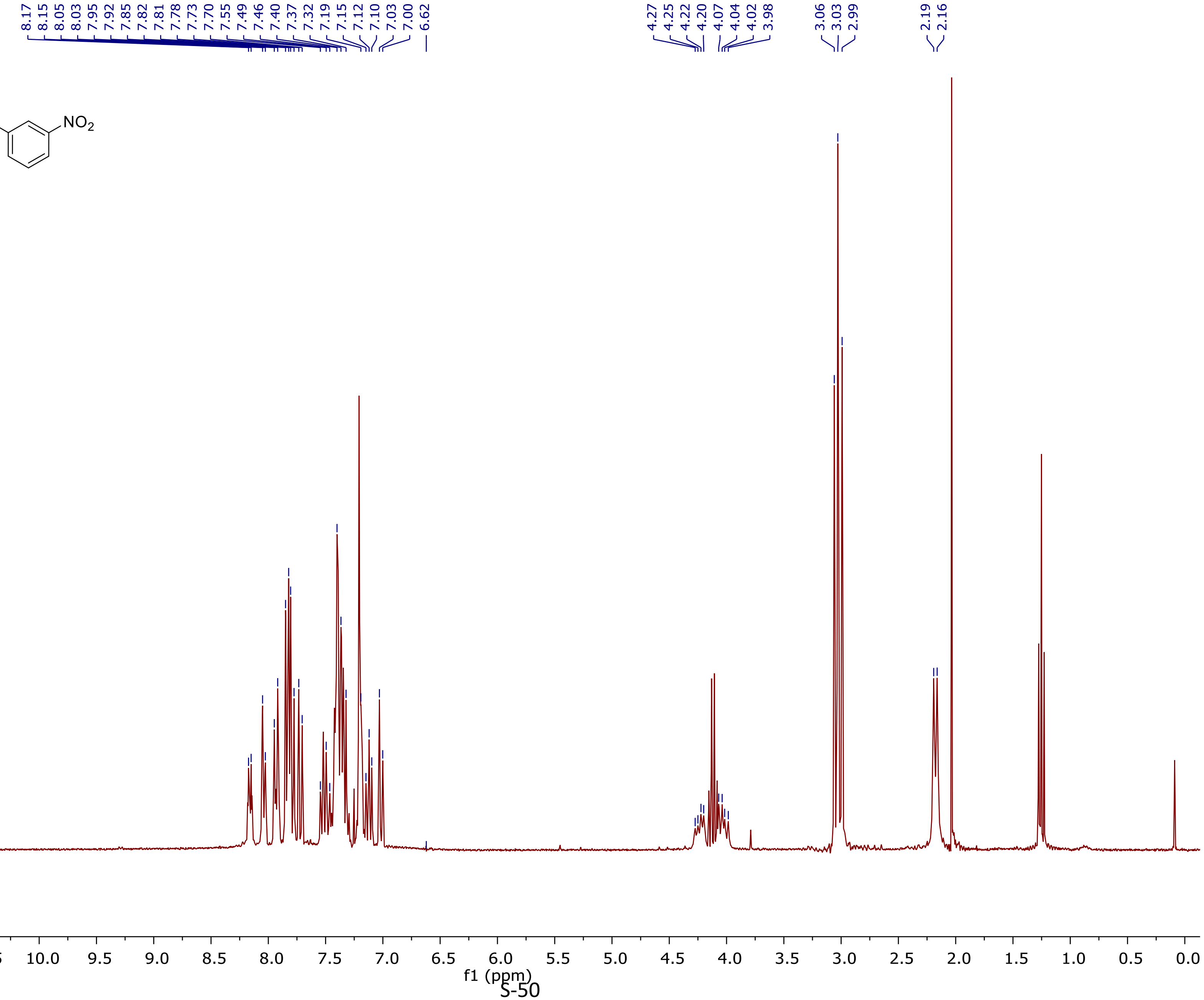
8.17
8.15
8.05
8.03
7.95
7.92
7.85
7.82
7.81
7.78
7.73
7.70
7.55
7.49
7.46
7.40
7.37
7.32
7.19
7.15
7.12
7.10
7.03
7.00
6.62
4.27
4.25
4.22
4.20
4.07
4.04
4.02
3.98
3.06
3.03
2.99
2.19
2.16
12.0 11.5 11.0 10.5 10.0 9.5 9.0 8.5 8.0 7.5 7.0 6.5 6.0 5.5 5.0 4.5 4.0 3.5 3.0 2.5 2.0 1.5 1.0 0.5 0.0
f1 (ppm)

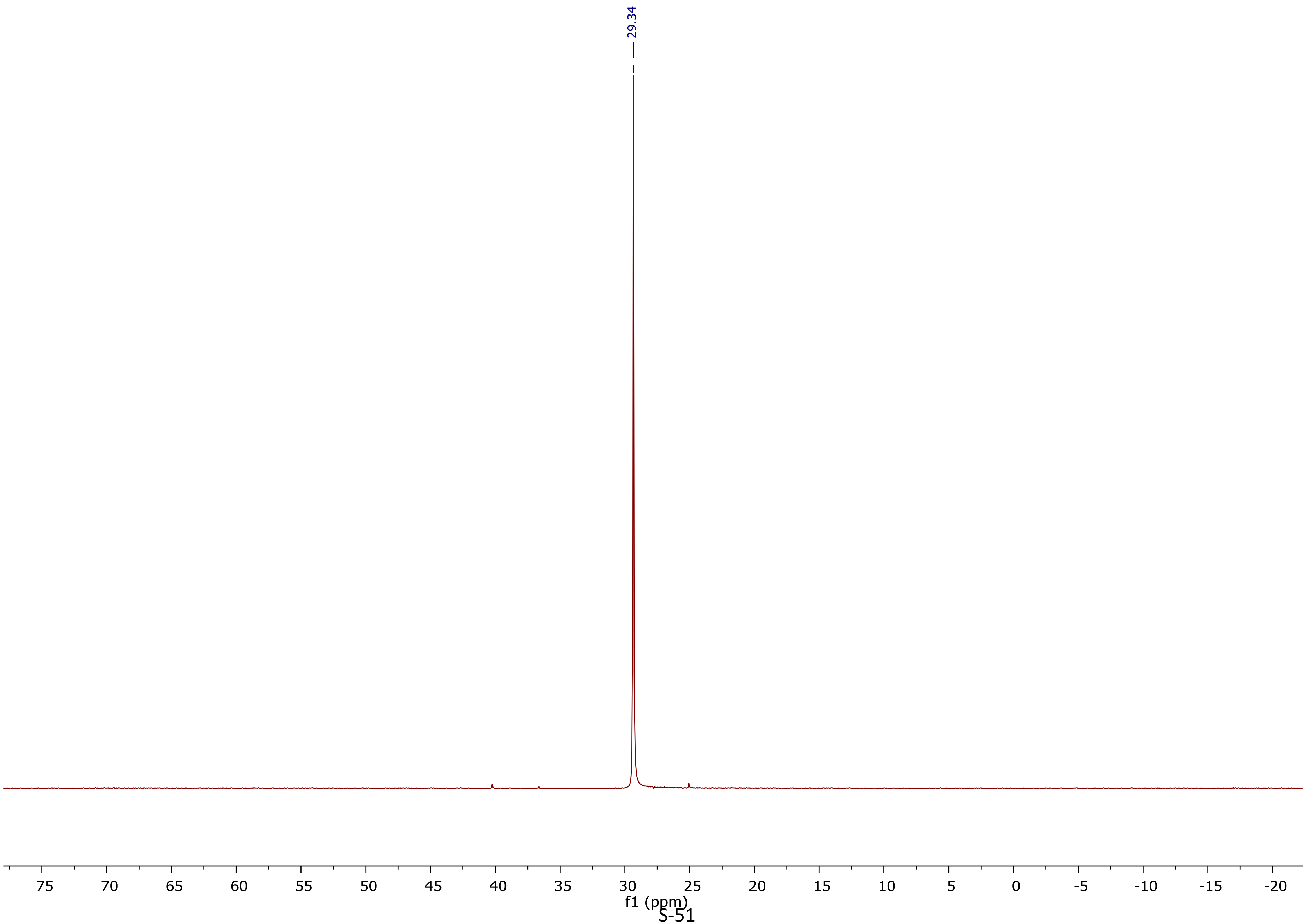
29.34
75 70 65 60 55 50 45 40 35 30 25 20 15 10 5 0 -5 -10 -15 -20
f1 (ppm)

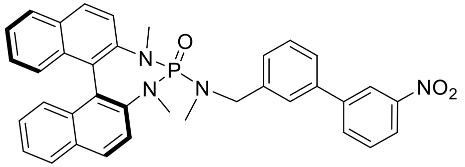

4g

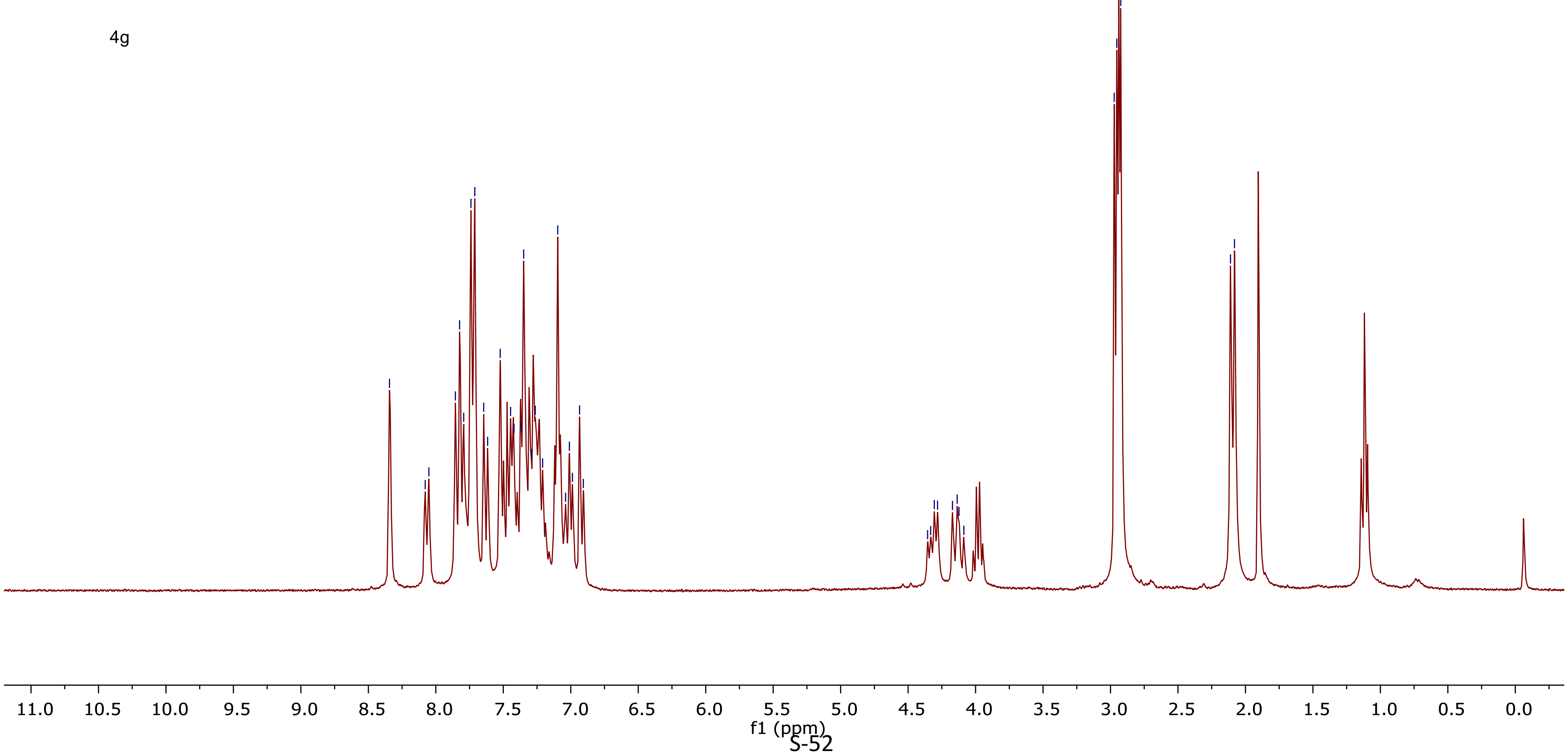

8.34
8.08
8.05
7.86
7.82
7.79
7.74
7.71
7.65
7.62
7.52
7.45
7.42
7.37
7.35
7.29
7.26
7.21
7.10
7.04
7.01
6.99
6.93
6.91
4.36
4.33
4.31
4.28
4.17
4.14
4.12
4.09
2.97
2.95
2.94
2.92
2.11
2.08
11.0 10.5 10.0 9.5 9.0 8.5 8.0 7.5 7.0 6.5 6.0 5.5 5.0 4.5 4.0 3.5 3.0 2.5 2.0 1.5 1.0 0.5 0.0
f1 (ppm)

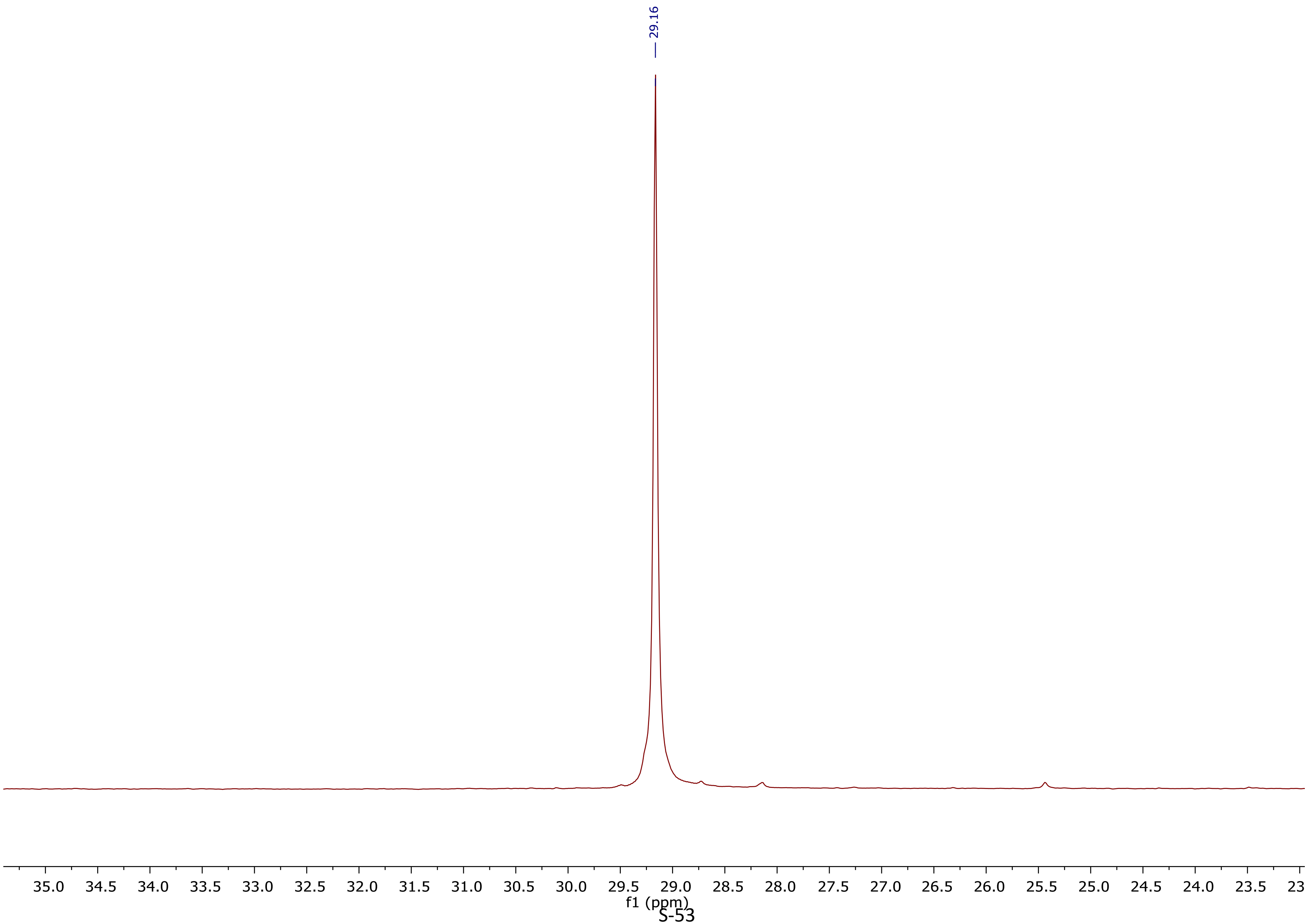
29.16
35.0
34.5
34.0
33.5
33.0
32.5
32.0
31.5
31.0
30.5
30.0
29.5
29.0
28.5
28.0
27.5
27.0
26.5
26.0
25.5
25.0
24.5
24.0
23.5
23
f1 (ppm)

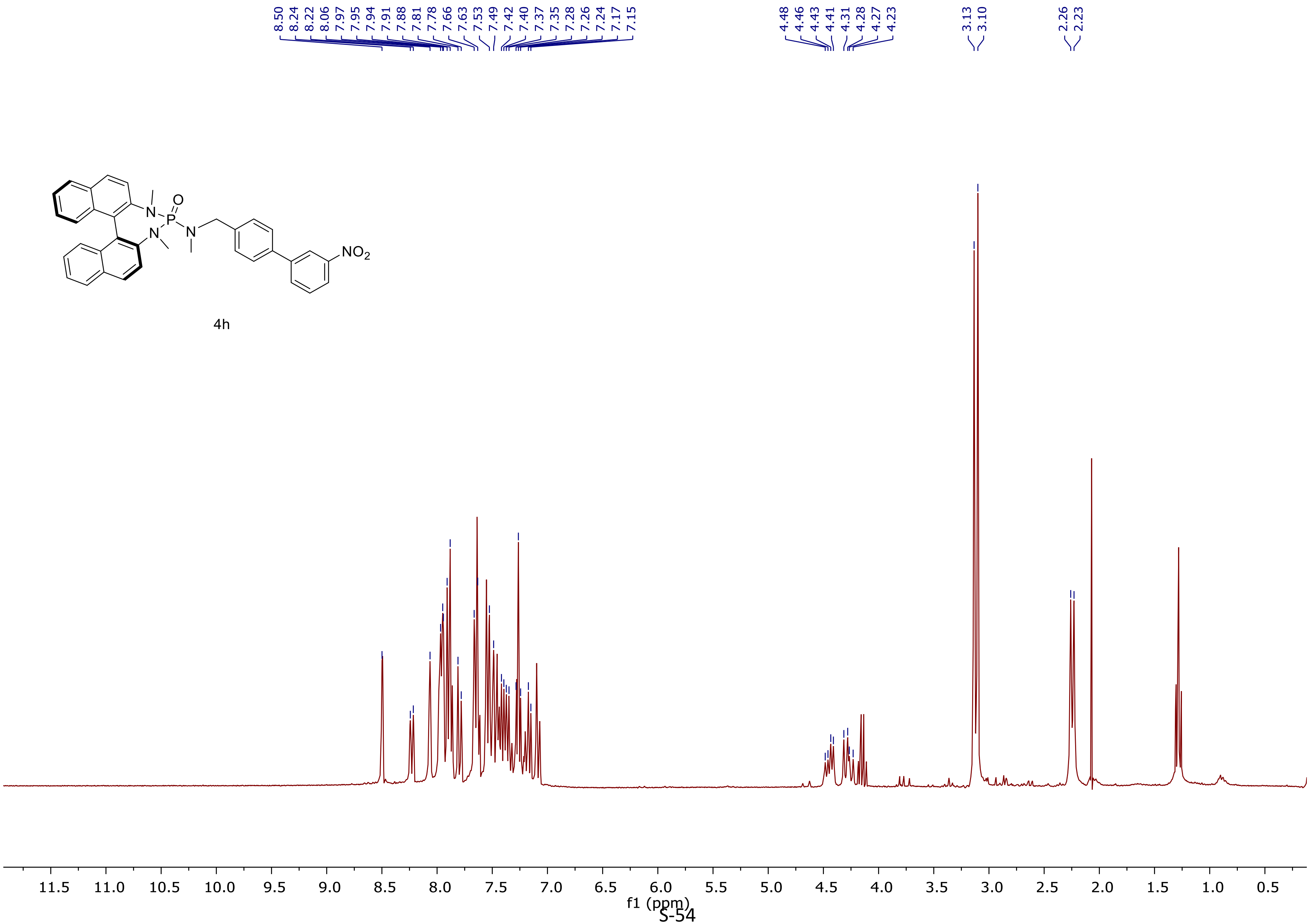

8.50 8.24 8.22 8.06 7.97 7.95 7.94 7.91 7.88 7.81 7.78 7.66 7.63 7.53 7.49 7.42 7.40 7.37 7.35 7.28 7.26 7.24 7.17 7.15
4.48 4.46 4.43 4.41 4.31 4.28 4.27 4.23
3.13 3.10
2.26 2.23
NO2
4h
11.5 11.0 10.5 10.0 9.5 9.0 8.5 8.0 7.5 7.0 6.5 6.0 5.5 5.0 4.5 4.0 3.5 3.0 2.5 2.0 1.5 1.0 0.5
f1 (ppm)

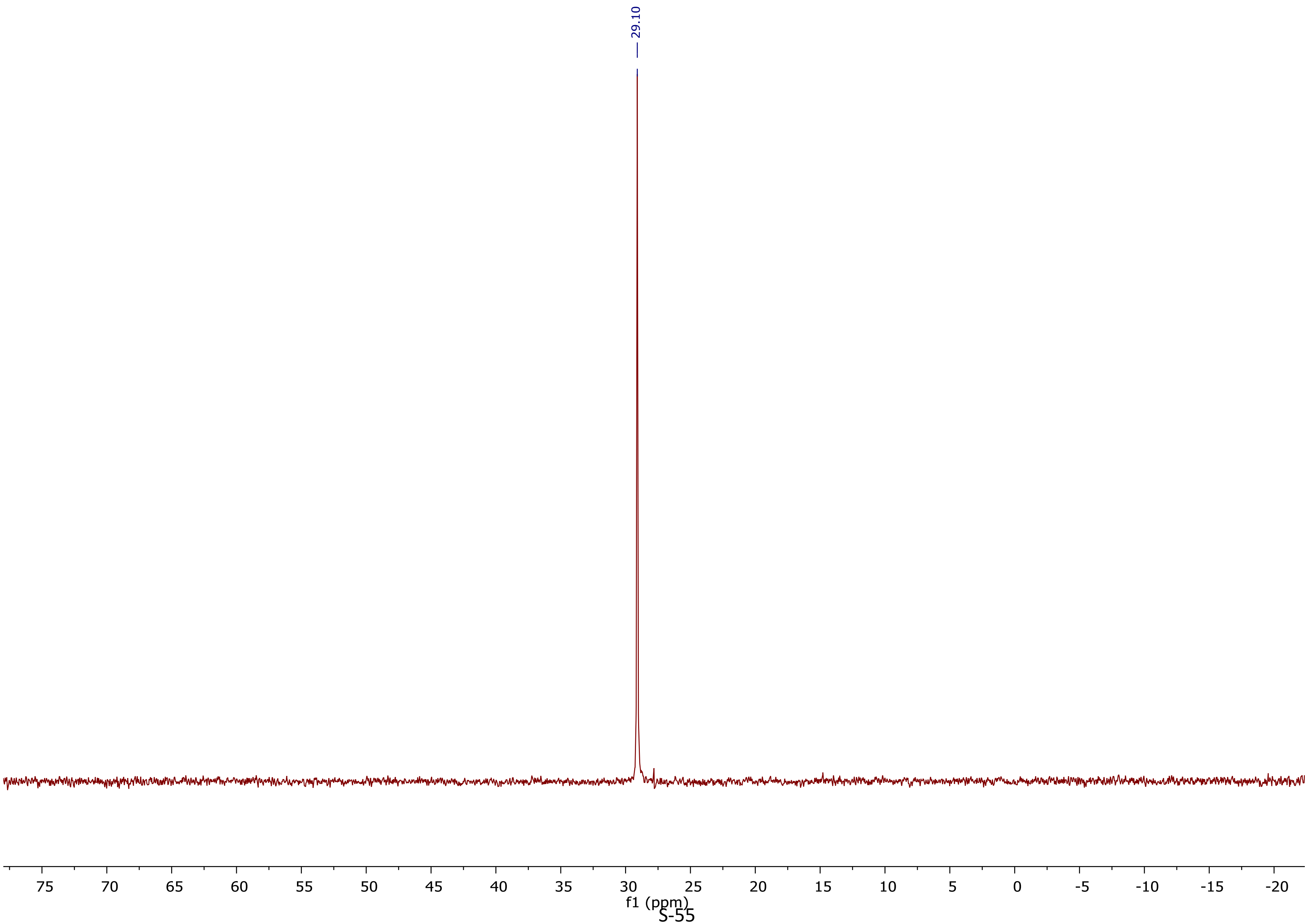

29.10
75
70
65
60
55
50
45
40
35
30
25
20
15
10
5
0
-5
-10
-15
-20
f1 (ppm)

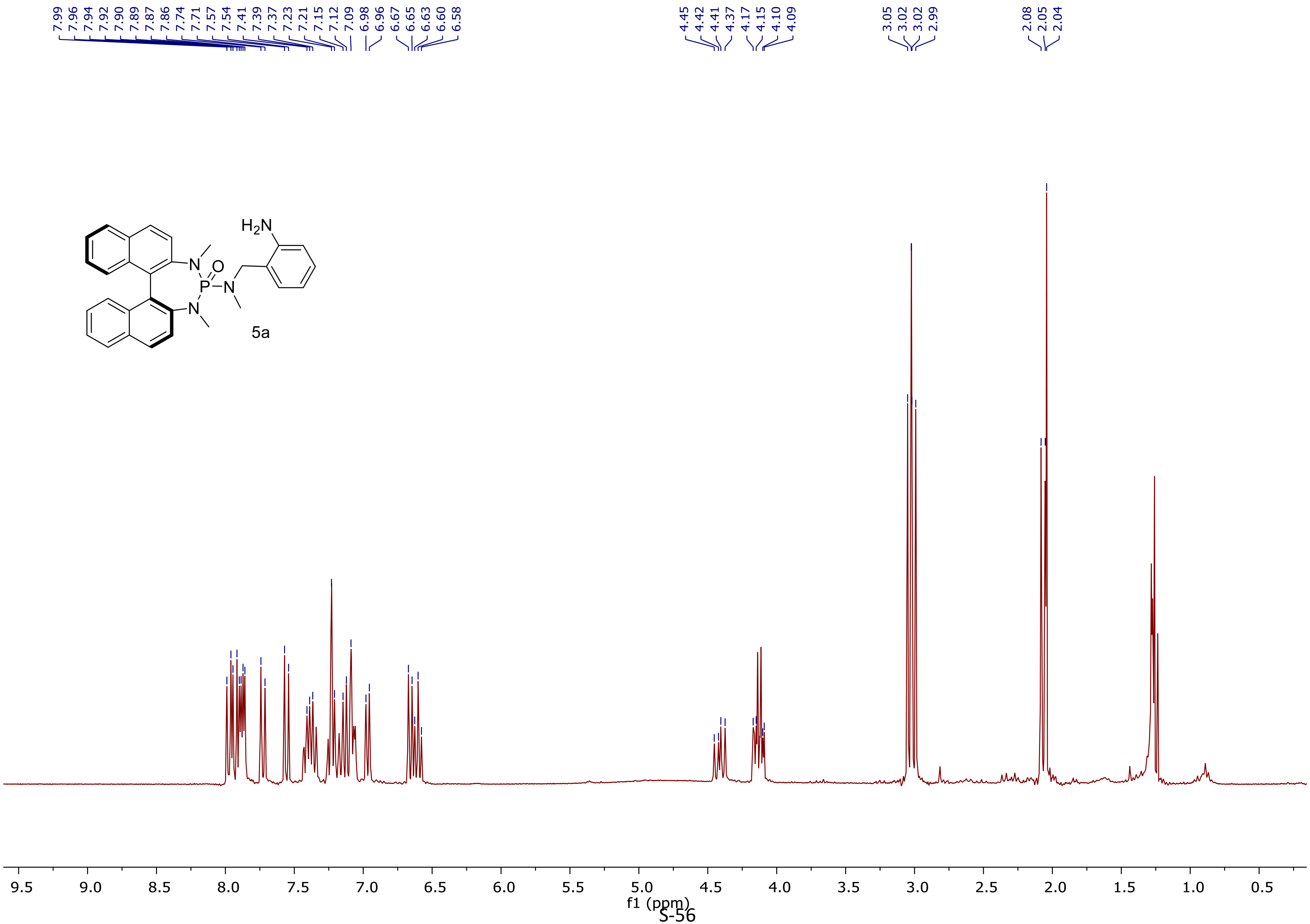
7.99
7.96
7.94
7.92
7.90
7.89
7.87
7.86
7.74
7.71
7.57
7.54
7.41
7.39
7.37
7.23
7.21
7.15
7.12
7.09
6.98
6.96
6.67
6.65
6.63
6.60
6.58
4.45
4.42
4.41
4.37
4.17
4.15
4.10
4.09
3.05
3.02
3.02
2.99
2.08
2.05
2.04
H2N
N
O
P
N
N
5a
9.5
9.0
8.5
8.0
7.5
7.0
6.5
6.0
5.5
5.0
4.5
4.0
3.5
3.0
2.5
2.0
1.5
1.0
0.5
f1 (ppm)

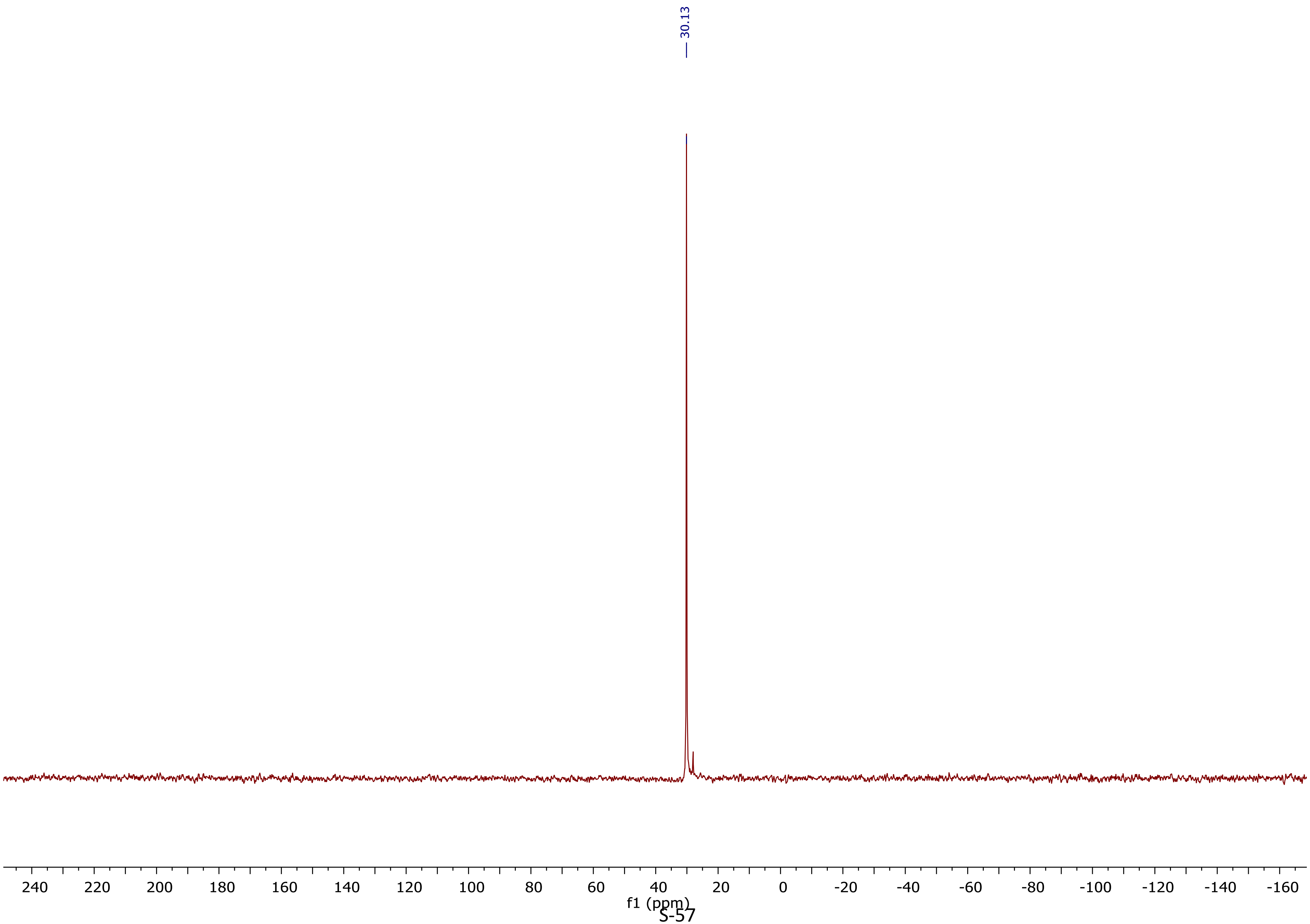

30.13
240
220
200
180
160
140
120
100
80
60
40
20
0
-20
-40
-60
-80
-100
-120
-140
-160
f1 (ppm)

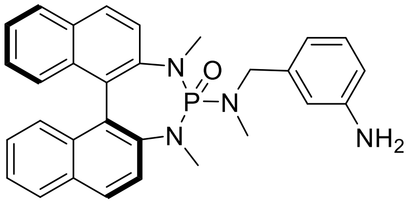
N
O
P
N
N
NH2

5b

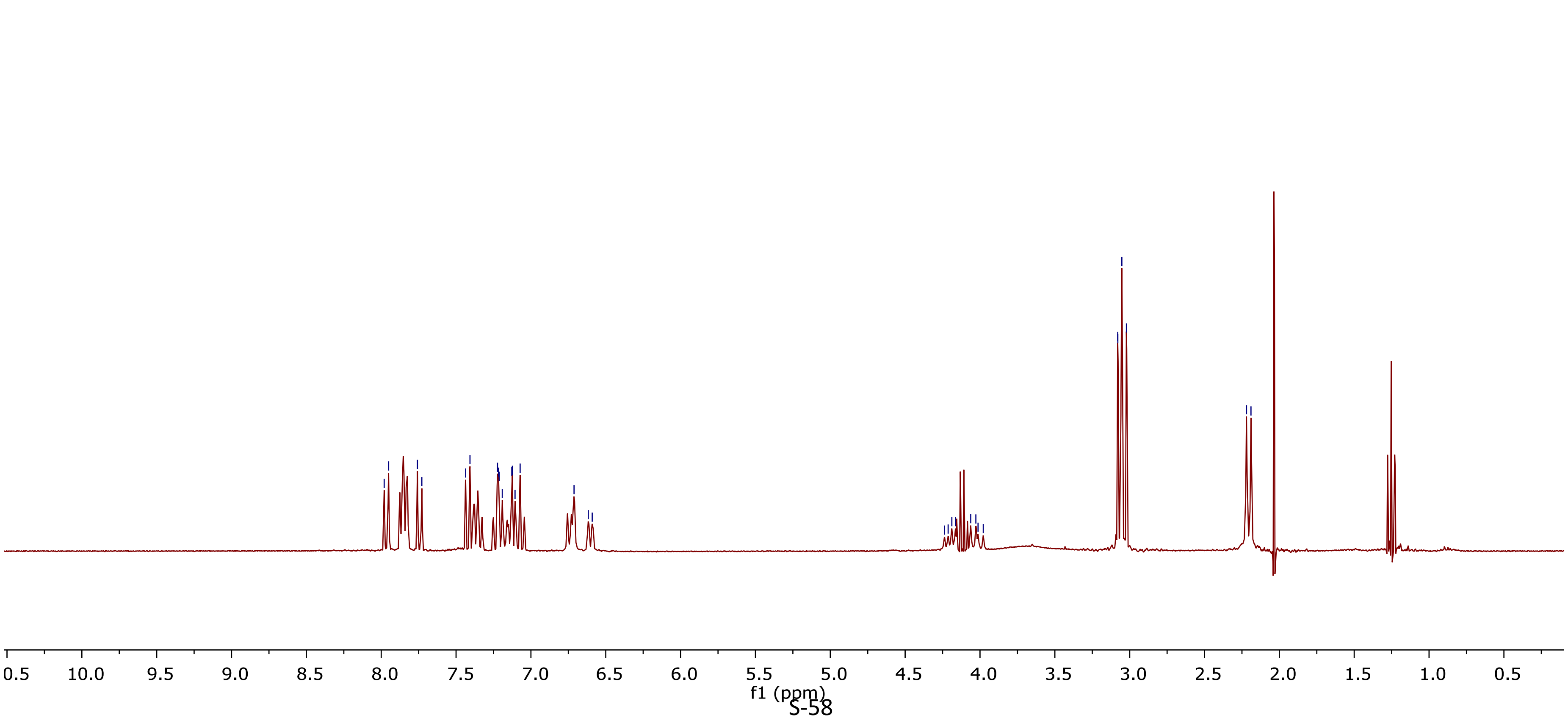
7.98
7.95
7.76
7.73
7.44
7.41
7.22
7.22
7.21
7.19
7.13
7.12
7.11
7.07
6.71
6.62
6.59
4.24
4.21
4.19
4.16
4.06
4.03
4.01
3.98
3.08
3.08
3.05
3.02
2.22
2.19
0.5 10.0 9.5 9.0 8.5 8.0 7.5 7.0 6.5 6.0 5.5 5.0 4.5 4.0 3.5 3.0 2.5 2.0 1.5 1.0 0.5
f1 (ppm)

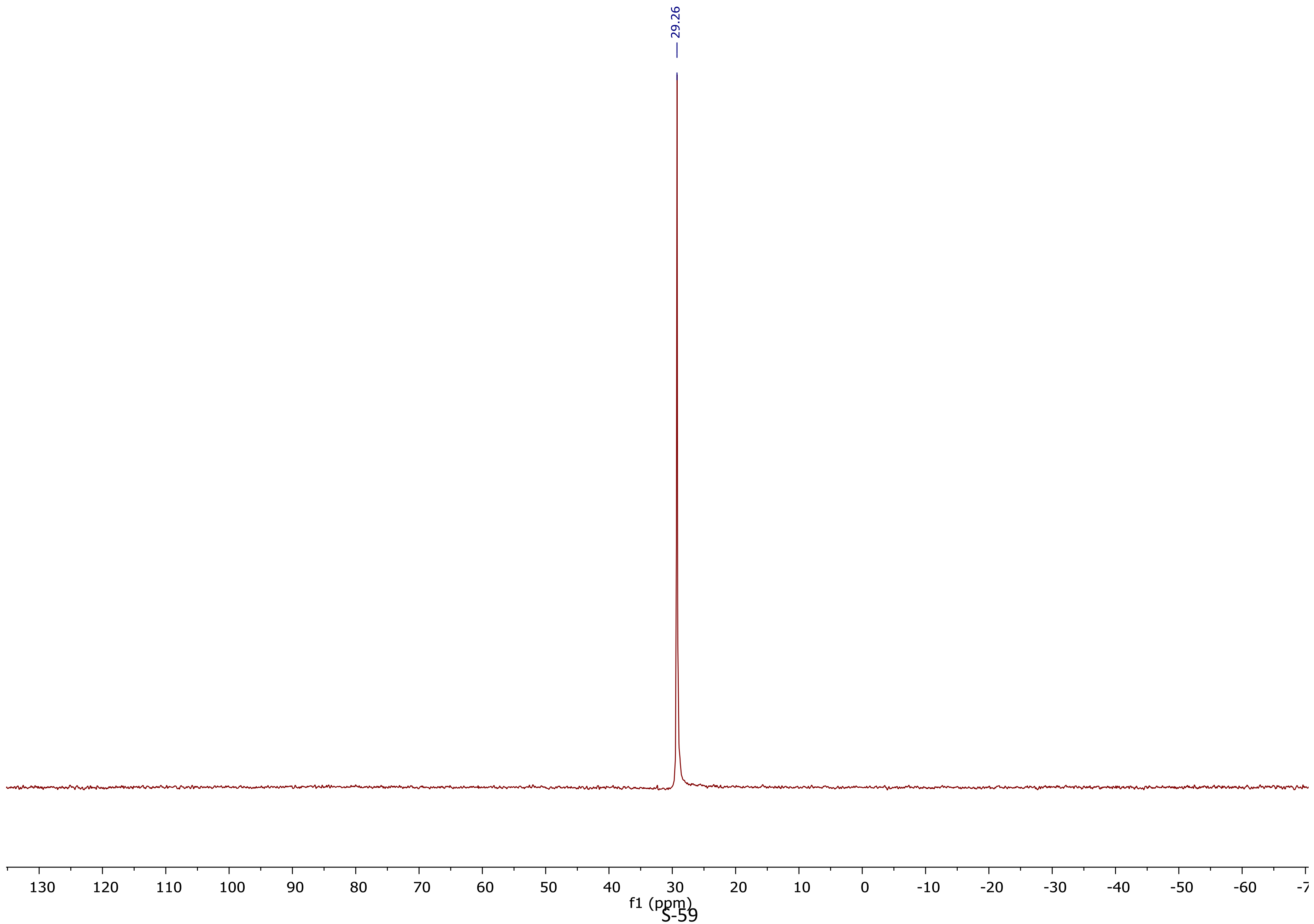
29.26
130
120
110
100
90
80
70
60
50
40
30
20
10
0
-10
-20
-30
-40
-50
-60
f1 (ppm)

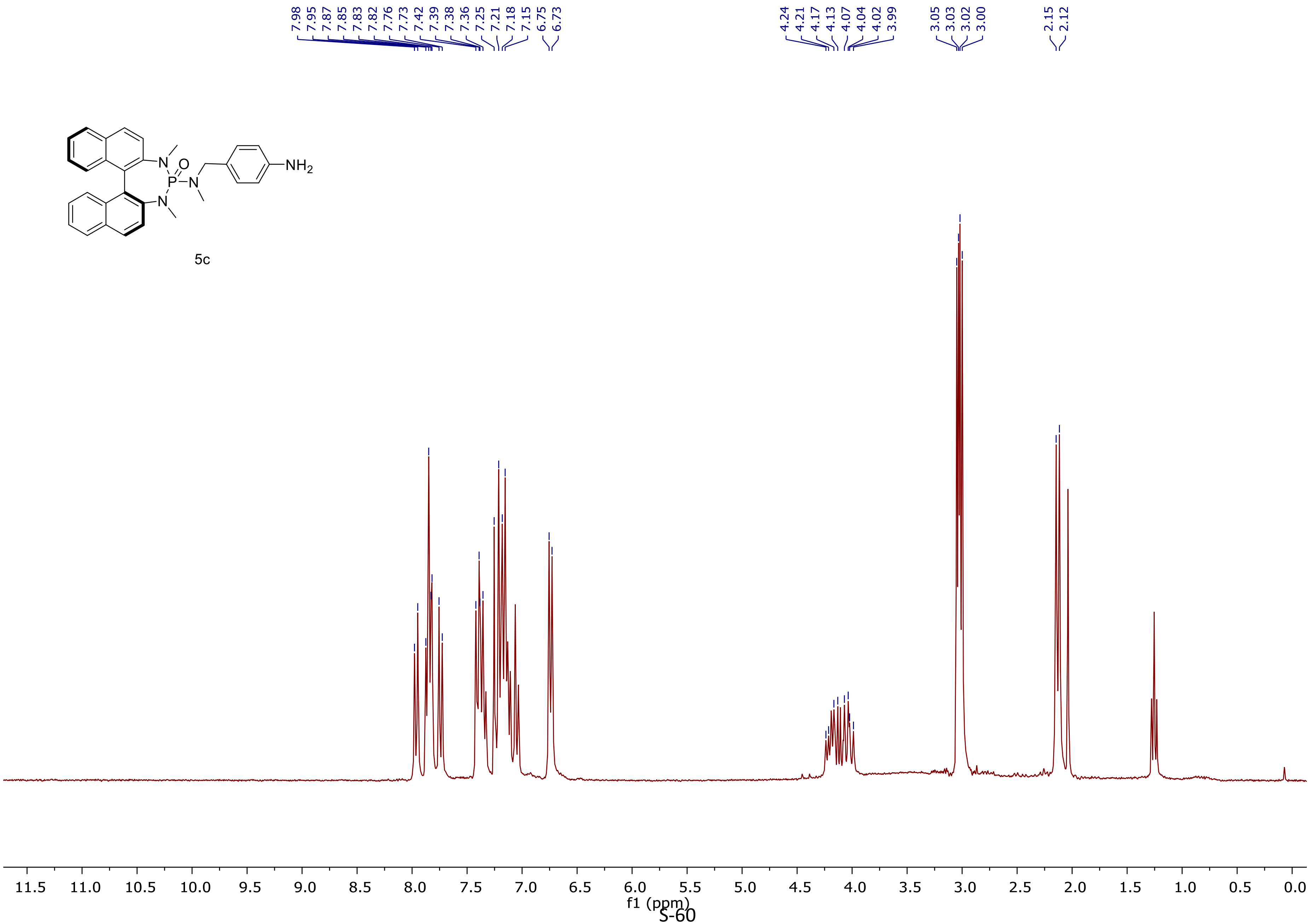
7.98
7.95
7.87
7.85
7.83
7.82
7.76
7.73
7.42
7.39
7.38
7.36
7.25
7.21
7.18
7.15
6.75
6.73
4.24
4.21
4.17
4.13
4.07
4.04
4.02
3.99
3.05
3.03
3.02
3.00
2.15
2.12
N
O
P
N
N
NH2
5c
11.5 11.0 10.5 10.0 9.5 9.0 8.5 8.0 7.5 7.0 6.5 6.0 5.5 5.0 4.5 4.0 3.5 3.0 2.5 2.0 1.5 1.0 0.5 0.0
f1 (ppm)

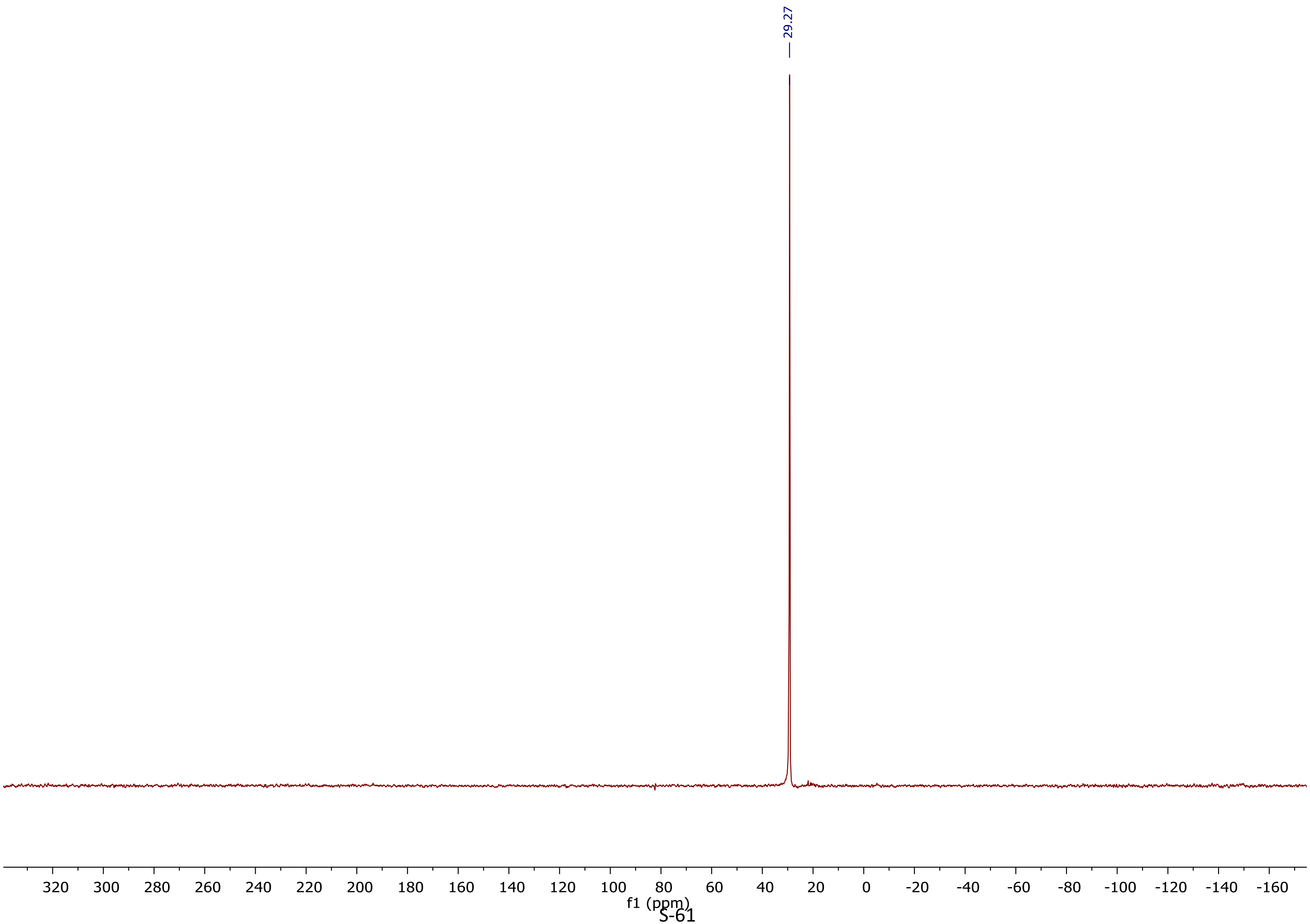
29.27
320 300 280 260 240 220 200 180 160 140 120 100 80 60 40 20 0 -20 -40 -60 -80 -100 -120 -140 -160
f1 (ppm)

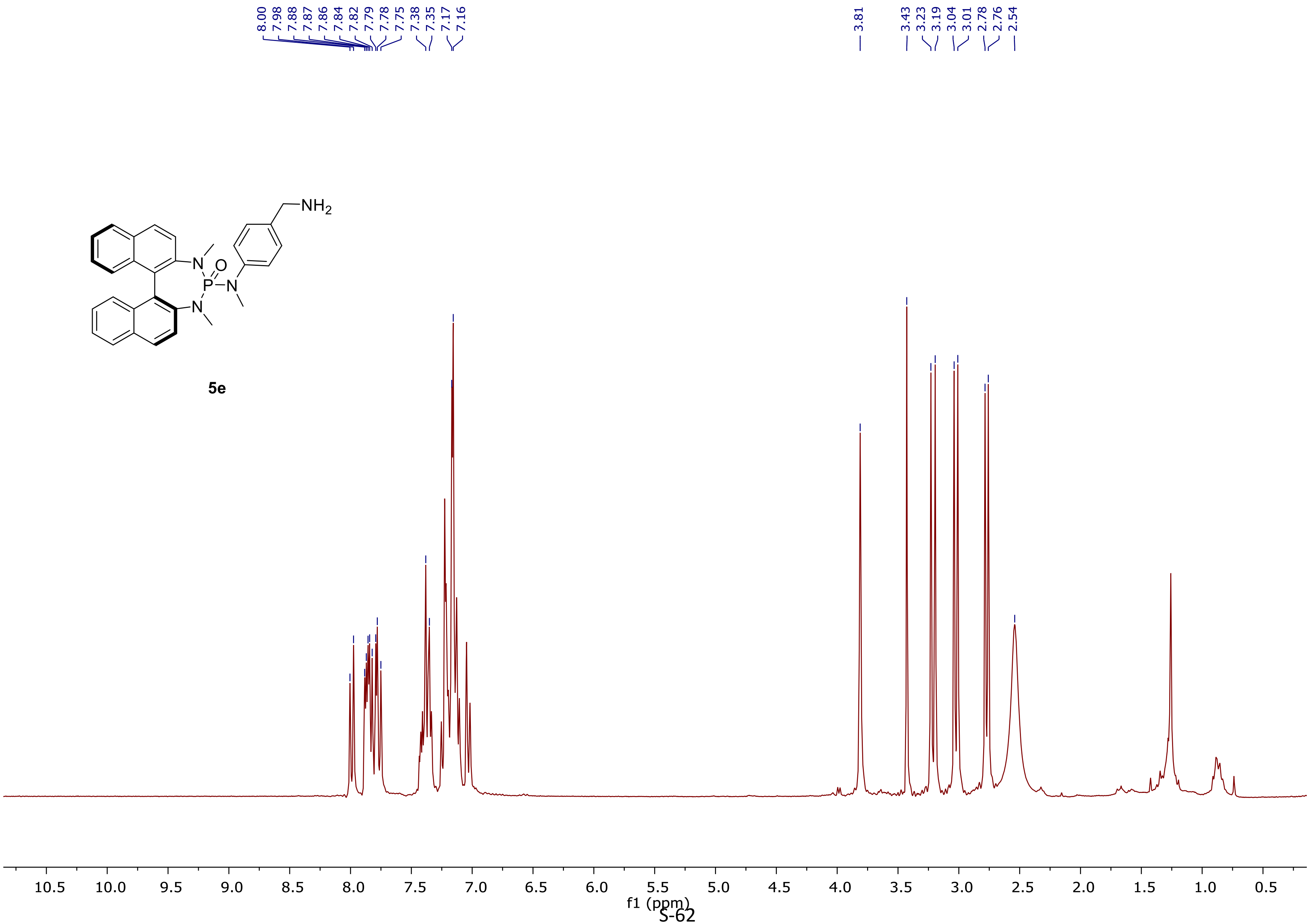
8.00
7.98
7.88
7.87
7.86
7.84
7.82
7.79
7.78
7.75
7.38
7.35
7.17
7.16
3.81
3.43
3.23
3.19
3.04
3.01
2.78
2.76
2.54
NH2
N
O
P
N
N
5e
10.5 10.0 9.5 9.0 8.5 8.0 7.5 7.0 6.5 6.0 5.5 5.0 4.5 4.0 3.5 3.0 2.5 2.0 1.5 1.0 0.5
f1 (ppm)

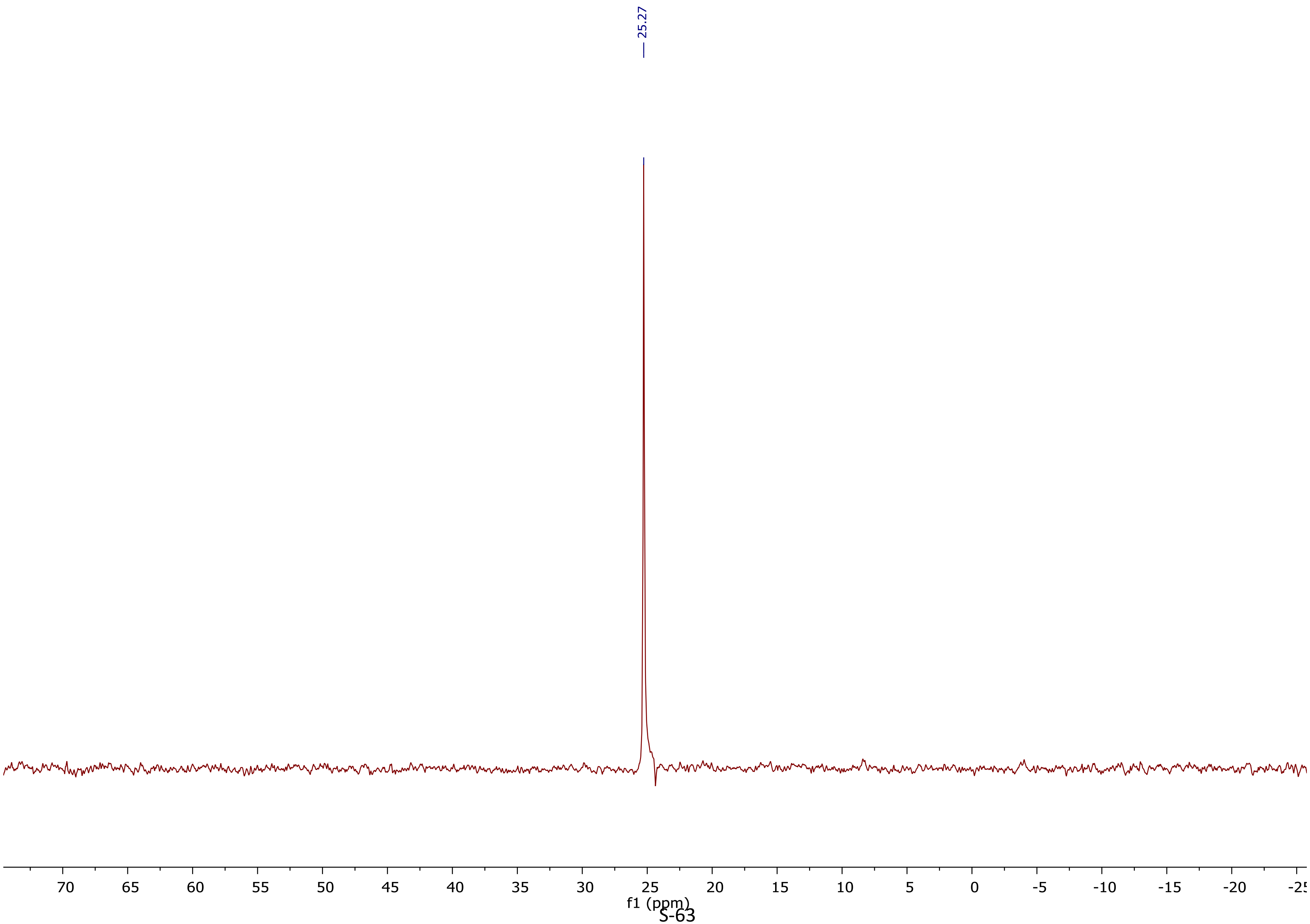
25.27
70
65
60
55
50
45
40
35
30
25
20
15
10
5
0
-5
-10
-15
-20
-25
f1 (ppm)

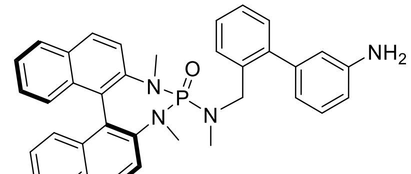
O
N
P
NH2

5f

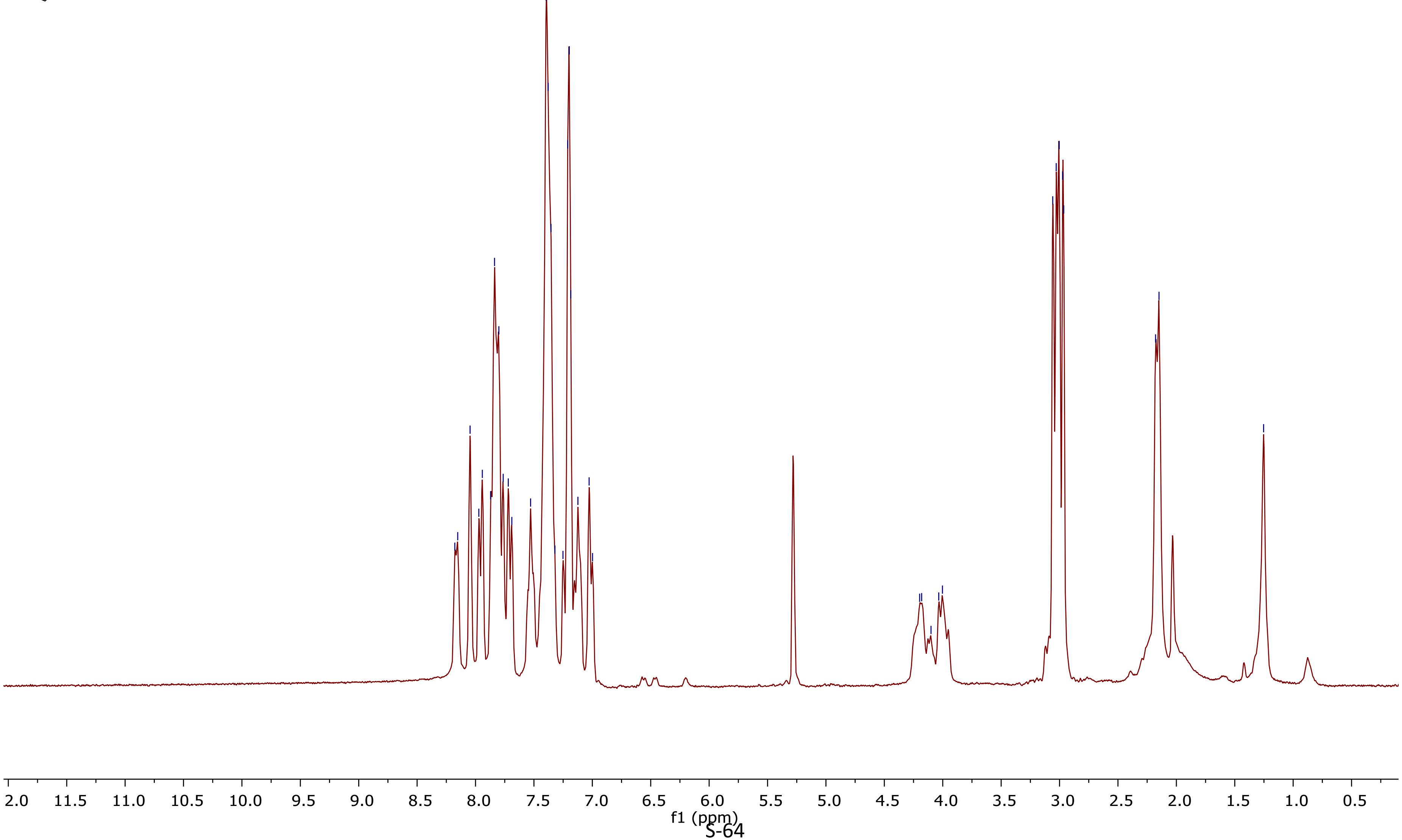
8.18
8.15
8.05
7.97
7.94
7.87
7.84
7.80
7.76
7.72
7.69
7.53
7.40
7.38
7.35
7.32
7.25
7.21
7.20
7.18
7.12
7.03
7.00
4.20
4.18
4.10
4.03
4.00
3.06
3.03
3.00
2.97
2.96
2.18
2.15
1.25
2.0 11.5 11.0 10.5 10.0 9.5 9.0 8.5 8.0 7.5 7.0 6.5 6.0 5.5 5.0 4.5 4.0 3.5 3.0 2.5 2.0 1.5 1.0 0.5
f1 (ppm)

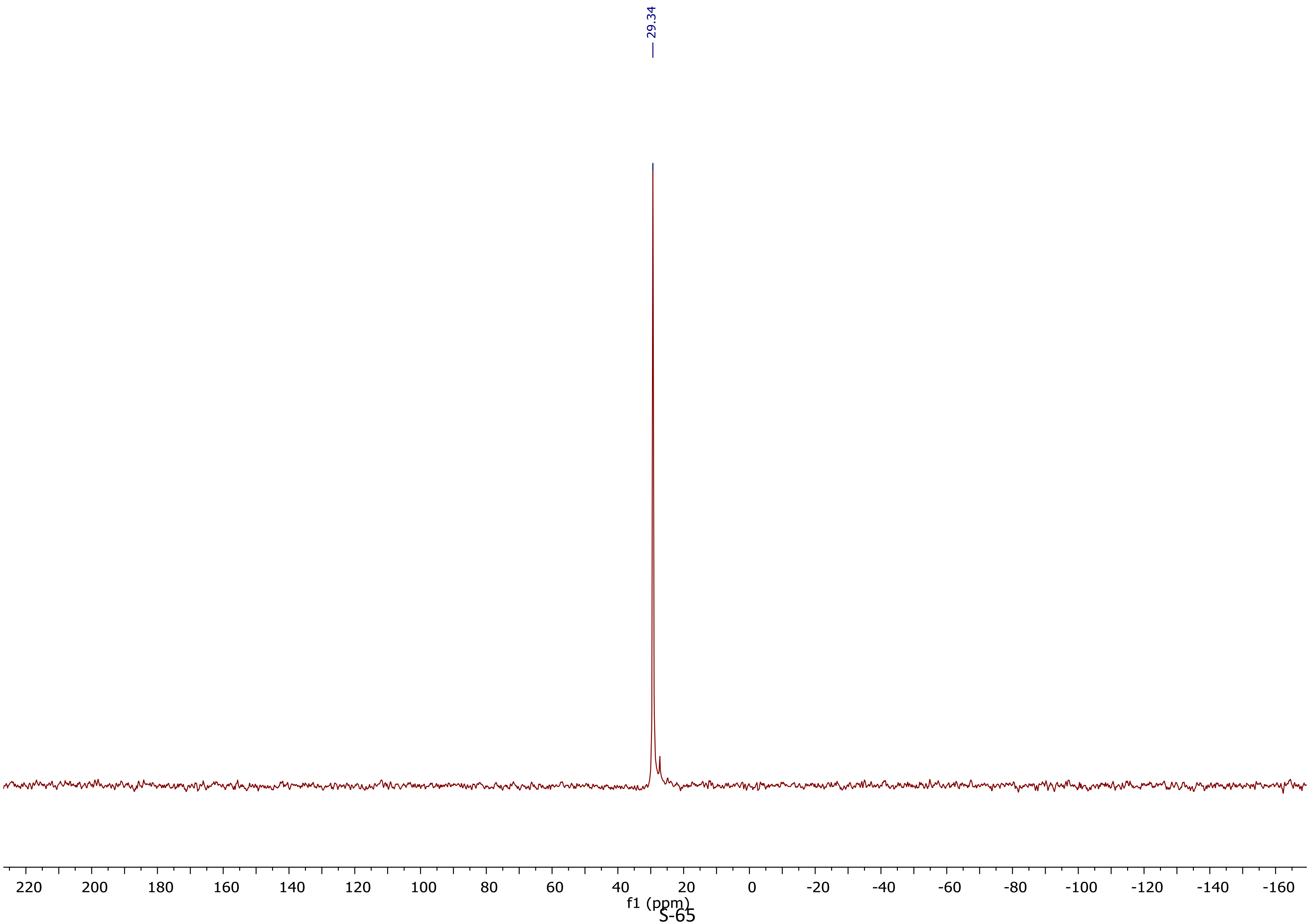
29.34
220
200
180
160
140
120
100
80
60
40
20
0
-20
-40
-60
-80
-100
-120
-140
-160
f1 (ppm)

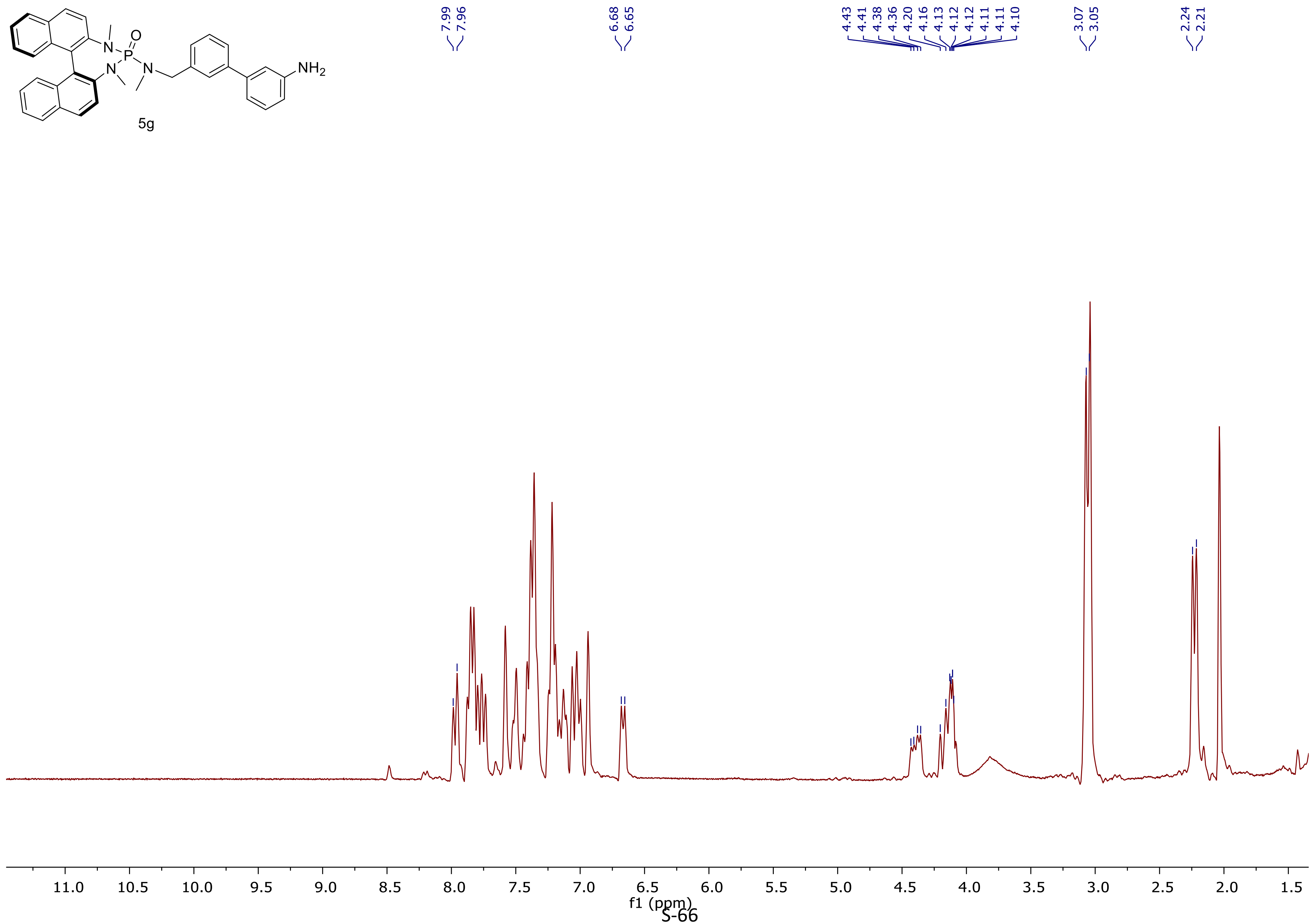
NH2
5g
7.99
7.96
6.68
6.65
4.43
4.41
4.38
4.36
4.20
4.16
4.13
4.12
4.12
4.11
4.11
4.10
3.07
3.05
2.24
2.21
11.0
10.5
10.0
9.5
9.0
8.5
8.0
7.5
7.0
6.5
6.0
5.5
5.0
4.5
4.0
3.5
3.0
2.5
2.0
1.5
f1 (ppm)

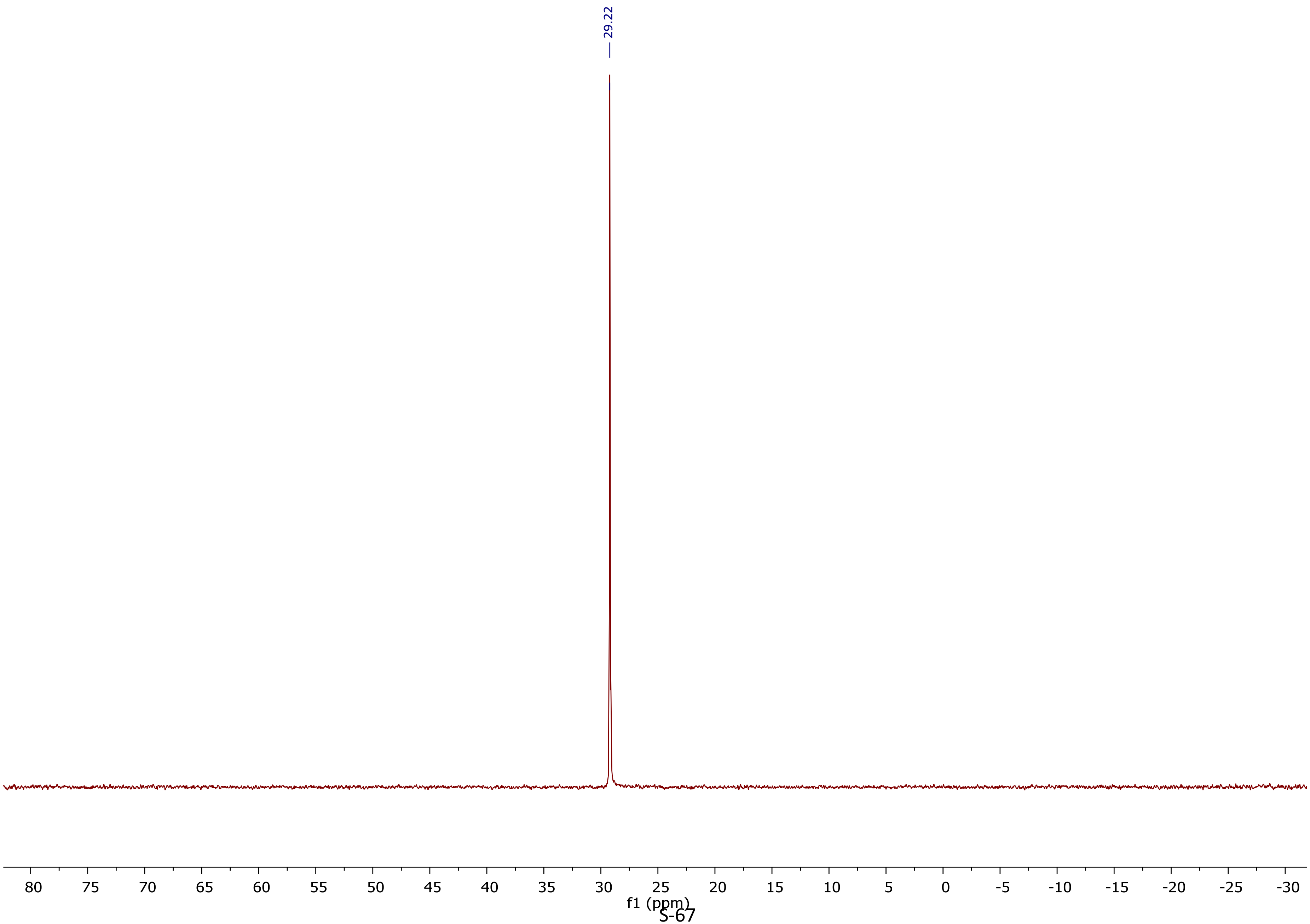
29.22
80 75 70 65 60 55 50 45 40 35 30 25 20 15 10 5 0 -5 -10 -15 -20 -25 -30
f1 (ppm)

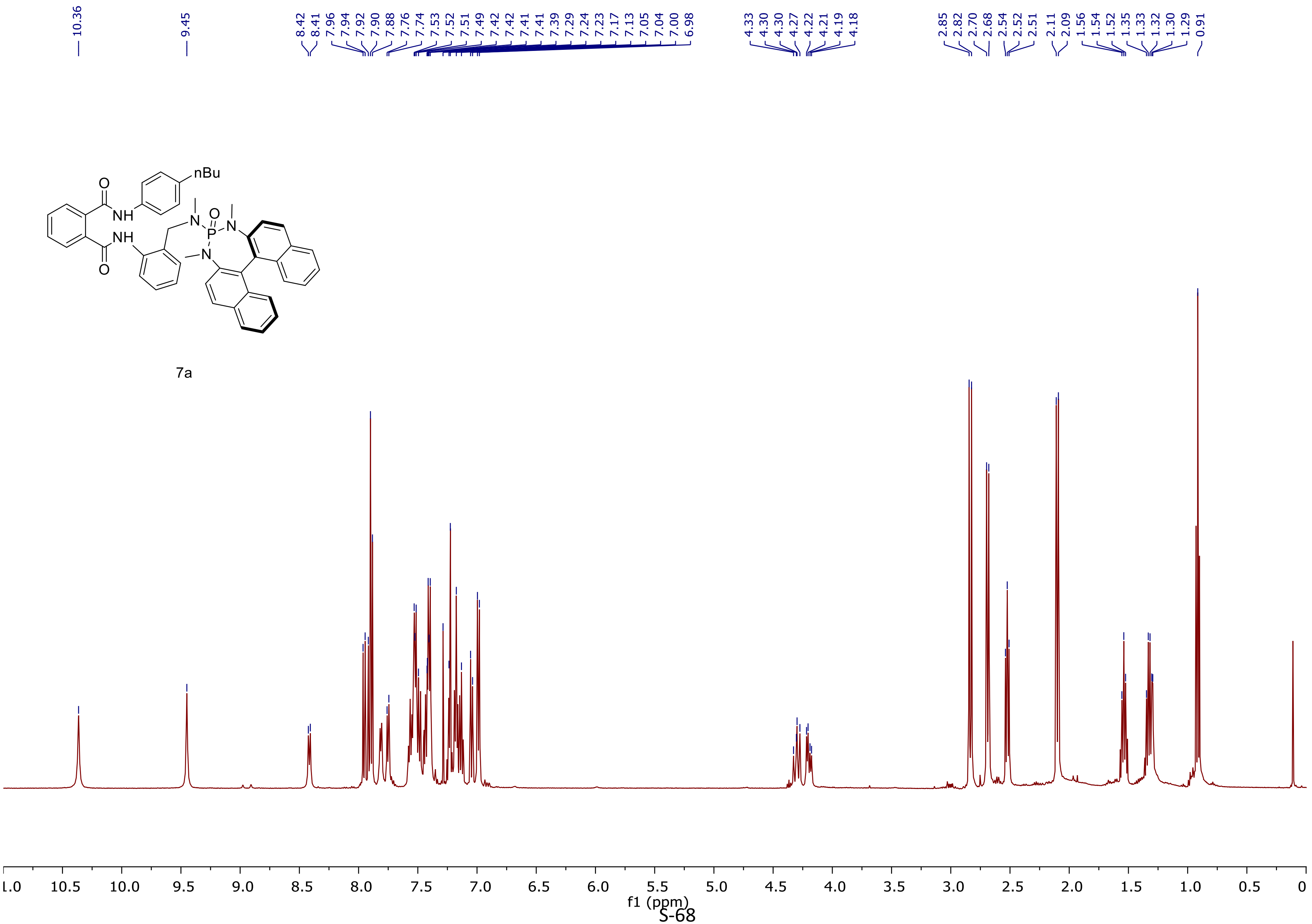
10.36
9.45
8.42
8.41
7.96
7.94
7.92
7.90
7.88
7.76
7.74
7.53
7.52
7.51
7.49
7.42
7.42
7.41
7.39
7.29
7.24
7.23
7.17
7.13
7.05
7.04
7.00
6.98
4.33
4.30
4.30
4.27
4.22
4.21
4.19
4.18
2.85
2.82
2.70
2.68
2.54
2.52
2.51
2.11
2.09
1.56
1.54
1.52
1.35
1.33
1.32
1.30
1.29
0.91
nBu
O
NH
NH
O
N
O
P
N
N
7a
f1 (ppm)

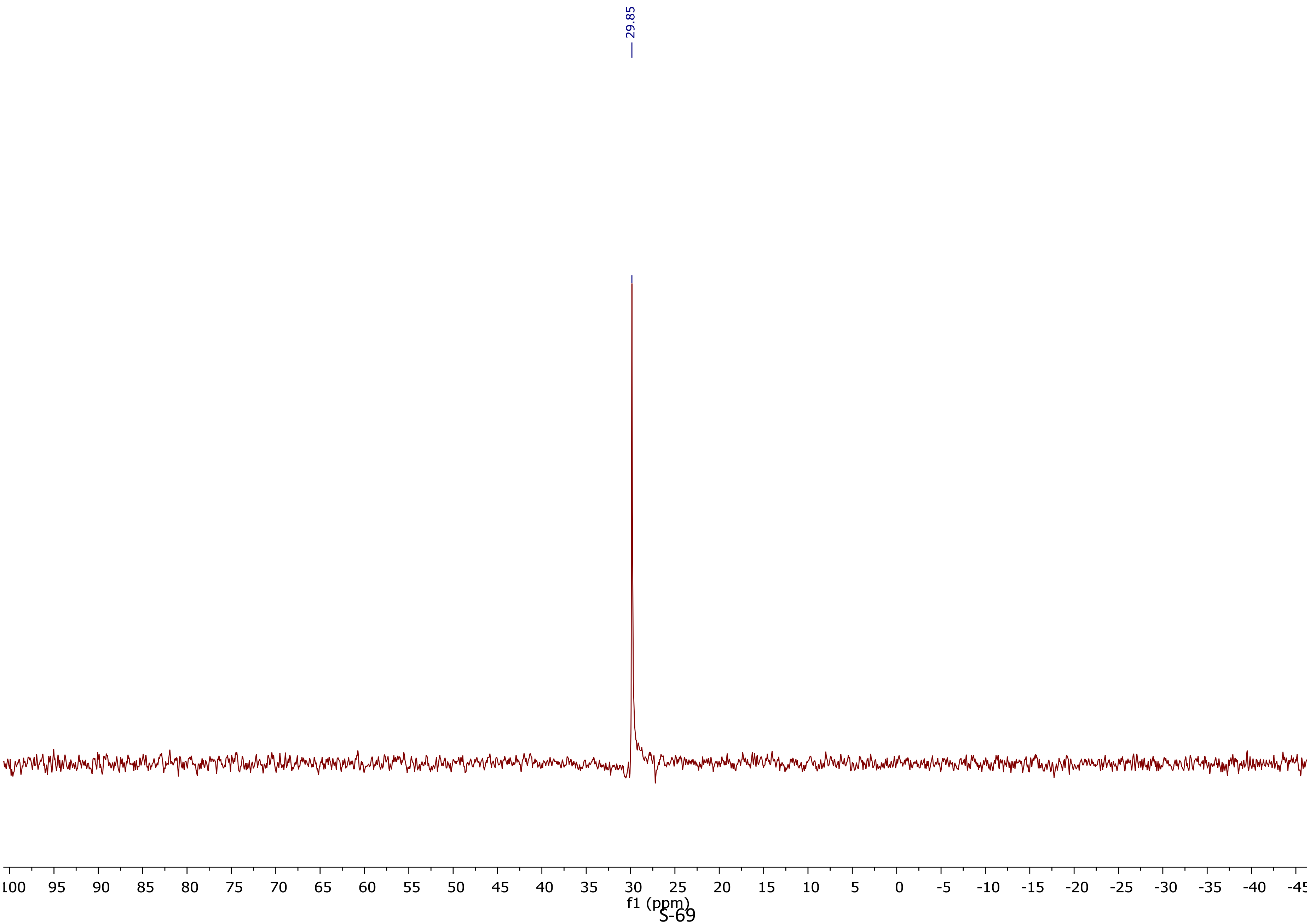
29.85
100 95 90 85 80 75 70 65 60 55 50 45 40 35 30 25 20 15 10 5 0 -5 -10 -15 -20 -25 -30 -35 -40 -45
f1 (ppm)

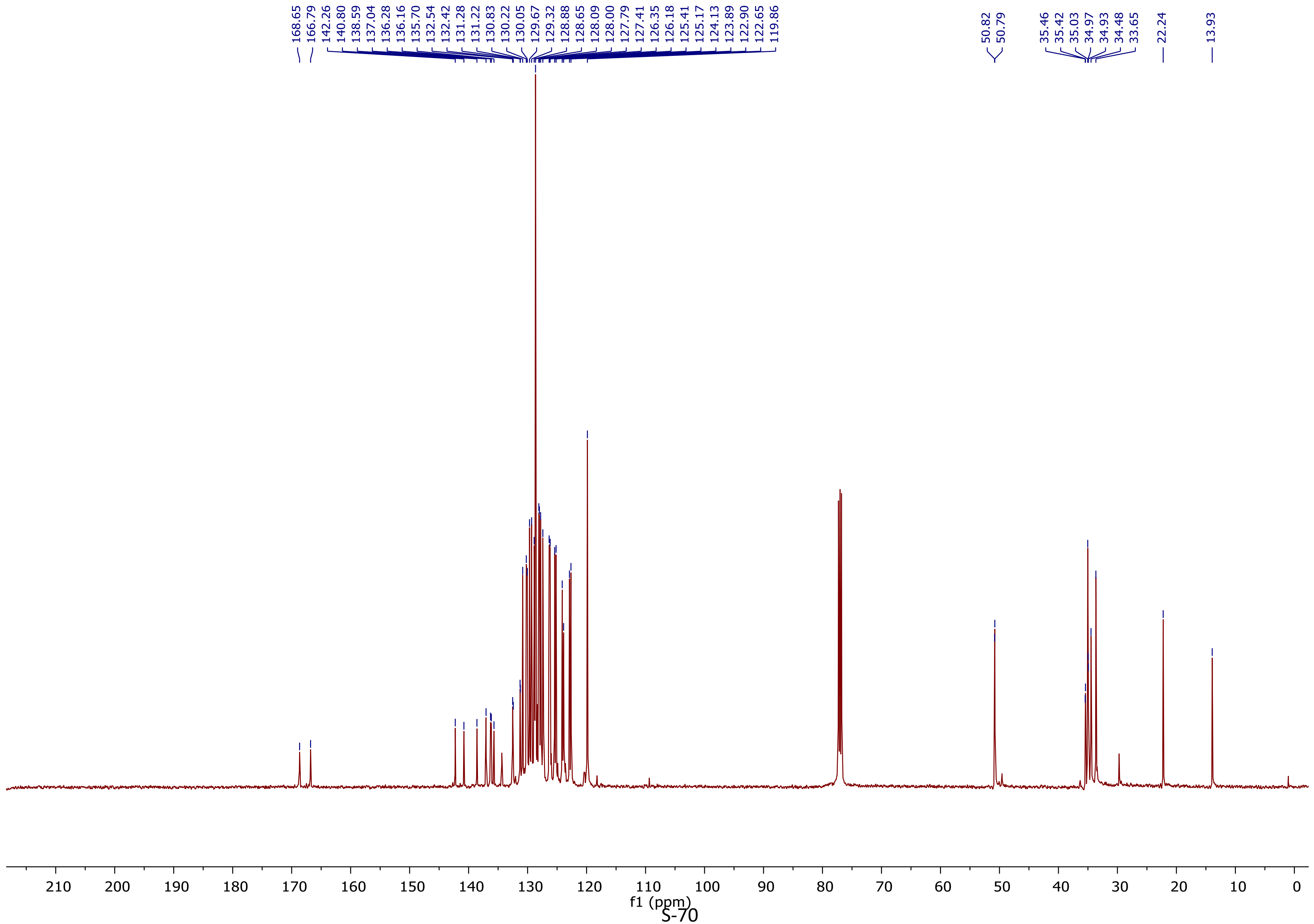
168.65
166.79
142.26
140.80
138.59
137.04
136.28
136.16
135.70
132.54
132.42
131.28
131.22
130.83
130.22
130.05
129.67
129.32
128.88
128.65
128.09
128.00
127.79
127.41
126.35
126.18
125.41
125.17
124.13
123.89
122.90
122.65
119.86
50.82
50.79
35.46
35.42
35.03
34.97
34.93
34.48
33.65
22.24
13.93
210 200 190 180 170 160 150 140 130 120 110 100 90 80 70 60 50 40 30 20 10 0
f1 (ppm)

COSY

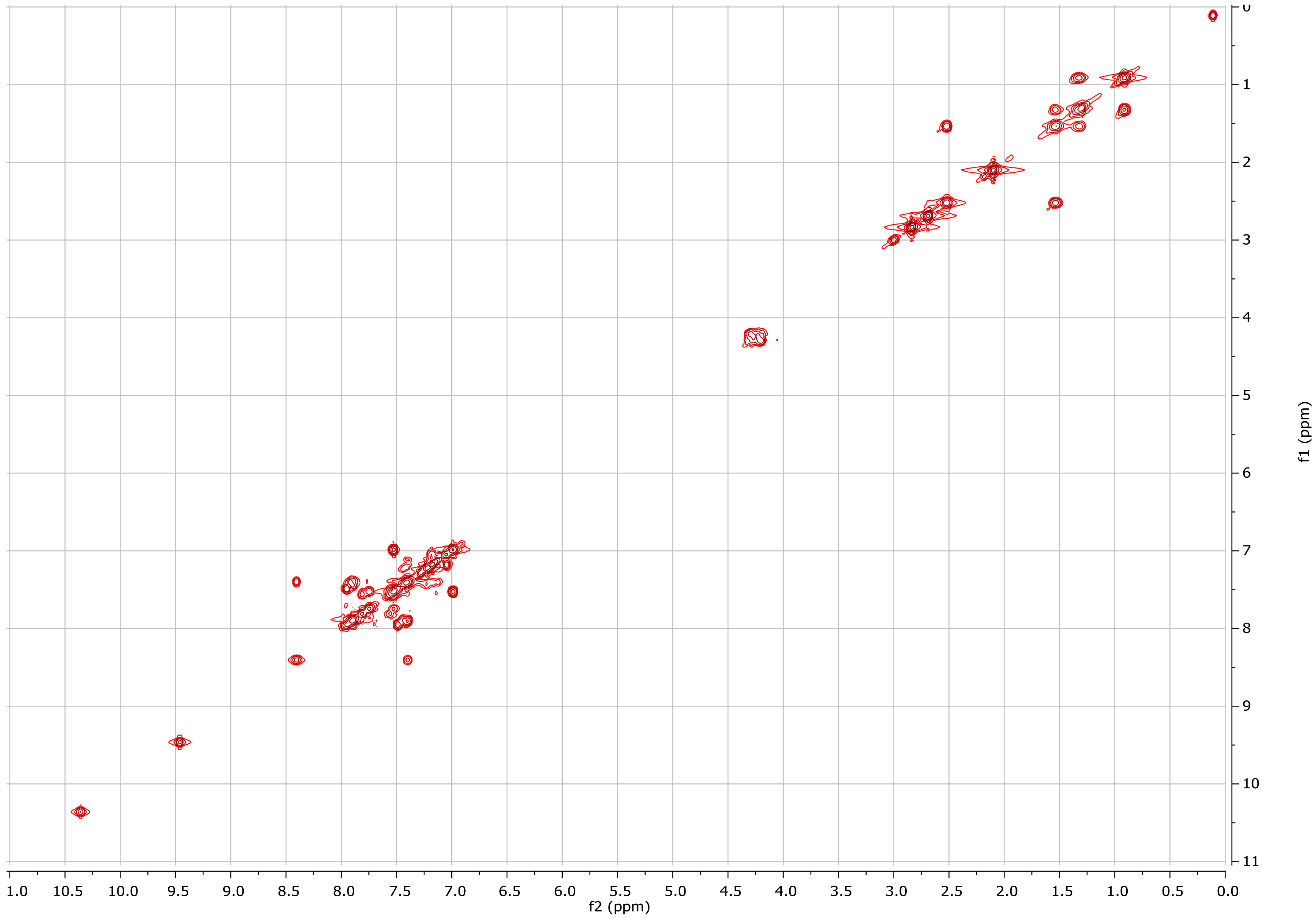

HSQC

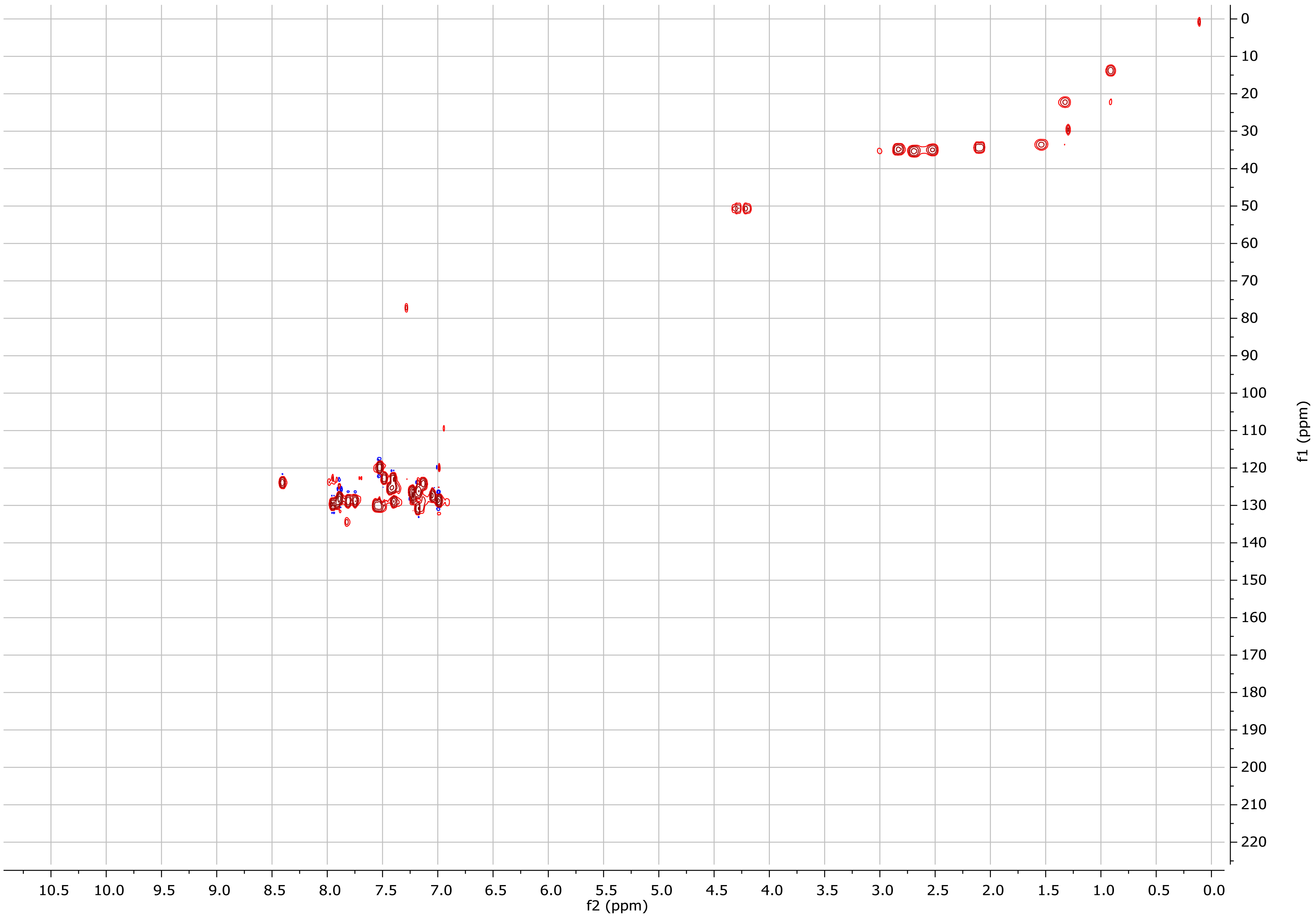

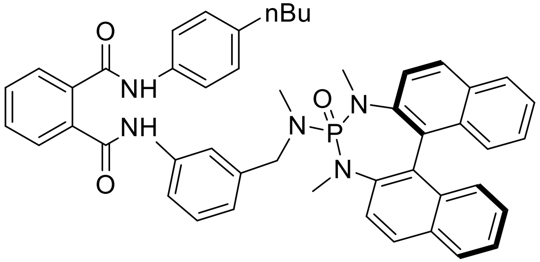
nBu
O
NH
NH
O
O
N
P
N
N

7b

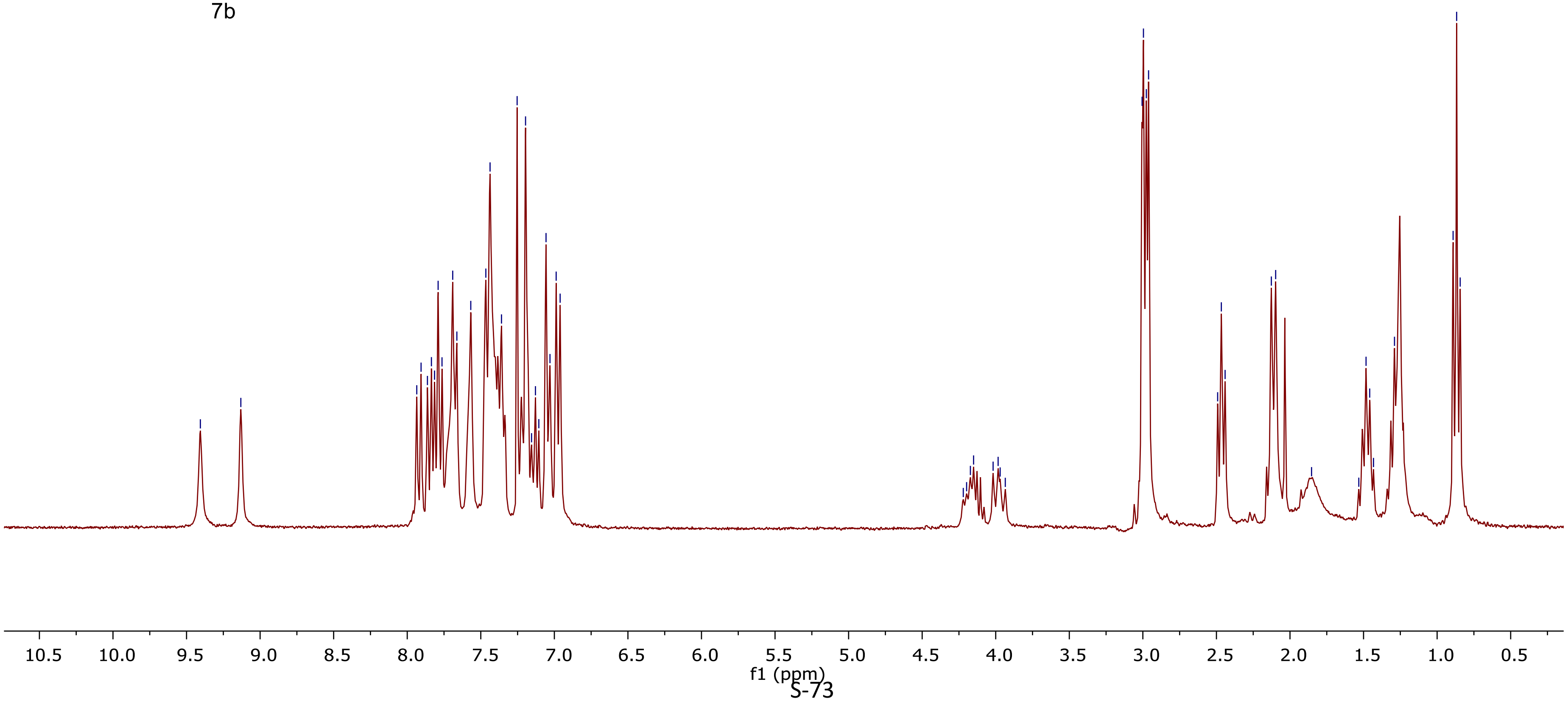
9.41
9.13
7.94
7.91
7.86
7.84
7.81
7.79
7.76
7.69
7.66
7.57
7.47
7.44
7.36
7.25
7.20
7.16
7.13
7.11
7.06
7.03
6.99
6.96
4.22
4.20
4.17
4.15
4.02
3.98
3.97
3.94
3.01
3.00
2.98
2.96
2.49
2.47
2.44
2.13
2.10
1.85
1.53
1.48
1.46
1.43
1.29
0.89
0.87
0.84
10.5
10.0
9.5
9.0
8.5
8.0
7.5
7.0
6.5
6.0
5.5
5.0
4.5
4.0
3.5
3.0
2.5
2.0
1.5
1.0
0.5
f1 (ppm)

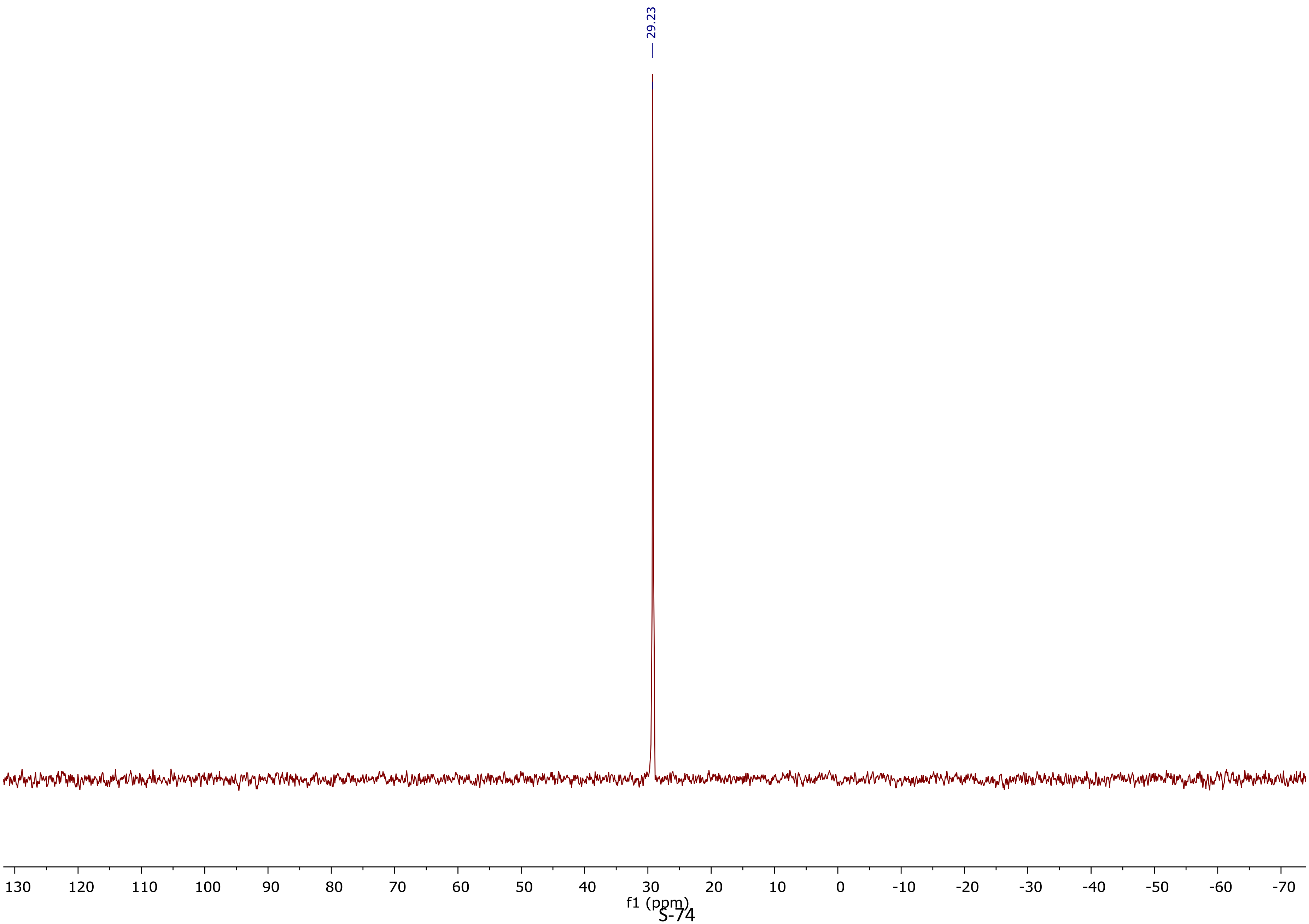
29.23
130
120
110
100
90
80
70
60
50
40
30
20
10
0
-10
-20
-30
-40
-50
-60
-70
f1 (ppm)

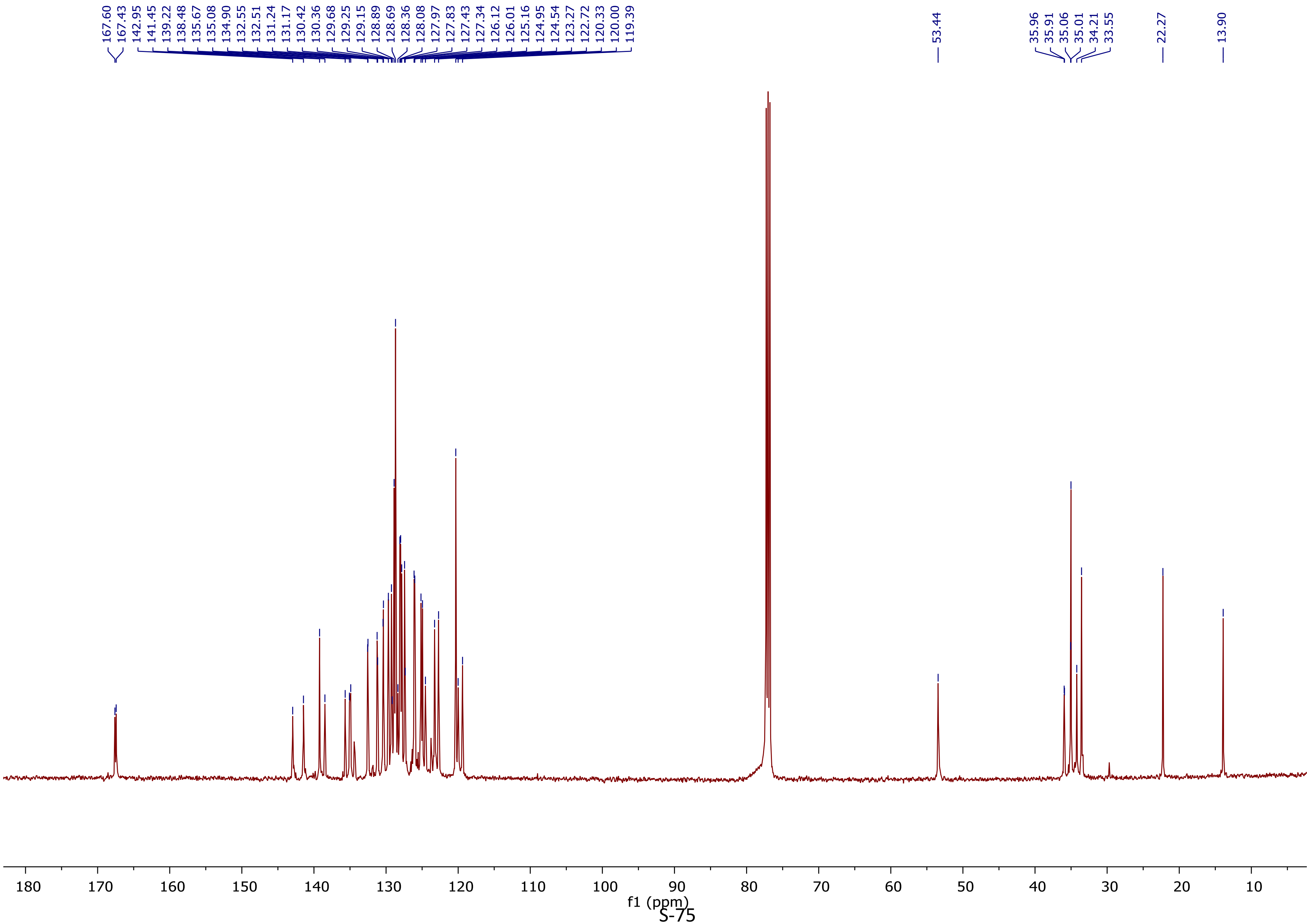
167.60
167.43
142.95
141.45
139.22
138.48
135.67
135.08
134.90
132.55
132.51
131.24
131.17
130.42
130.36
129.68
129.25
129.15
128.89
128.69
128.36
128.08
127.97
127.83
127.43
127.34
126.12
126.01
125.16
124.95
124.54
123.27
122.72
120.33
120.00
119.39
53.44
35.96
35.91
35.06
35.01
34.21
33.55
22.27
13.90
180 170 160 150 140 130 120 110 100 90 80 70 60 50 40 30 20 10
f1 (ppm)

COSY

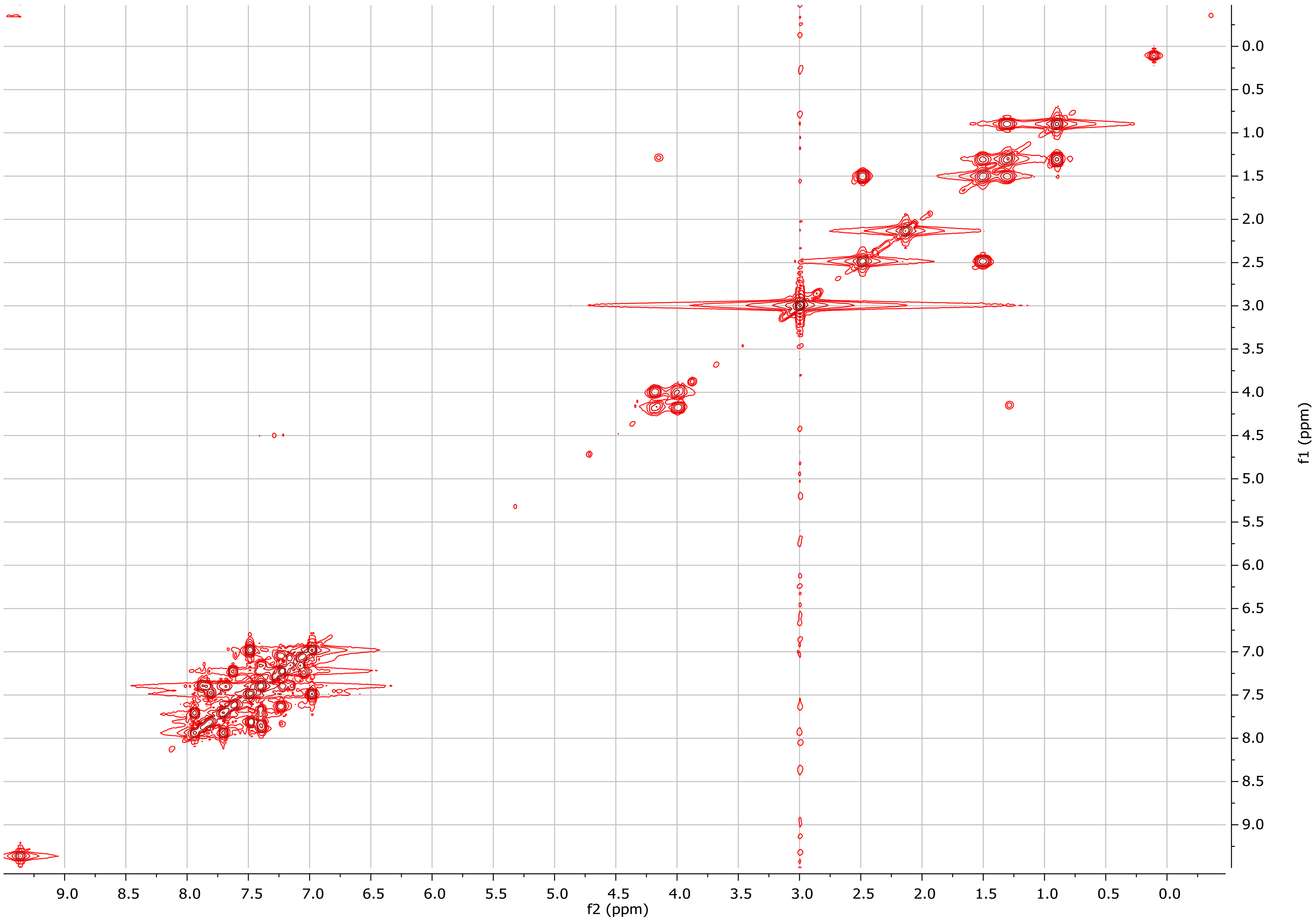

9.0 8.5 8.0 7.5 7.0 6.5 6.0 5.5 5.0 4.5 4.0 3.5 3.0 2.5 2.0 1.5 1.0 0.5 0.0
f2 (ppm)
0.0 0.5 1.0 1.5 2.0 2.5 3.0 3.5 4.0 4.5 5.0 5.5 6.0 6.5 7.0 7.5 8.0 8.5 9.0
f1 (ppm)

HMQC

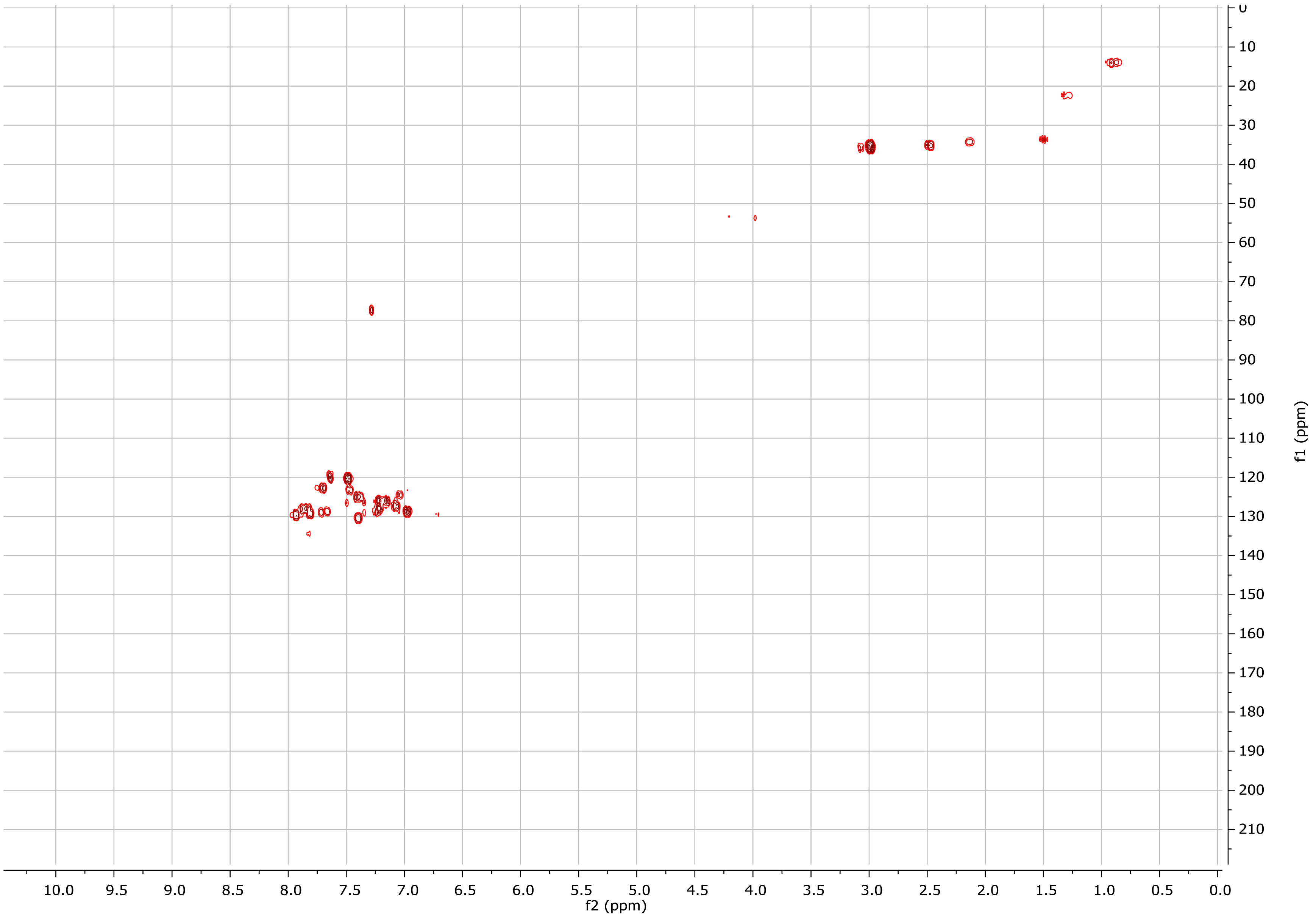

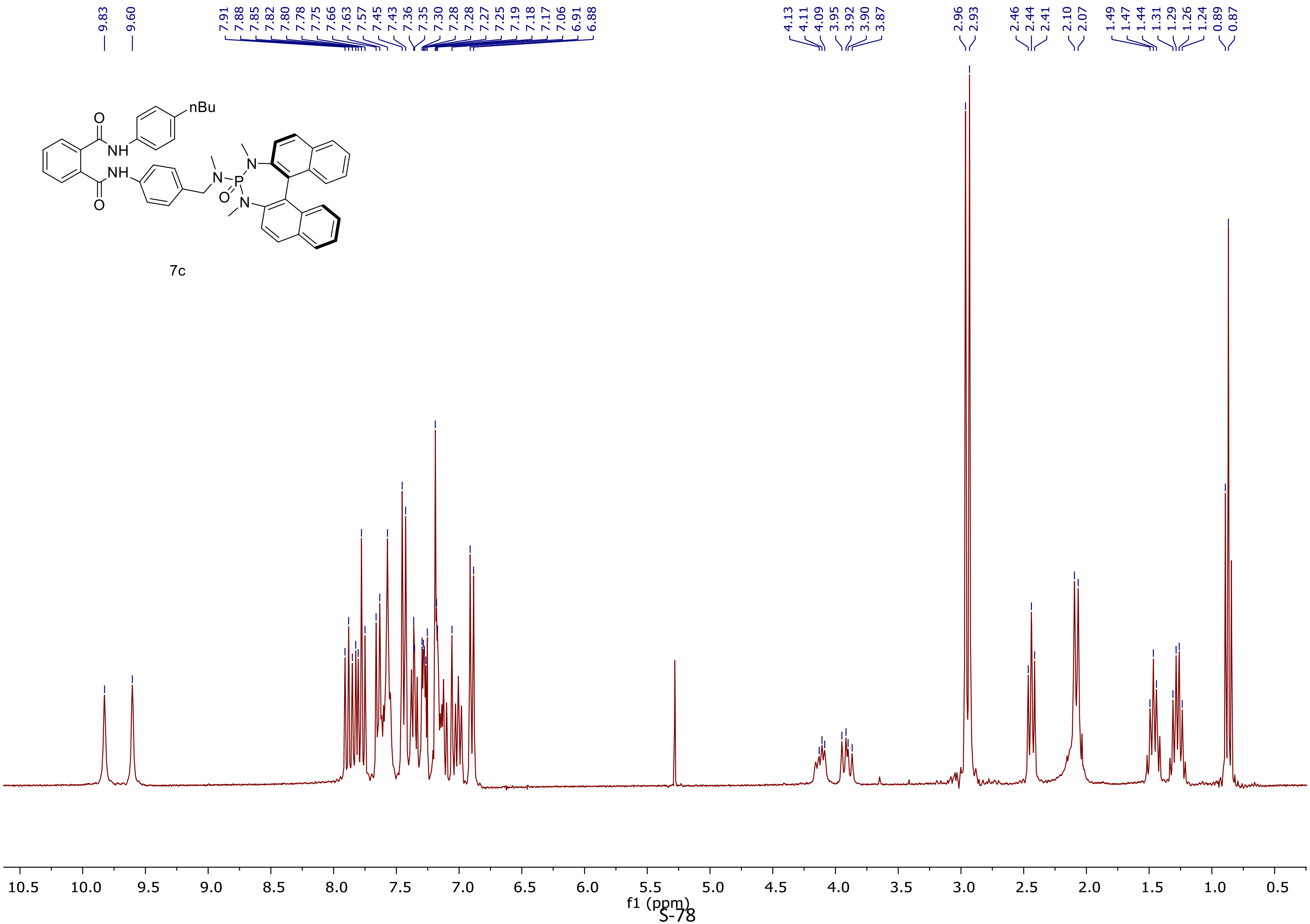
9.83
9.60
7.91
7.88
7.85
7.82
7.80
7.78
7.75
7.66
7.63
7.57
7.45
7.43
7.36
7.35
7.30
7.28
7.28
7.27
7.25
7.19
7.18
7.17
7.06
6.91
6.88
4.13
4.11
4.09
3.95
3.92
3.90
3.87
2.96
2.93
2.46
2.44
2.41
2.10
2.07
1.49
1.47
1.44
1.31
1.29
1.26
1.24
0.89
0.87
nBu
O
NH
NH
O
N
P
O
N
N
7c
10.5 10.0 9.5 9.0 8.5 8.0 7.5 7.0 6.5 6.0 5.5 5.0 4.5 4.0 3.5 3.0 2.5 2.0 1.5 1.0 0.5
f1 (ppm)

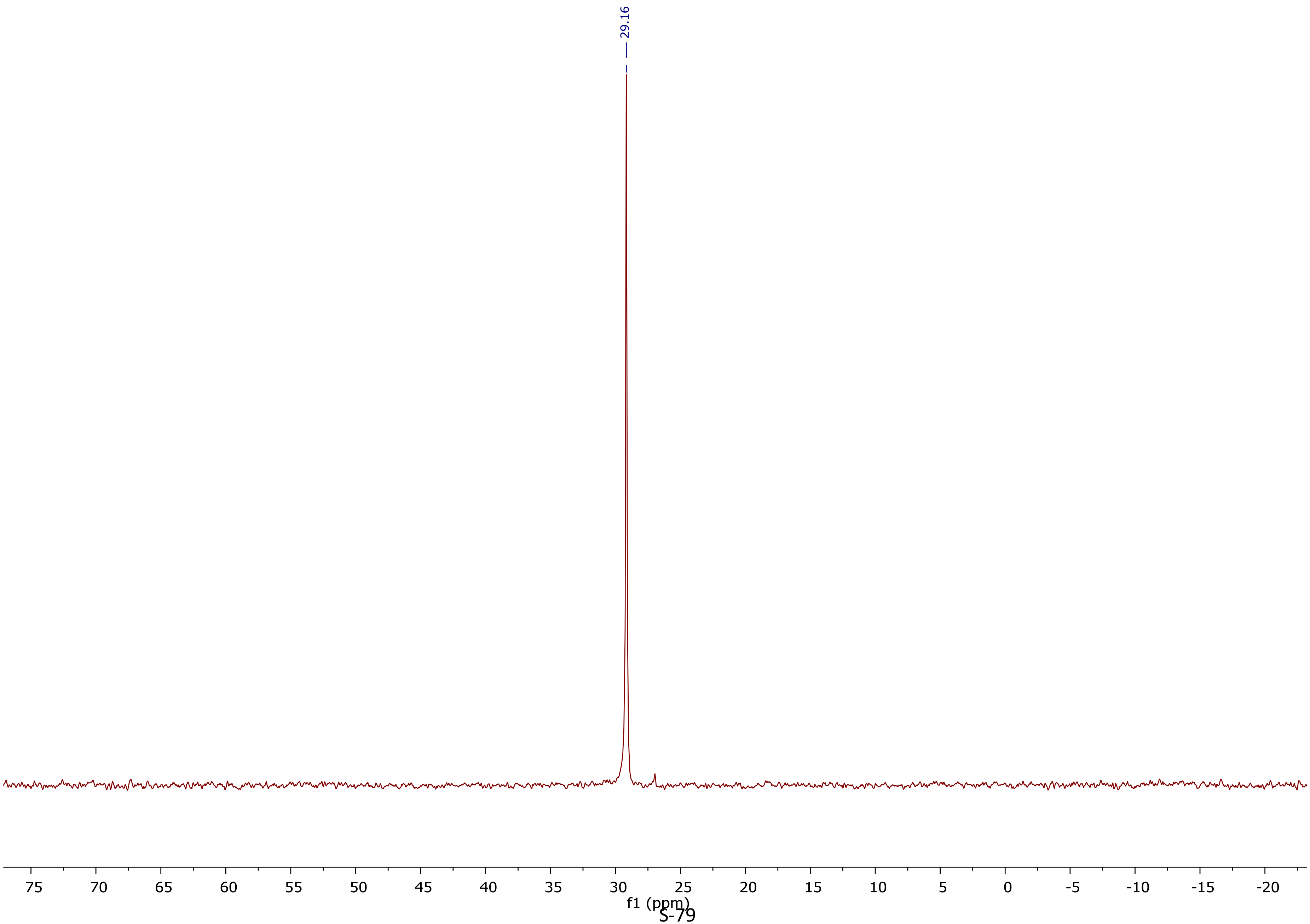
29.16
75
70
65
60
55
50
45
40
35
30
25
20
15
10
5
0
-5
-10
-15
-20
f1 (ppm)

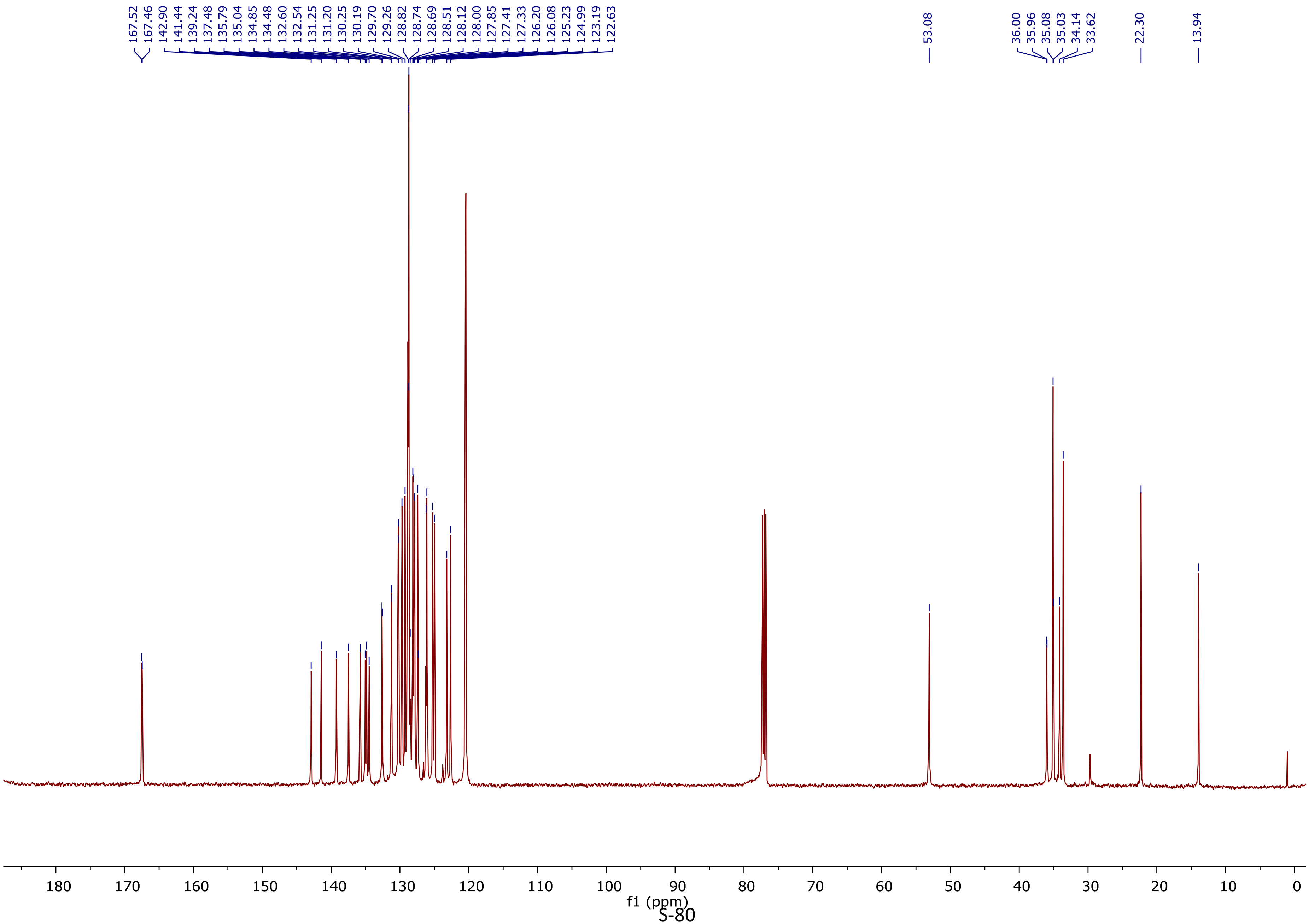
167.52
167.46
142.90
141.44
139.24
137.48
135.79
135.04
134.85
134.48
132.60
132.54
131.25
131.20
130.25
130.19
129.70
129.26
128.82
128.74
128.69
128.51
128.12
128.00
127.85
127.41
127.33
126.20
126.08
125.23
124.99
123.19
122.63
53.08
36.00
35.96
35.08
35.03
34.14
33.62
22.30
13.94
180 170 160 150 140 130 120 110 100 90 80 70 60 50 40 30 20 10 0
f1 (ppm)

COSY

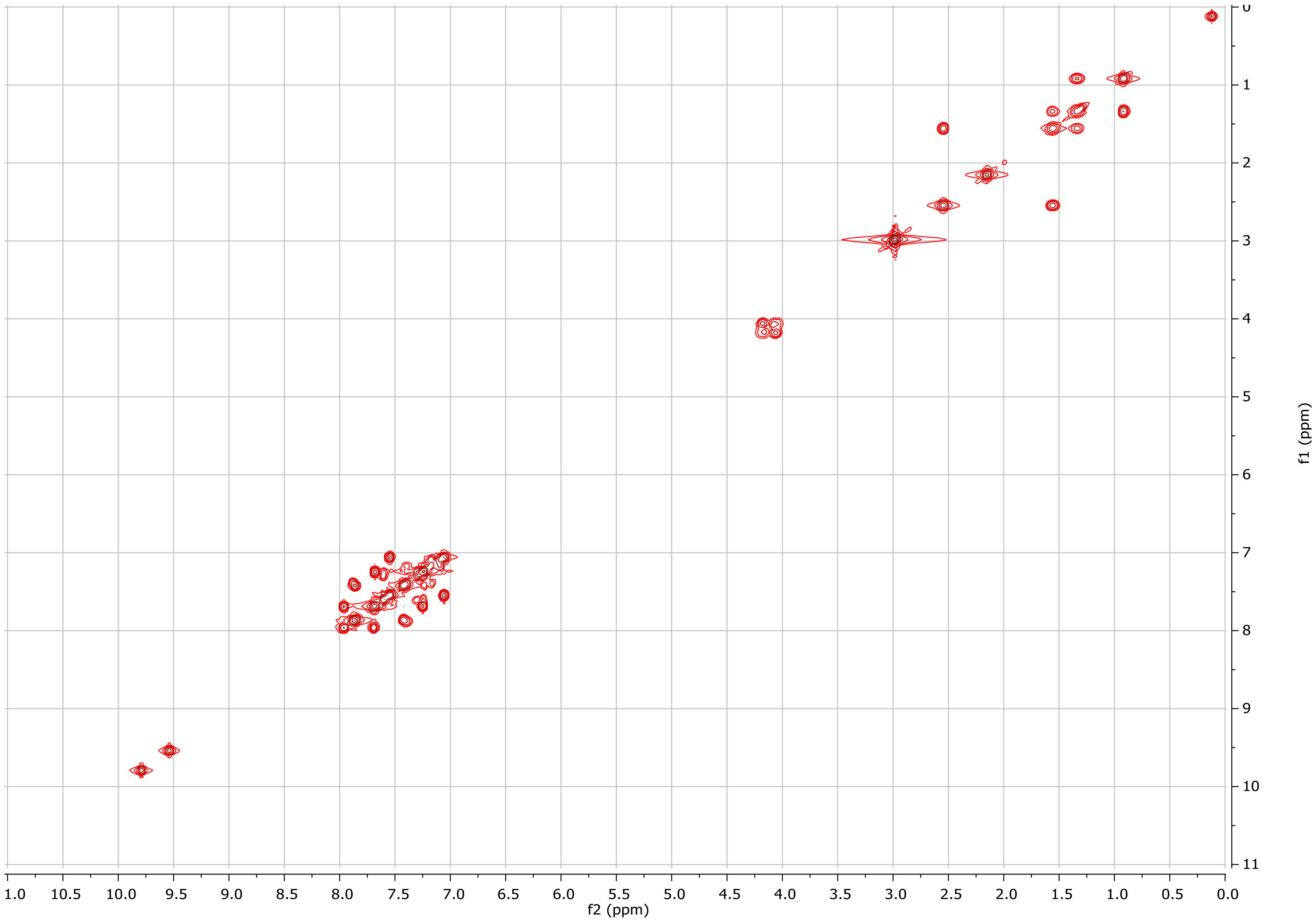

HSQC

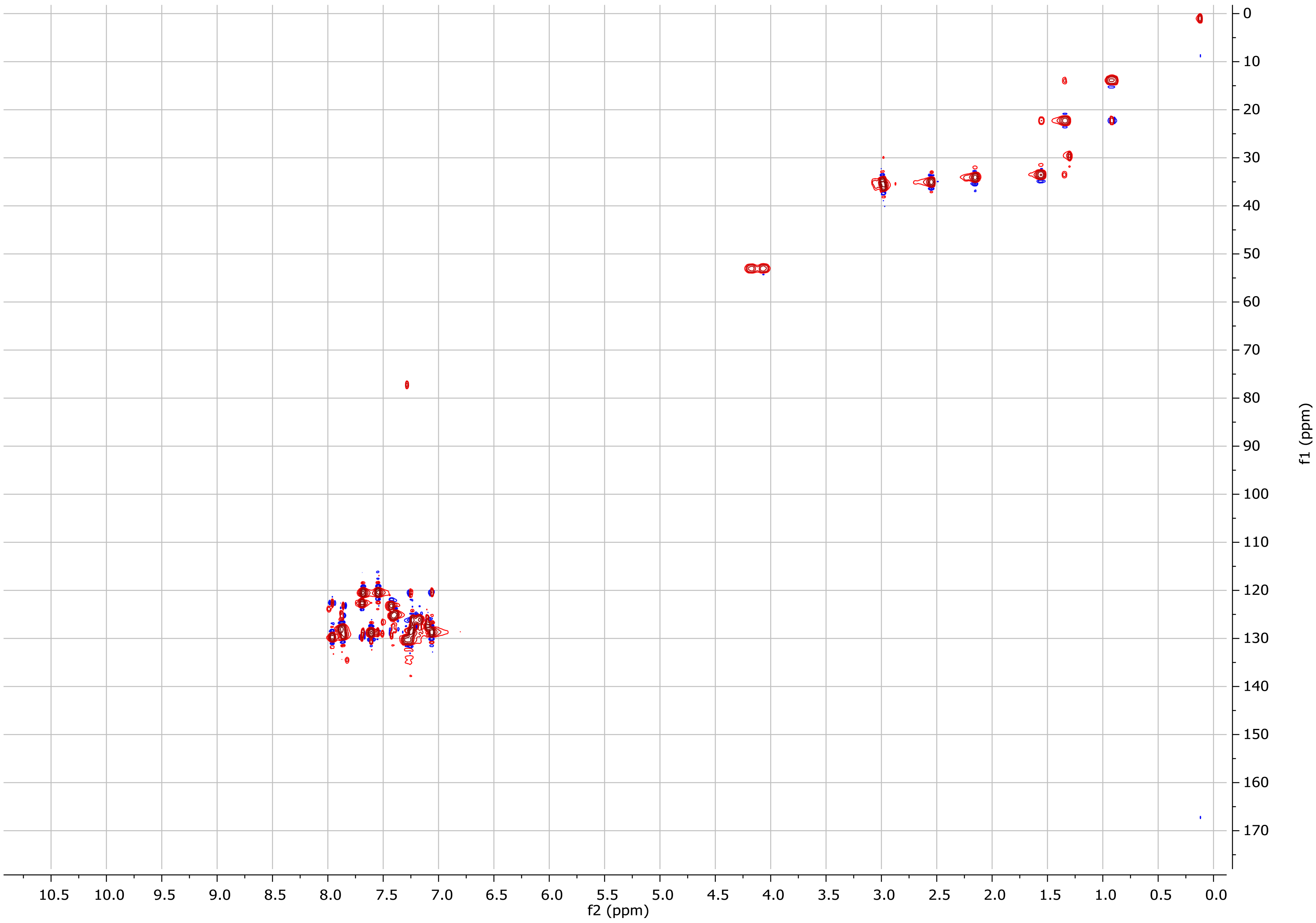

10.5
10.0
9.5
9.0
8.5
8.0
7.5
7.0
6.5
6.0
5.5
5.0
4.5
4.0
3.5
3.0
2.5
2.0
1.5
1.0
0.5
0.0
f2 (ppm)
0
10
20
30
40
50
60
70
80
90
100
110
120
130
140
150
160
170
f1 (ppm)

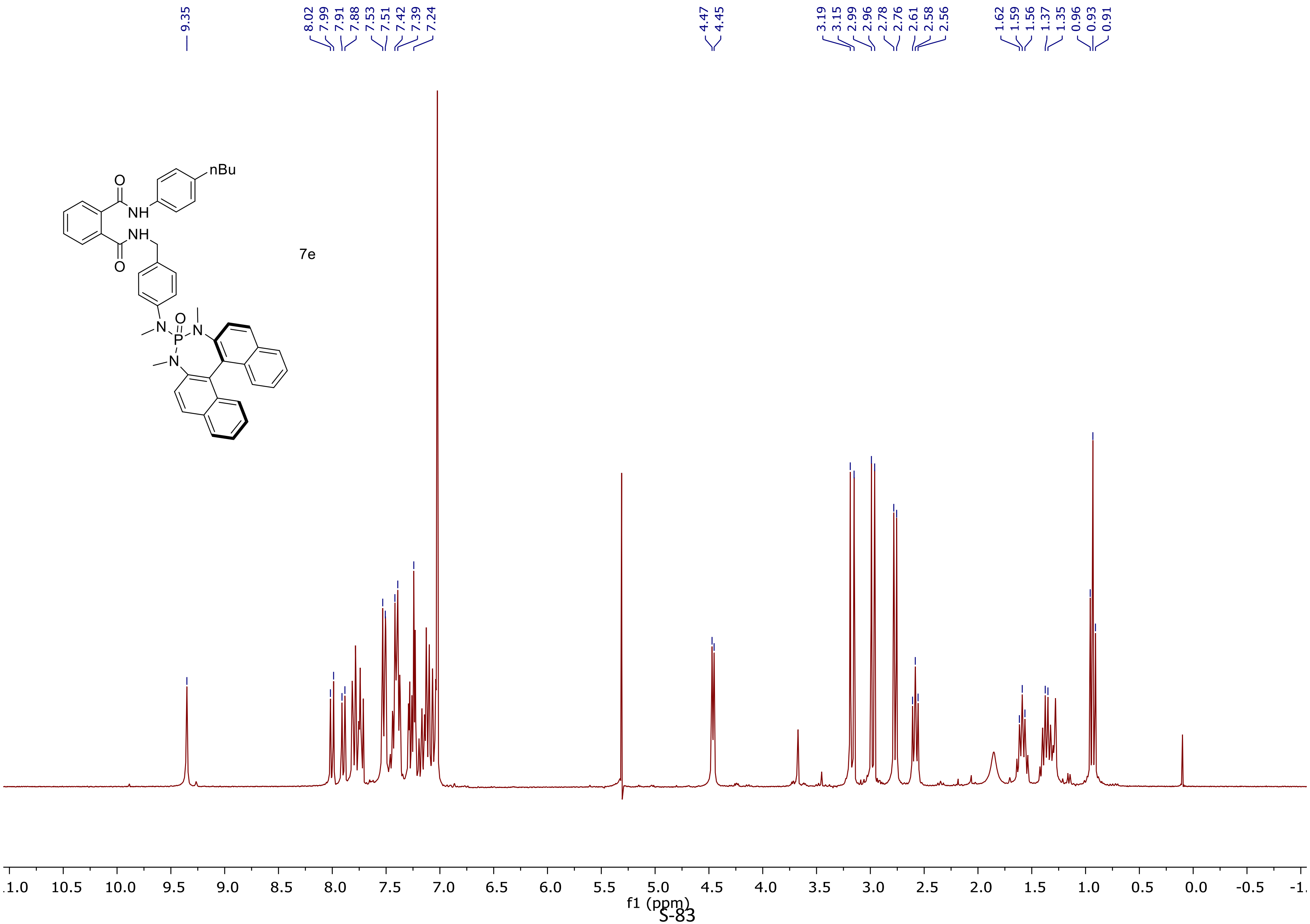
9.35
8.02
7.99
7.91
7.88
7.53
7.51
7.42
7.39
7.24
4.47
4.45
3.19
3.15
2.99
2.96
2.78
2.76
2.61
2.58
2.56
1.62
1.59
1.56
1.37
1.35
0.96
0.93
0.91
nBu
O
NH
NH
O
N
P
O
N
N
7e
f1 (ppm)

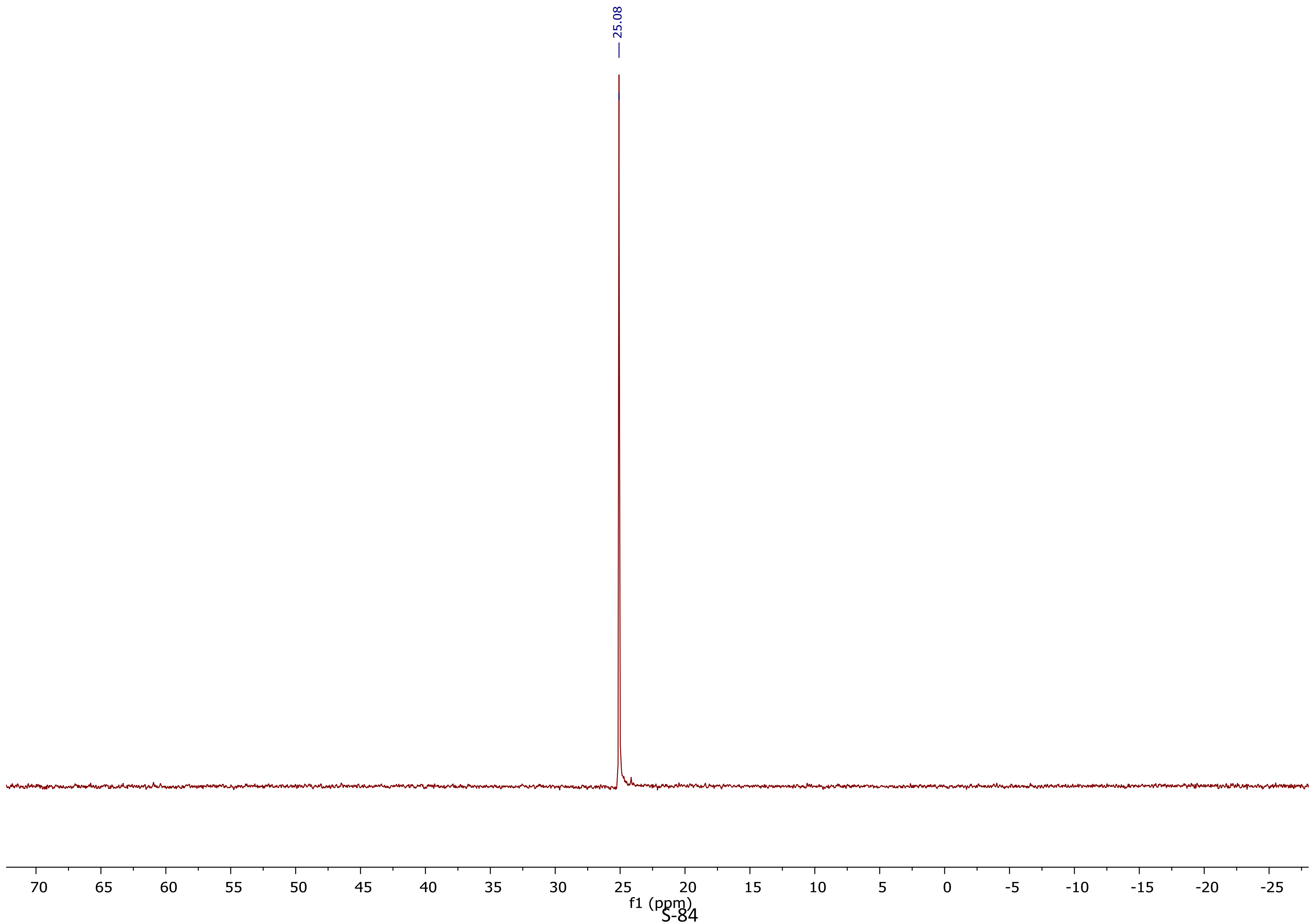
25.08
70
65
60
55
50
45
40
35
30
25
20
15
10
5
0
-5
-10
-15
-20
-25
f1 (ppm)

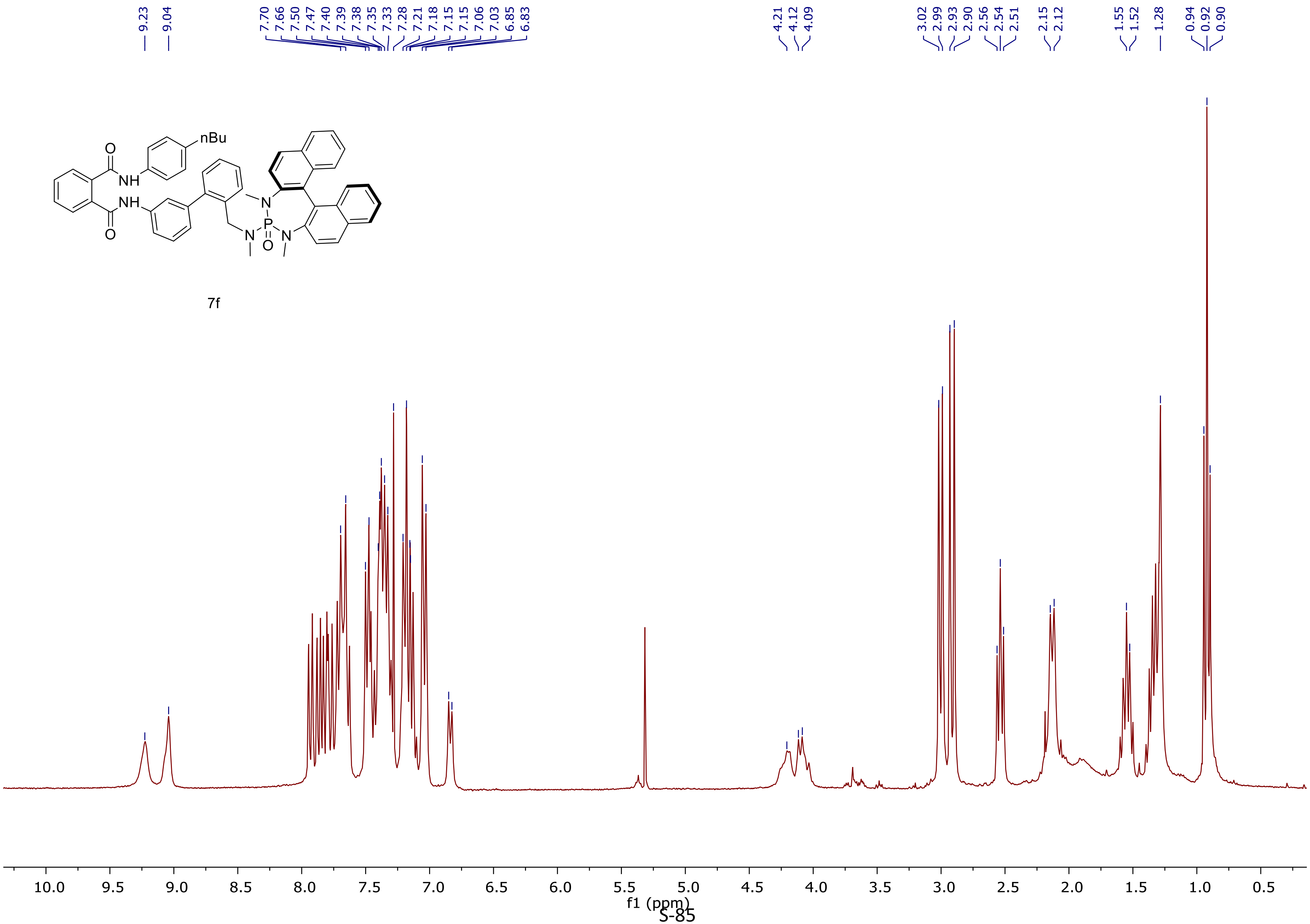
9.23
9.04
7.70
7.66
7.50
7.47
7.40
7.39
7.38
7.35
7.33
7.28
7.21
7.18
7.15
7.15
7.06
7.03
6.85
6.83
4.21
4.12
4.09
3.02
2.99
2.93
2.90
2.56
2.54
2.51
2.15
2.12
1.55
1.52
1.28
0.94
0.92
0.90
nBu
O
NH
NH
O
N
P
N
N
O
7f
10.0 9.5 9.0 8.5 8.0 7.5 7.0 6.5 6.0 5.5 5.0 4.5 4.0 3.5 3.0 2.5 2.0 1.5 1.0 0.5
f1 (ppm)

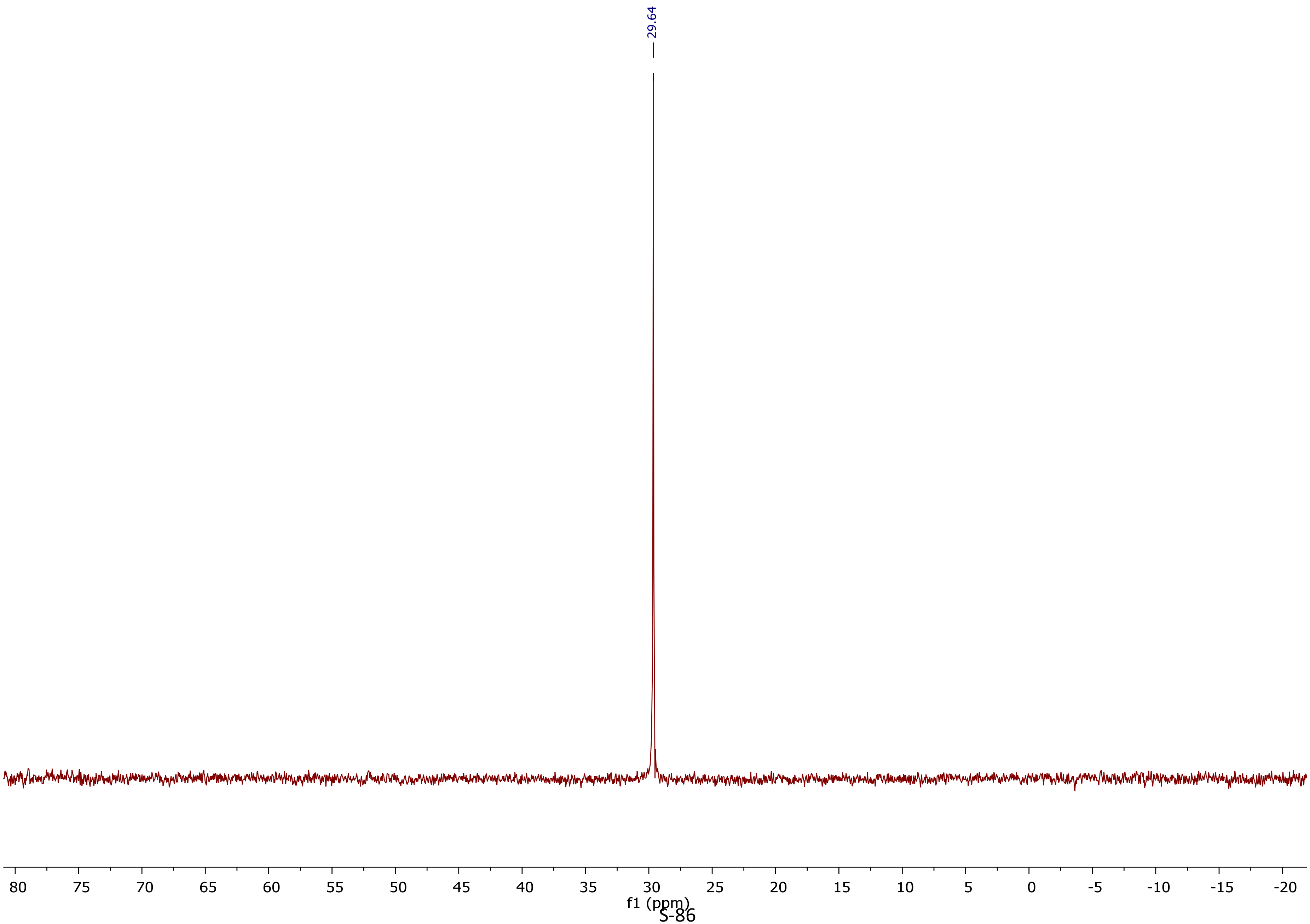
29.64
80 75 70 65 60 55 50 45 40 35 30 25 20 15 10 5 0 -5 -10 -15 -20
f1 (ppm)

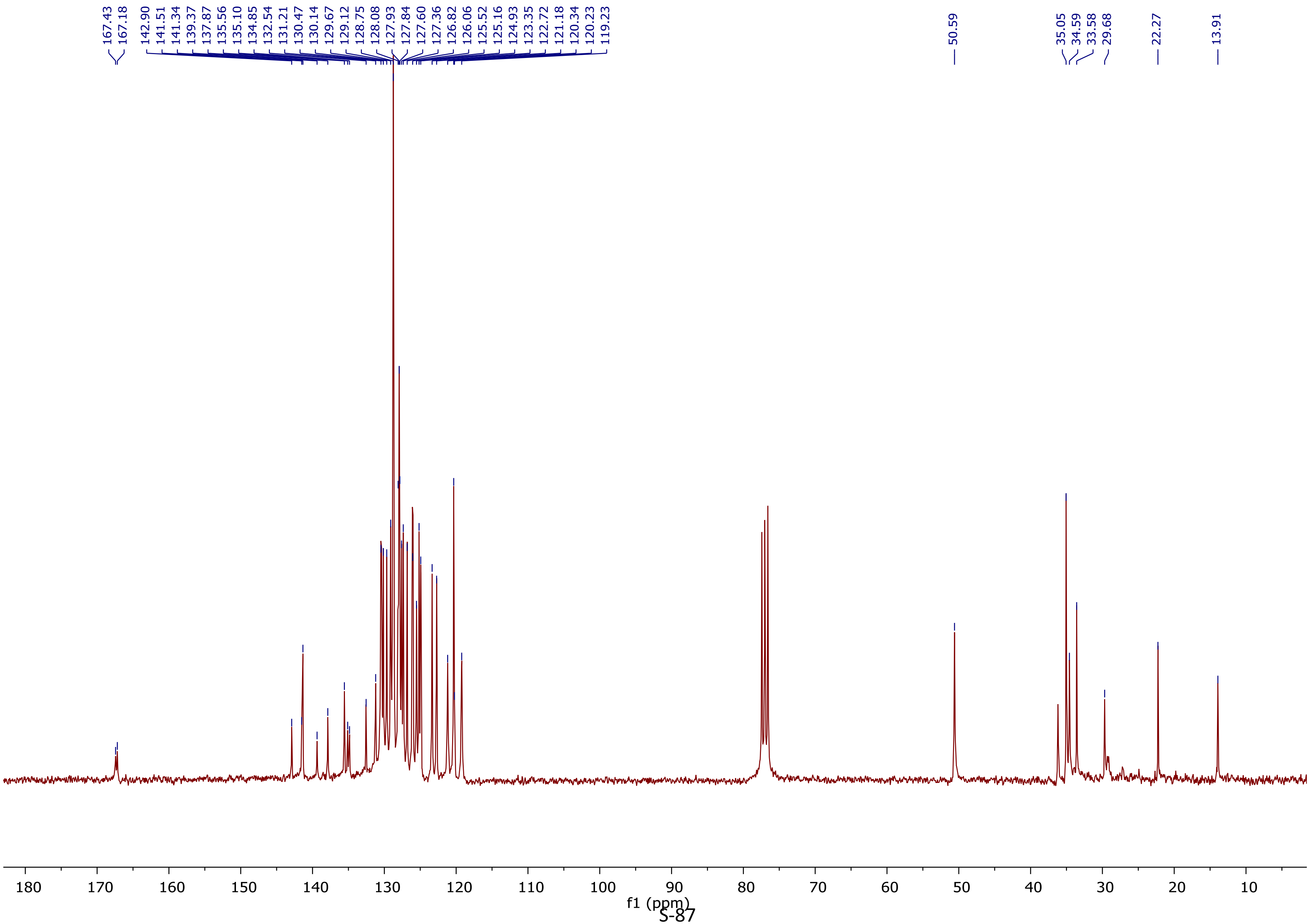
167.43
167.18
142.90
141.51
141.34
139.37
137.87
135.56
135.10
134.85
132.54
131.21
130.47
130.14
129.67
129.12
128.75
128.08
127.93
127.84
127.60
127.36
126.82
126.06
125.52
125.16
124.93
123.35
122.72
121.18
120.34
120.23
119.23
50.59
35.05
34.59
33.58
29.68
22.27
13.91
180
170
160
150
140
130
120
110
100
90
80
70
60
50
40
30
20
10
f1 (ppm)

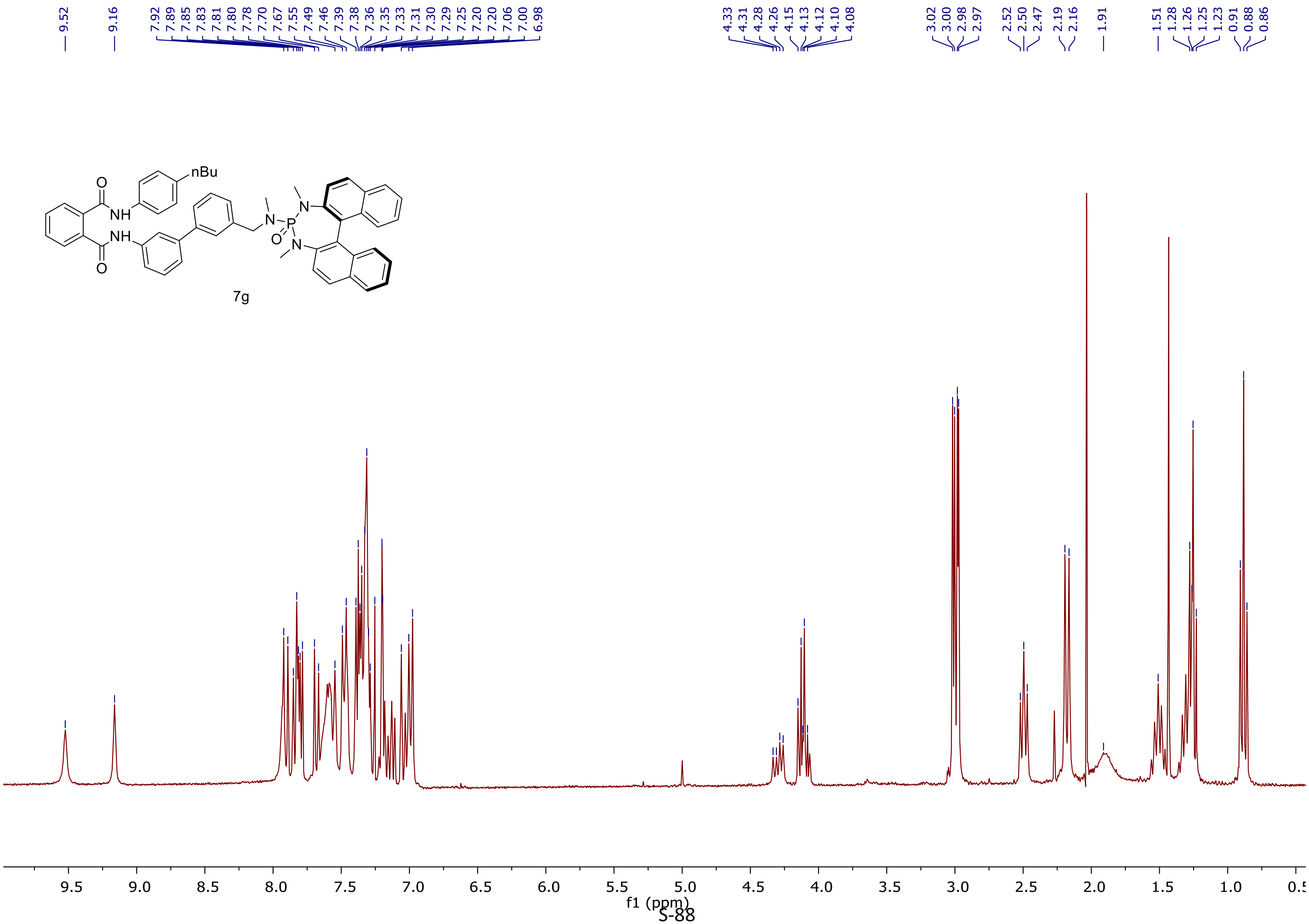
9.52
9.16
7.92 7.89 7.85 7.83 7.81 7.80 7.78 7.70 7.67 7.55 7.49 7.46 7.39 7.38 7.36 7.35 7.33 7.31 7.30 7.29 7.25 7.20 7.20 7.06 7.00 6.98
4.33 4.31 4.28 4.26 4.15 4.13 4.12 4.10 4.08
3.02 3.00 2.98 2.97
2.52 2.50 2.47
2.19 2.16
1.91
1.51 1.28 1.26 1.25 1.23
0.91 0.88 0.86
O
NH
nBu
NH
O
N
P
N
O
N
7g
9.5 9.0 8.5 8.0 7.5 7.0 6.5 6.0 5.5 5.0 4.5 4.0 3.5 3.0 2.5 2.0 1.5 1.0 0.5
f1 (ppm)

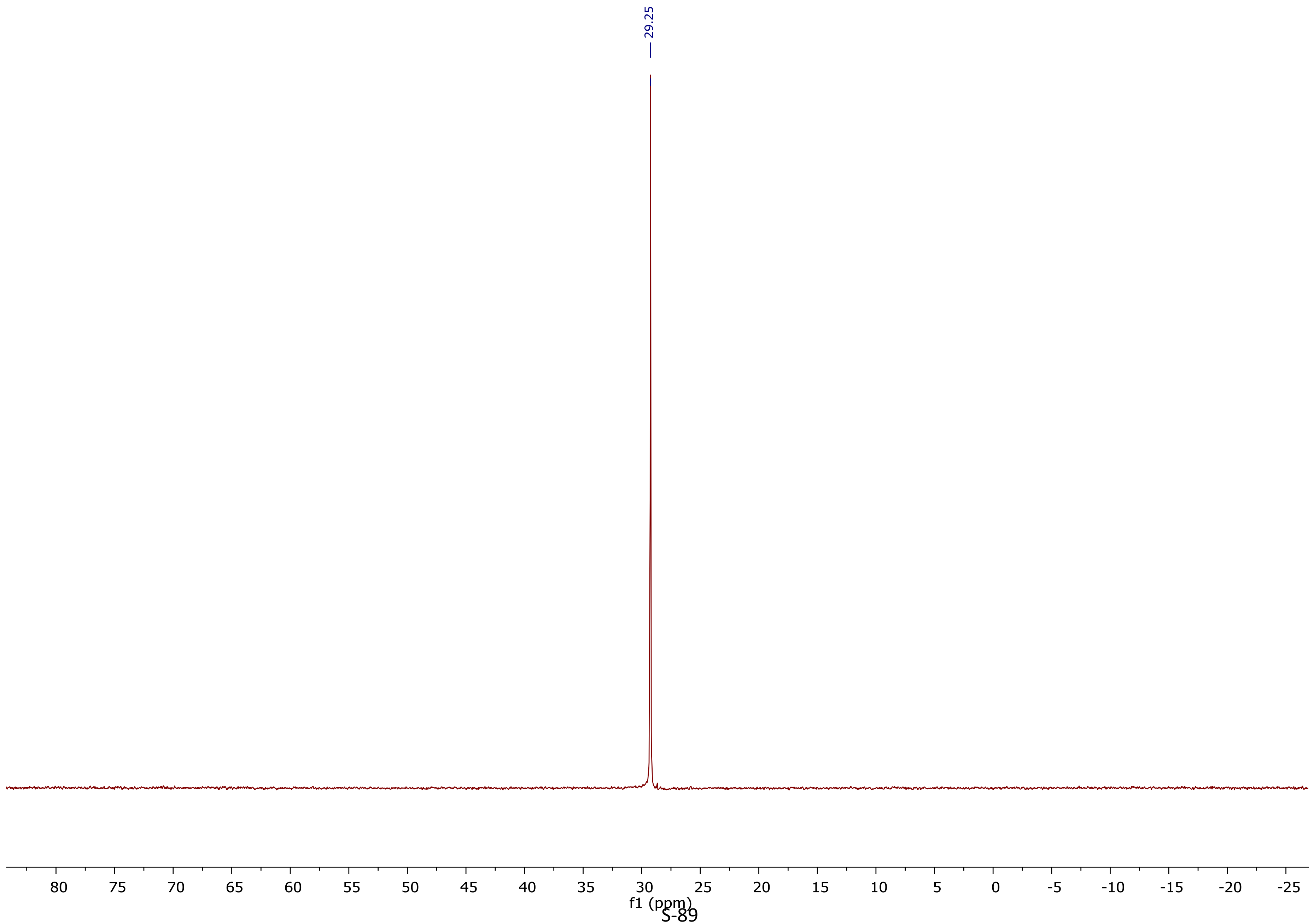
29.25
80
75
70
65
60
55
50
45
40
35
30
25
20
15
10
5
0
-5
-10
-15
-20
-25
f1 (ppm)

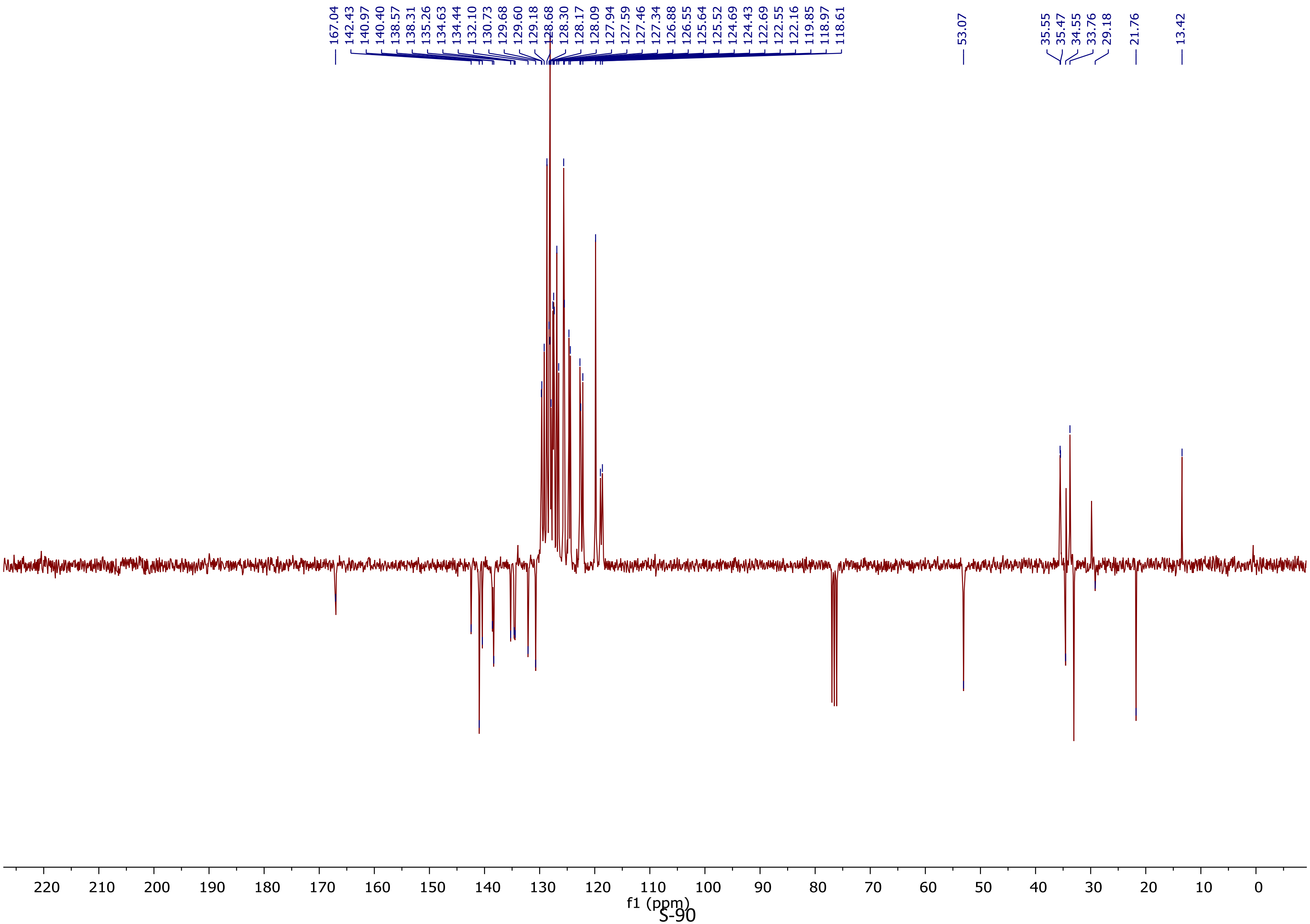
167.04
142.43
140.97
140.40
138.57
138.31
135.26
134.63
134.44
132.10
130.73
129.68
129.60
129.18
128.68
128.30
128.17
128.09
127.94
127.59
127.46
127.34
126.88
126.55
125.64
125.52
124.69
124.43
122.69
122.55
122.16
119.85
118.97
118.61
53.07
35.55
35.47
34.55
33.76
29.18
21.76
13.42
220 210 200 190 180 170 160 150 140 130 120 110 100 90 80 70 60 50 40 30 20 10 0
f1 (ppm)

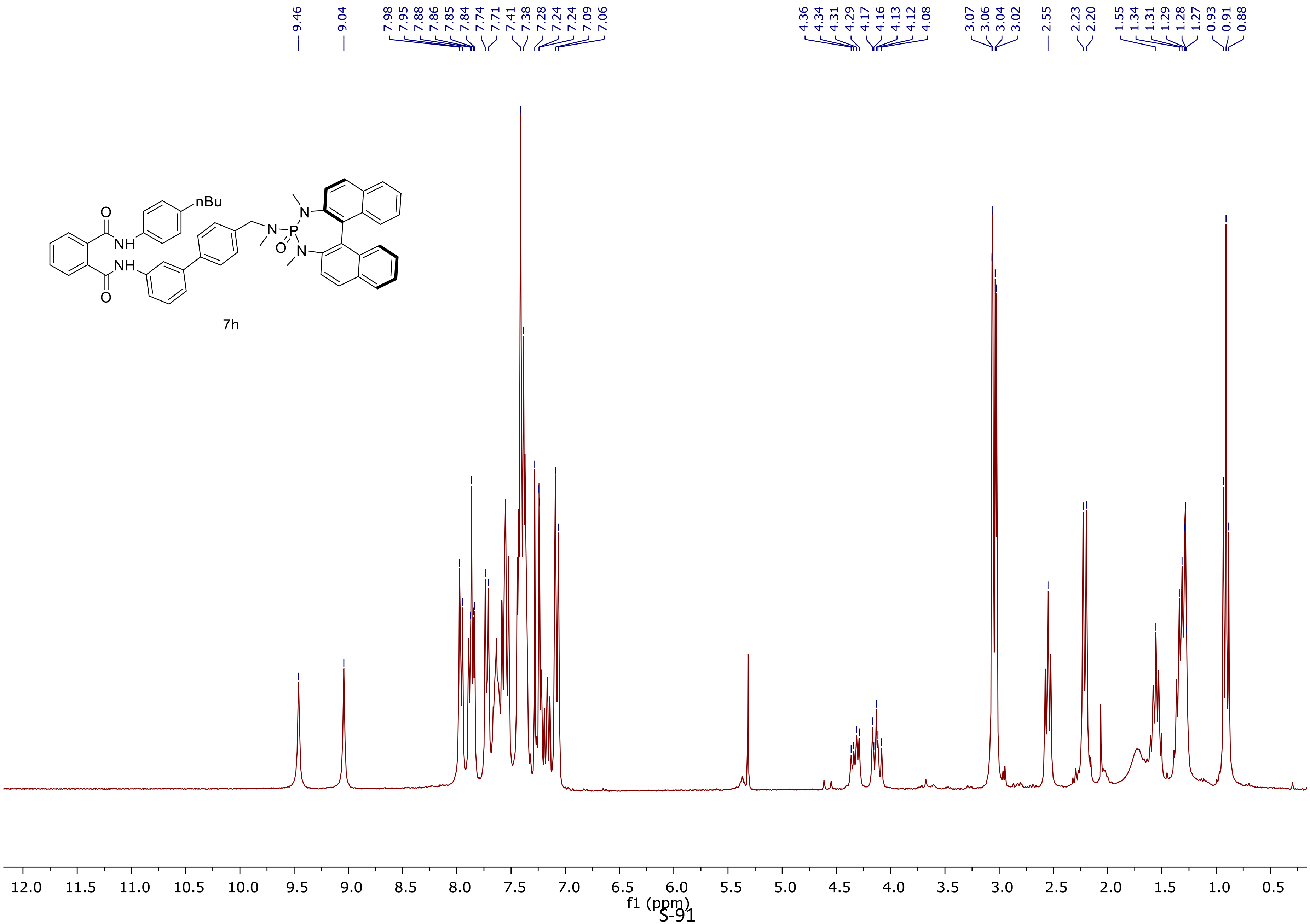

9.46
9.04
7.98
7.95
7.88
7.86
7.85
7.84
7.74
7.71
7.41
7.38
7.28
7.24
7.24
7.09
7.06
4.36
4.34
4.31
4.29
4.17
4.16
4.13
4.12
4.08
3.07
3.06
3.04
3.02
2.55
2.23
2.20
1.55
1.34
1.31
1.29
1.28
1.27
0.93
0.91
0.88
O
NH
nBu
NH
O
N
P
O
N
N
7h
12.0
11.5
11.0
10.5
10.0
9.5
9.0
8.5
8.0
7.5
7.0
6.5
6.0
5.5
5.0
4.5
4.0
3.5
3.0
2.5
2.0
1.5
1.0
0.5
f1 (ppm)

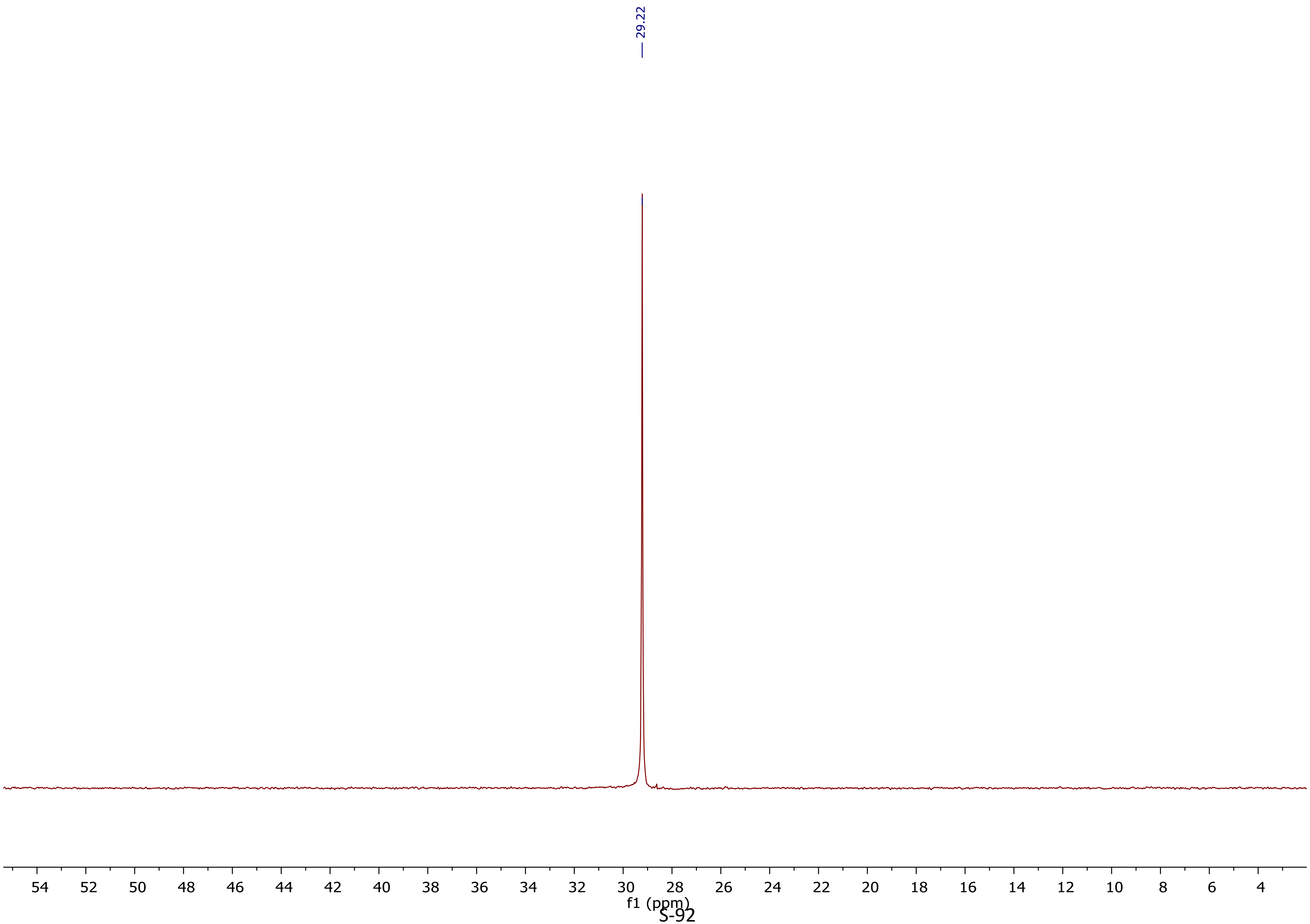
29.22
54
52
50
48
46
44
42
40
38
36
34
32
30
28
26
24
22
20
18
16
14
12
10
8
6
4
f1 (ppm)

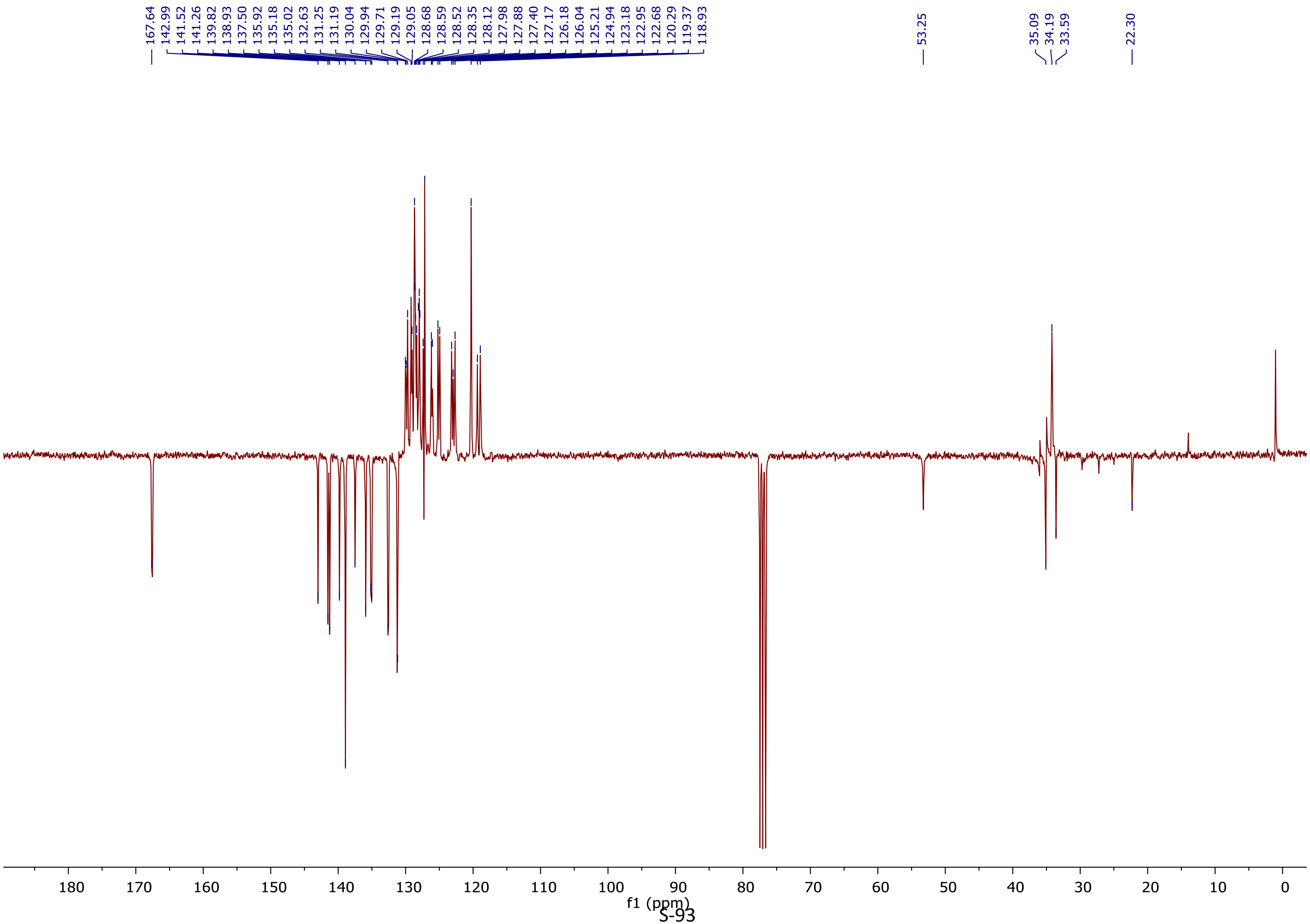

167.64
142.99
141.52
141.26
139.82
138.93
137.50
135.92
135.18
135.02
132.63
131.25
131.19
130.04
129.94
129.71
129.19
129.05
128.68
128.59
128.52
128.35
128.12
127.98
127.88
127.40
127.17
126.18
126.04
125.21
124.94
123.18
122.95
122.68
120.29
119.37
118.93
53.25
35.09
34.19
33.59
22.30
180 170 160 150 140 130 120 110 100 90 80 70 60 50 40 30 20 10 0
f1 (ppm)

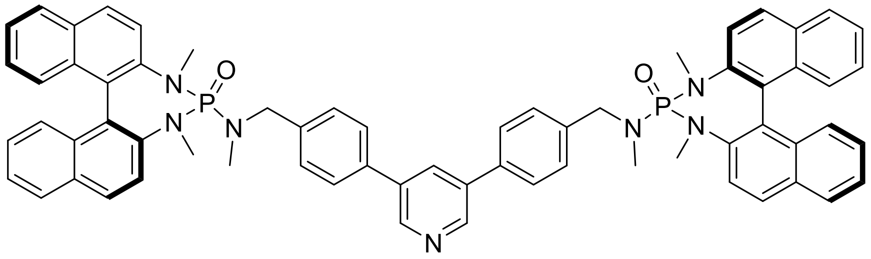

15a

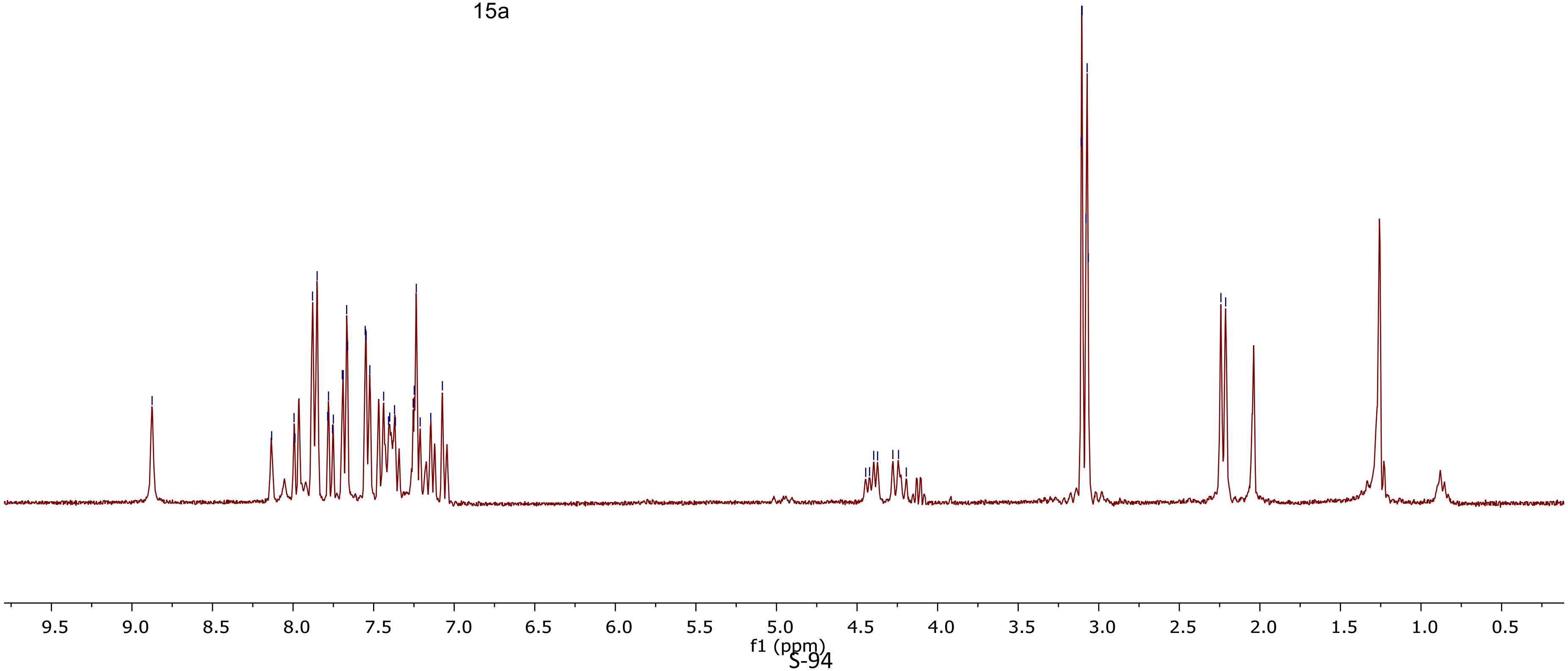

8.88
8.13
7.99
7.99
7.88
7.85
7.79
7.78
7.76
7.75
7.69
7.69
7.67
7.66
7.55
7.55
7.52
7.44
7.41
7.40
7.37
7.36
7.25
7.25
7.24
7.21
7.15
7.07
4.45
4.42
4.40
4.37
4.28
4.24
4.19
3.11
3.10
3.08
3.07
3.06
2.24
2.21
9.5
9.0
8.5
8.0
7.5
7.0
6.5
6.0
5.5
5.0
4.5
4.0
3.5
3.0
2.5
2.0
1.5
1.0
0.5
f1 (ppm)

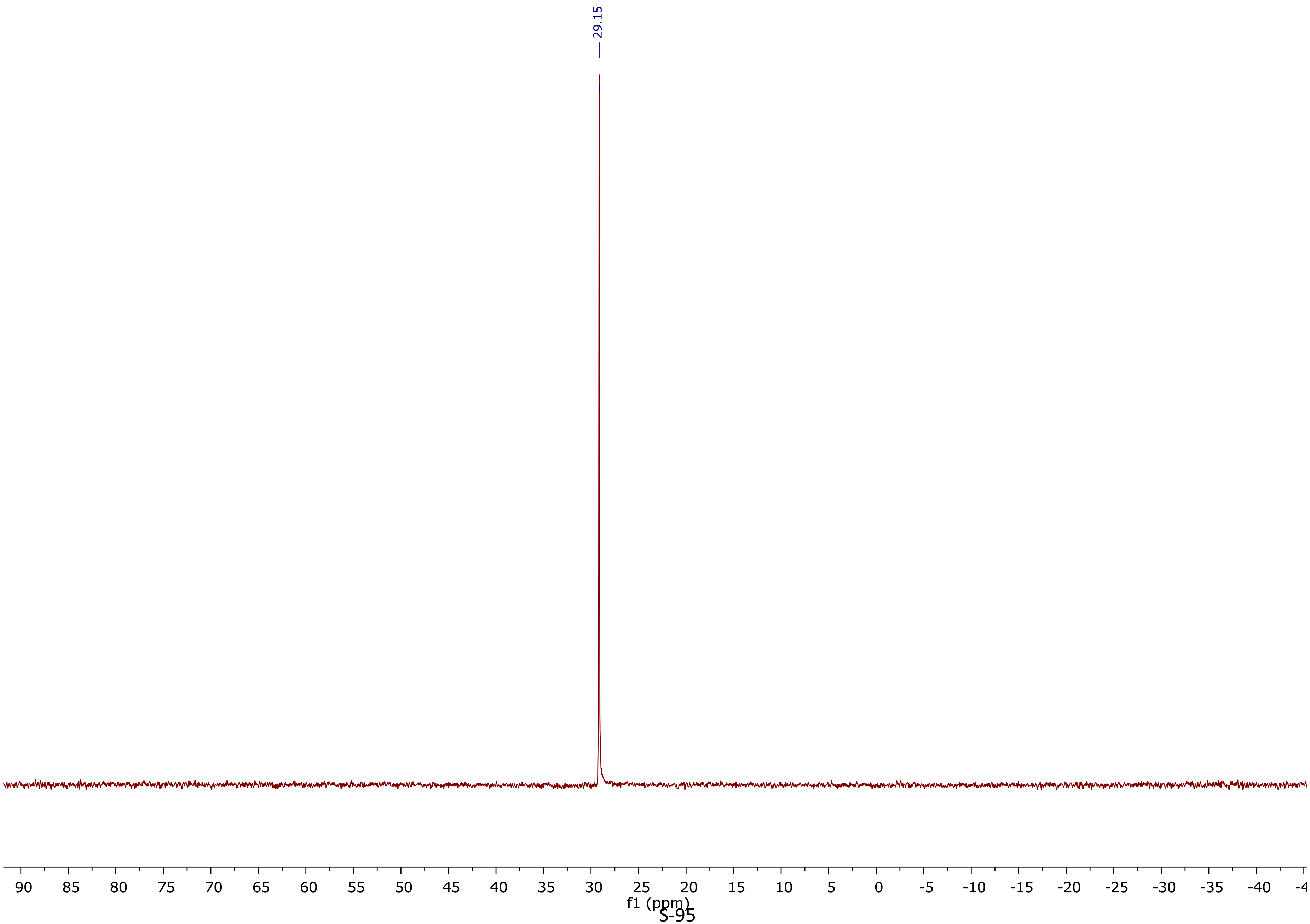
29.15
f1 (ppm)

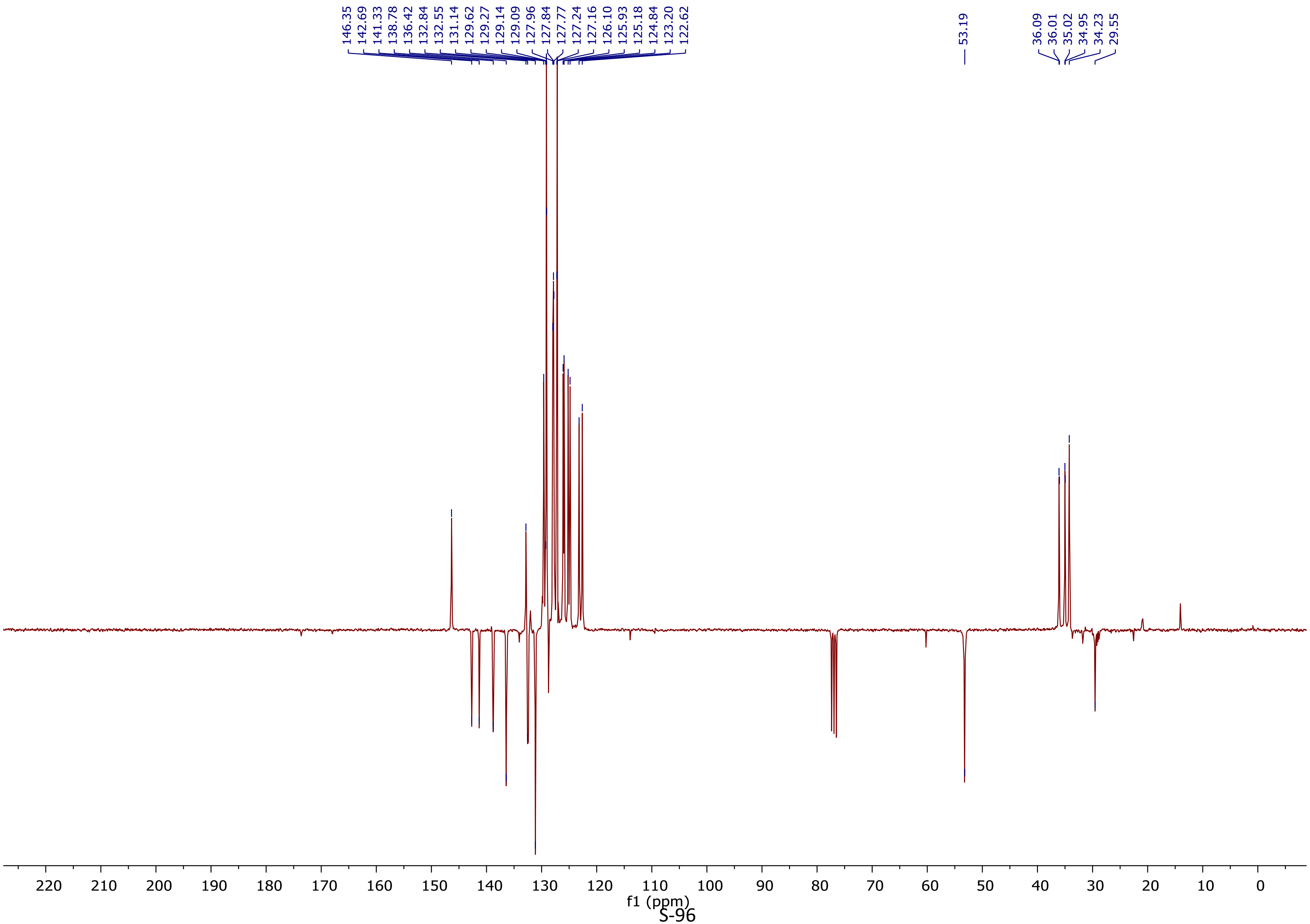
146.35
142.69
141.33
138.78
136.42
132.84
132.55
131.14
129.62
129.27
129.14
129.09
127.96
127.84
127.77
127.24
127.16
126.10
125.93
125.18
124.84
123.20
122.62
53.19
36.09
36.01
35.02
34.95
34.23
29.55
220 210 200 190 180 170 160 150 140 130 120 110 100 90 80 70 60 50 40 30 20 10 0
f1 (ppm)

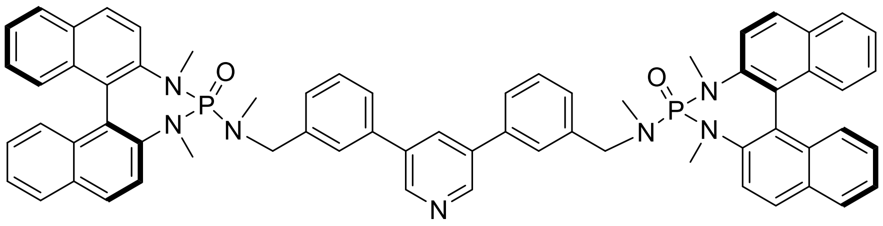

15b

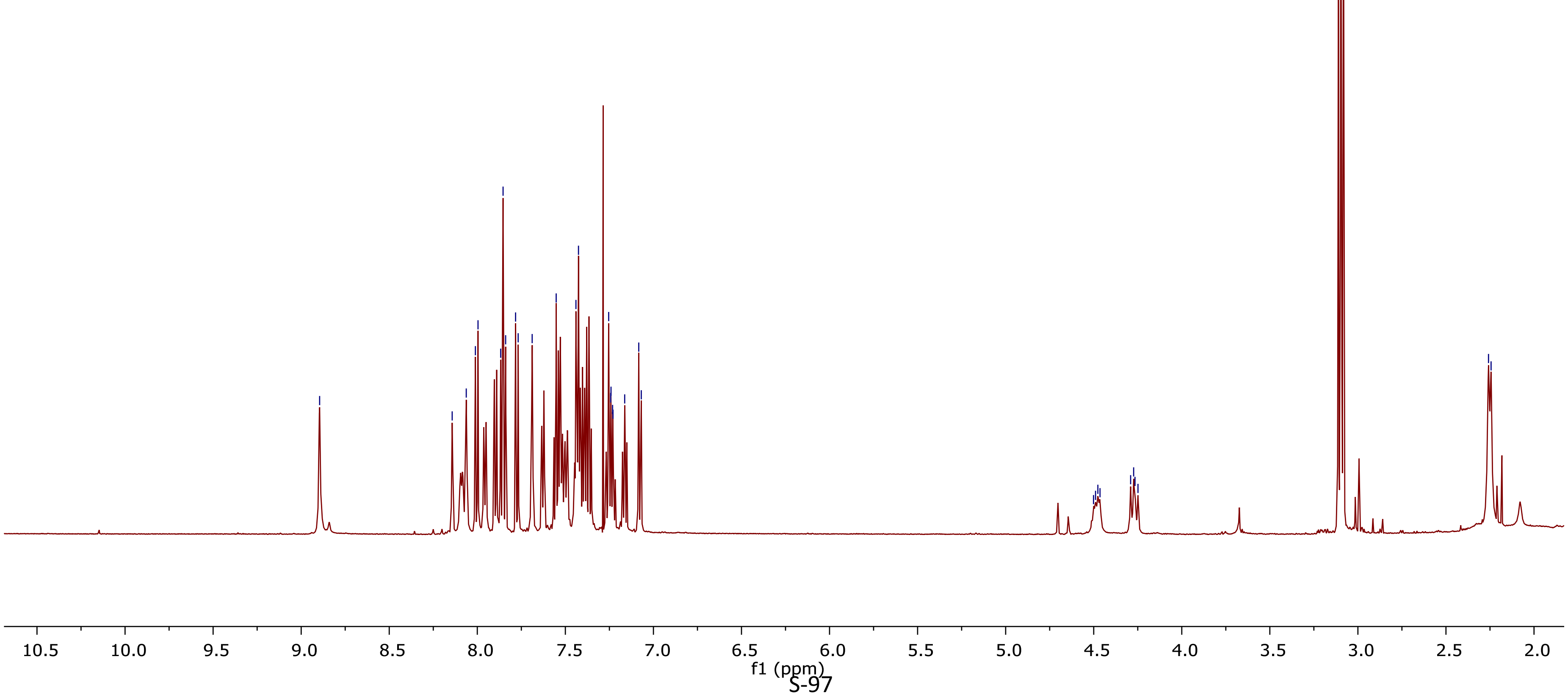

8.90
8.14 8.06 8.01 8.00 7.87 7.85 7.84 7.78 7.77 7.69 7.55 7.44 7.43 7.25 7.24 7.24 7.23 7.23 7.16 7.08 7.07
4.50 4.49 4.48 4.46 4.29 4.27 4.27 4.25
3.11 3.10 3.09 3.08
2.26 2.24
10.5 10.0 9.5 9.0 8.5 8.0 7.5 7.0 6.5 6.0 5.5 5.0 4.5 4.0 3.5 3.0 2.5 2.0
f1 (ppm)

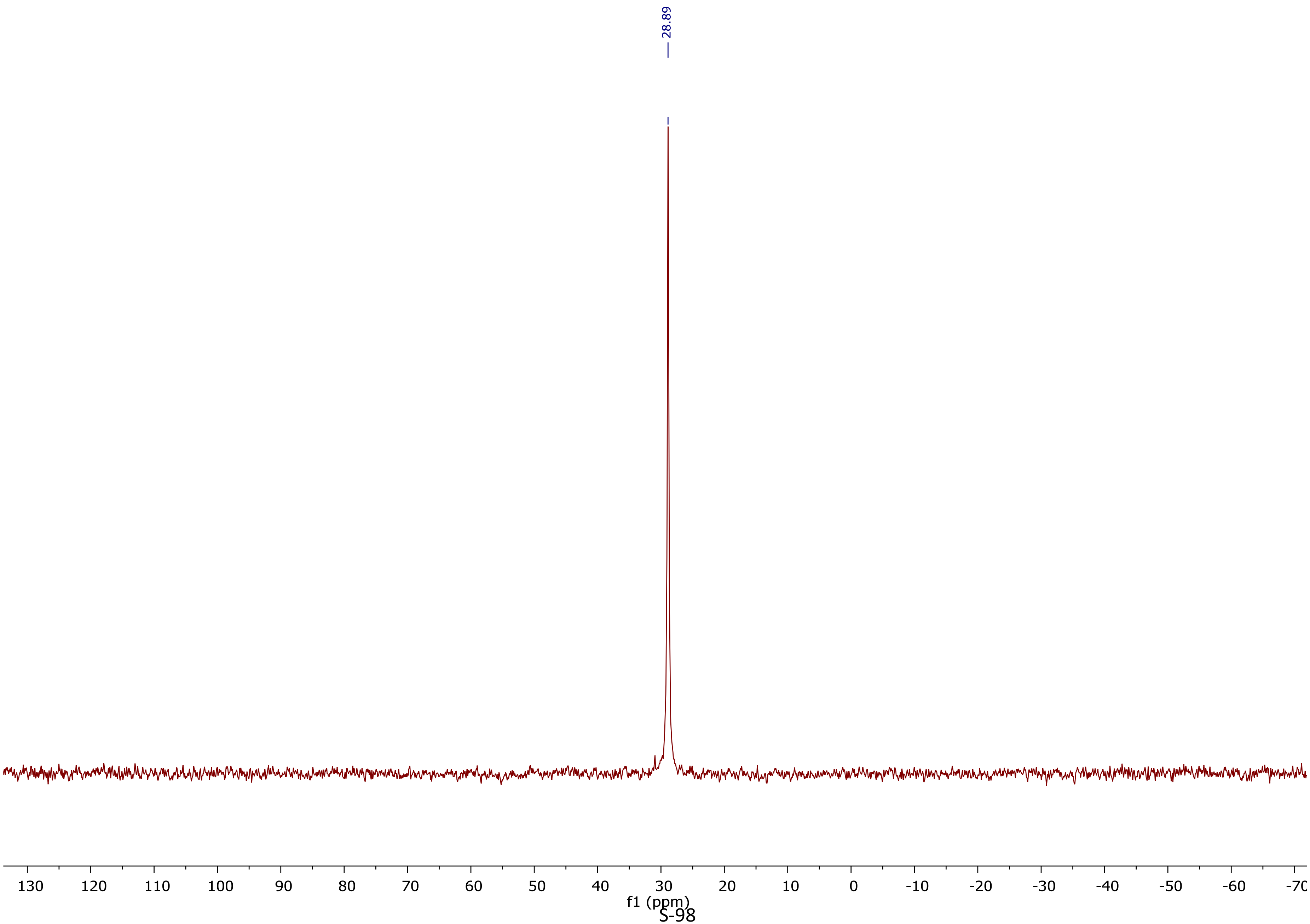
28.89
130 120 110 100 90 80 70 60 50 40 30 20 10 0 -10 -20 -30 -40 -50 -60 -70
f1 (ppm)

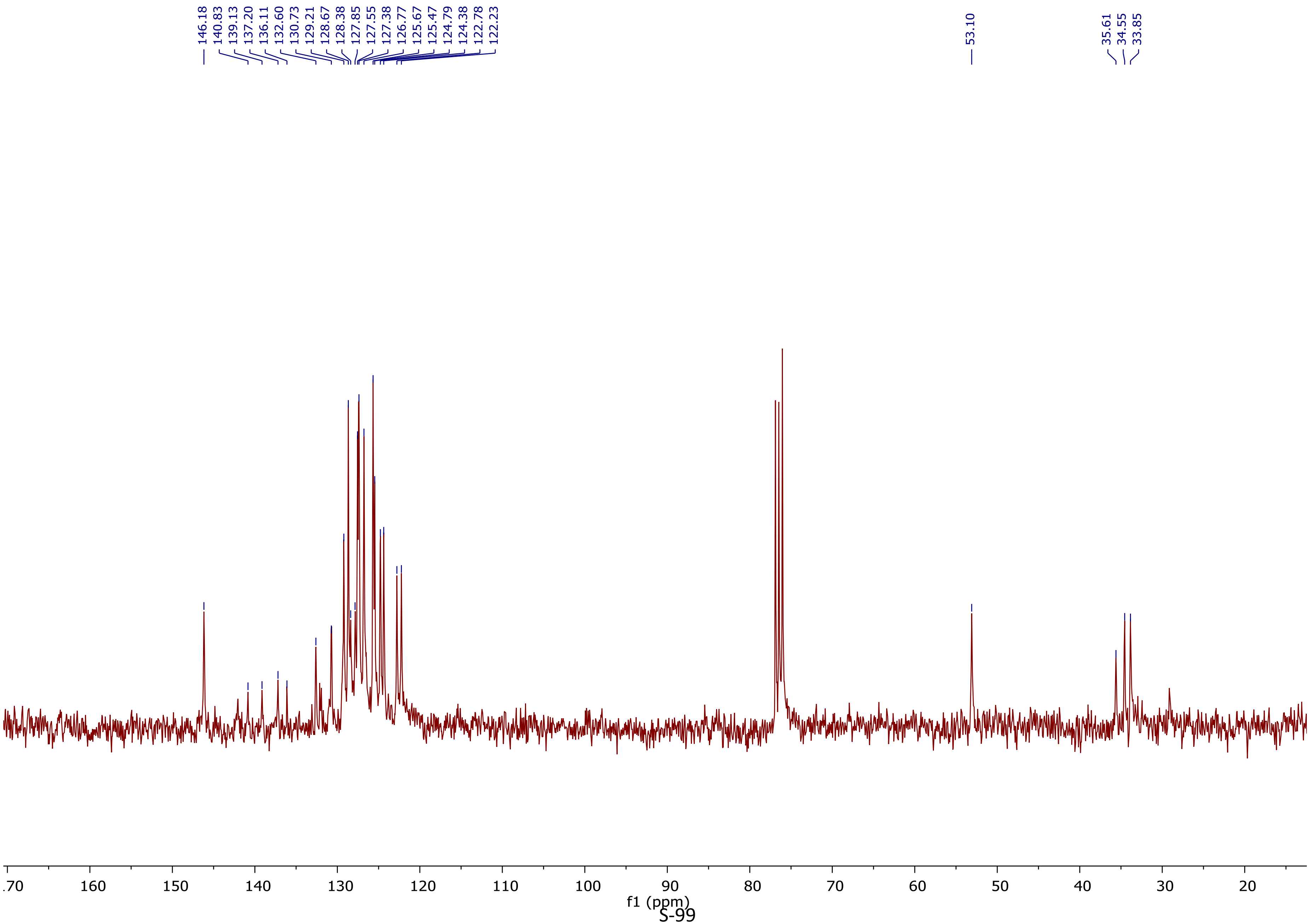

146.18
140.83
139.13
137.20
136.11
132.60
130.73
129.21
128.67
128.38
127.85
127.55
127.38
126.77
125.67
125.47
124.79
124.38
122.78
122.23
53.10
35.61
34.55
33.85
.70
160
150
140
130
120
110
100
90
80
70
60
50
40
30
20
f1 (ppm)
